# Mechanism and selectivity of the dinuclear iron benzoyl-coenzyme A epoxidase BoxB†
†Electronic supplementary information (ESI) available. See DOI: 10.1039/c5sc00313j



**DOI:** 10.1039/c5sc00313j

**Published:** 2015-03-02

**Authors:** Rong-Zhen Liao, Per E. M. Siegbahn

**Affiliations:** a Key Laboratory for Large-Format Battery Materials and System , Ministry of Education , School of Chemistry and Chemical Engineering , Huazhong University of Science and Technology , Wuhan 430074 , China . Email: rongzhen@hust.edu.cn; b Department of Organic Chemistry , Arrhenius Laboratory , Stockholm University , SE-10691 Stockholm , Sweden . Email: ps@organ.su.se

## Abstract

Benzoyl-CoA epoxidase is a dinuclear iron enzyme that catalyzes the epoxidation reaction of the aromatic ring of benzoyl-CoA with chemo-, regio- and stereo-selectivity. It has been suggested that this enzyme may also catalyze the deoxygenation reaction of epoxide, suggesting a unique bifunctionality among the diiron enzymes. We report a density functional theory study of this enzyme aimed at elucidating its mechanism and the various selectivities. The epoxidation is suggested to start with the binding of the O_2_ molecule to the diferrous center to generate a diferric peroxide complex, followed by concerted O–O bond cleavage and epoxide formation. Two different pathways have been located, leading to (2*S*,3*R*)-epoxy and (2*R*,3*S*)-epoxy products, with barriers of 17.6 and 20.4 kcal mol^–1^, respectively. The barrier difference is 2.8 kcal mol^–1^, corresponding to a diastereomeric excess of about 99 : 1. Further isomerization from epoxide to phenol is found to have quite a high barrier, which cannot compete with the product release step. After product release into solution, fast epoxide–oxepin isomerization and racemization can take place easily, leading to a racemic mixture of (2*S*,3*R*) and (2*R*,3*S*) products. The deoxygenation of epoxide to regenerate benzoyl-CoA by a diferrous form of the enzyme proceeds *via* a stepwise mechanism. The C2–O bond cleavage happens first, coupled with one electron transfer from one iron center to the substrate, to form a radical intermediate, which is followed by the second C3–O bond cleavage. The first step is rate-limiting with a barrier of only 10.8 kcal mol^–1^. Further experimental studies are encouraged to verify our results.

## Abstract 2

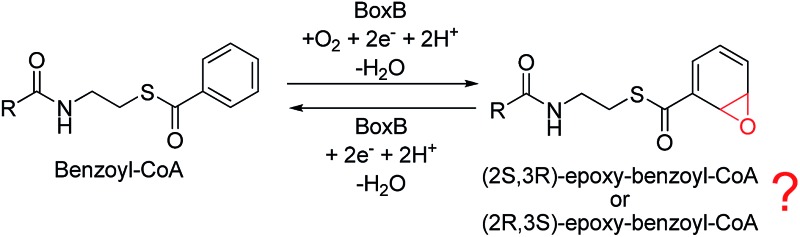
DFT calculations are used to elucidate the reaction mechanism and selectivity of BoxB catalyzed benzoyl-CoA epoxidation.

## 1. Introduction

Aromatic organic compounds like benzoate and phenylacetate represent the second most abundant class of nutrients in nature after carbohydrates. The metabolism of these compounds is dominated by microorganisms and therefore they play an important role in the biogeochemical carbon cycle and bioremediation of contaminated ecosystems.^1,2^ To overcome the high resonance energy of the aromatic ring, three different catabolic strategies are used by microorganisms to activate and cleave the conjugated cyclic ring. Under aerobic conditions, molecular oxygen is utilized as a co-substrate to oxidize and cleave the ring.^3–7^ Under anaerobic conditions, the metabolism proceeds *via* initial conversion to aromatic coenzyme A derivatives, which can undergo reductive dearomatization and then hydrolytic ring cleavage.^8–12^ The third strategy used by many bacteria is to combine the first aerobic and the second anaerobic approaches to adapt to a low or fluctuating O_2_ concentration.^13–16^ The pathway starts with the formation of CoA derivatives, which are then used as substrates for oxygen-dependent epoxidation. Two members have been discovered and characterized to affect the epoxidation, namely benzoyl-CoA epoxidase (BoxB)^17^ and phenylacetyl-CoA epoxidase (PaaAC).^18^

**Fig. 1:**
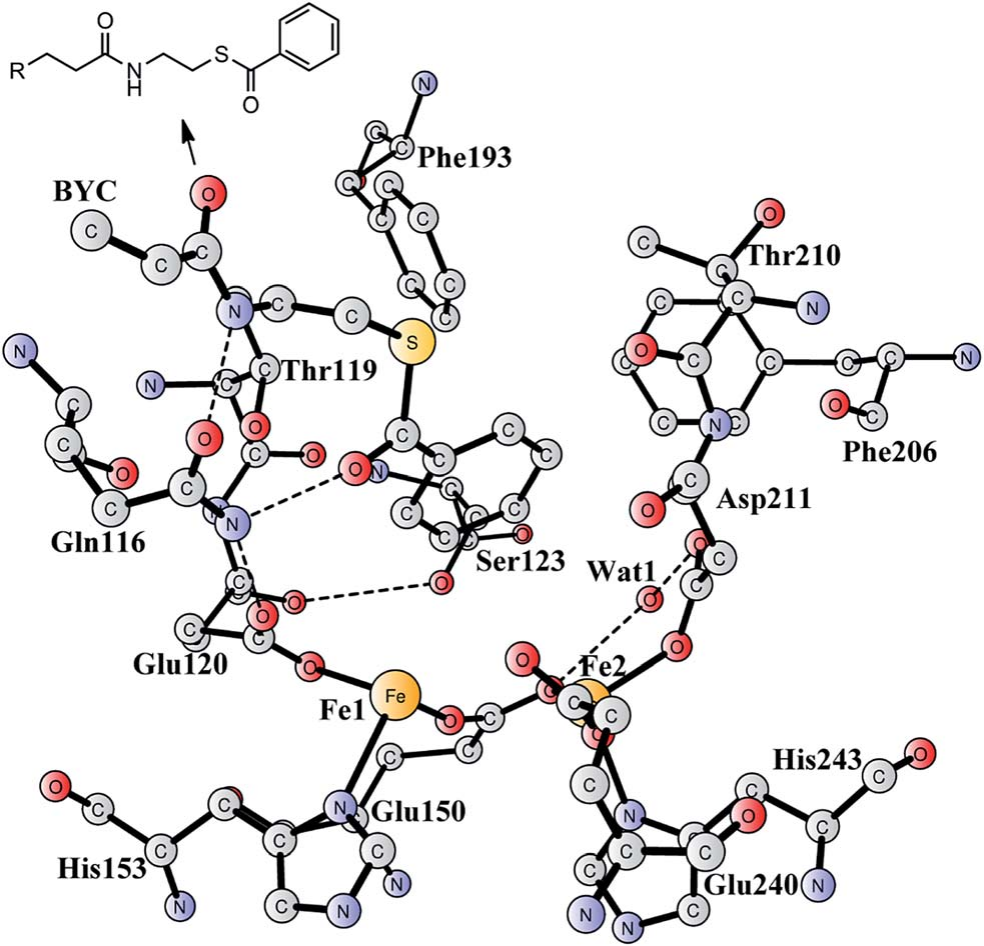
X-ray structure of the active site of BoxB from *Azoarcus evansii* complexed with benzoyl-CoA (coordinates taken from PDB entry ; 3PM5).^17^ The residue name of the benzoyl-CoA substrate is BYC.

BoxB was first demonstrated to be a member of the diiron protein family through amino acid sequence comparisons.^19^ This family is a group of enzymes that possess a common O_2_ activation mechanism but enable diverse oxygen-dependent chemical transformations, such as hydroxylation,^20–22^ amide oxidation,^23^ decarbonylation,^24^ double bond formation,^25^ C–P bond cleavage,^26^ radical generation,^27^ and epoxidation.^17,18^ The X-ray crystal structure of BoxB from *Azoarcus evansii* (resolution of 2.3 Å) has been solved in complex with the benzoyl-CoA substrate and reveals a reduced diferrous site in the active site.^17^ The two iron ions are bridged by a glutamate (Glu150, see Fig. 1). Fe1 is ligated by a glutamate (Glu120) and a histidine (His153), while Fe2 is ligated by an aspartate (Asp211), a glutamate (Glu240) and a histidine (His243). In addition, a water molecule is seen to form a hydrogen bond with Glu150 and Asp211. Furthermore, the benzoyl-CoA substrate forms two hydrogen bonds with Gln116, which in turn is hydrogen-bonded to Glu120. A number of other second-shell residues are also important for the orientation of the benzoyl moiety, including Thr119, Ser123, Phe193, Phe203, and Thr210.

On the basis of the X-ray structure^17^ and earlier ^18^O isotope labeling studies,^15^ the product for this enzyme has been proposed to be (2*S*,3*R*)-epoxy-benzoyl-CoA, rather than the tautomeric oxepin with an opening of the epoxide ring. During the reaction, no aromatic mono-hydroxylation^28–36^ or di-hydroxylation^37,38^ takes place. In addition, the epoxide is formed exclusively at the 2,3-position, rather than the 1,2-position or 3,4-position.^17^ Finally, for the generation of a 2,3-epoxide, the configuration can be either (2*S*,3*R*) or (2*R*,3*S*), and the (2*S*,3*R*) seems to be more likely.^17^ Nevertheless, further experiments are needed to assign the product configuration and measure the stereoselectivity. These findings suggest a unique feature of this enzyme, which shows chemoselectivity, regioselectivity, and stereoselectivity. A plausible reaction mechanism has been put forward to explain the reactivity of this enzyme.^17^ The resting state is a diferric complex with two bridging hydroxides. Upon reduction by BoxA/NADPH, two water molecules are released into solution and the benzoyl-CoA substrate enters into the active site. This is followed by O_2_ binding to the diiron site first to form an end-on superoxide complex, which is then converted into a bis-μ-oxo-bridged diferryl intermediate similarly to the extensively-studied methane monooxygenase (MMO).^20^ Subsequently, one terminal oxo group attacks the C2 and C3 atoms of the phenyl ring in either a concerted or stepwise pathway. Finally, the epoxide product is released and the reaction with a water molecule can generate the starting resting species.

It should be mentioned that PaaAC catalyzes the epoxide formation at the 1,2-position, which is different from BoxB.^18^ The crystal structure of PaaAC has also been reported with conserved first-shell ligands (three Glu, two His, and one Asp) but only in an apo-form without iron ions in the diiron active site.^18^ Interestingly, PaaAC is able to mediate the removal of the epoxide oxygen with the formation of a water molecule by using NADPH as the electron donor.^39^ The bifunctionality of this enzyme has been proposed to play an important role in controlling the toxic epoxide concentration. It was speculated that BoxB may have a similar bifunctionality.^39^ This makes BoxB significantly different from other types of diiron enzymes.

In the present work, the quantum chemical cluster approach^40–45^ was used to investigate the reaction mechanism and selectivity of BoxB. With a quite large model of the active site designed on the basis of the crystal structure (PDB entry: ; 3PM5), density functional calculations were performed to calculate the potential energy profile for the epoxidation and also the deoxygenation reaction catalyzed by this enzyme. This kind of methodology has been successfully applied to the study of various classes of enzymes,^40–45^ including several related diiron enzymes.^46–56^

## 2. Computational details

A model of the BoxB active site was designed on the basis of a crystal structure of the wild-type BoxB in complex with the benzoyl-CoA substrate (PDB entry 3PM5).^17^ The model consists of the two iron ions along with their ligands Glu120, Glu150, His153, Asp211, His243, and His 240. In addition, a water molecule was added to coordinate to Fe1, which has been found for other di-iron enzymes.^46–56^ Furthermore, six important second-shell residues, Gln116, Thr119, Ser123, Phe193, Phe206, Thr210, and a water molecule (Wat1 in Fig. 1), which might be involved in controlling the orientation of the substrate benzoyl group, were also included in the model. Hydrogen atoms were added manually, and the amino acids were truncated at their α-carbon so that in principle only side chains were kept in the model. However, the two peptide chains of Thr119-Glu120 and Thr210-Asp211 were included. The substrate was truncated with two carbons next to the amide carbonyl carbon to allow enough flexibility of the aromatic ring. Truncated bonds were saturated with hydrogen atoms. For the epoxidation reaction, an oxygen molecule was added to bind to the diiron center. The model is thus composed of 208 atoms and has a total charge of 0.

The quantum chemical calculations presented herein were accomplished with the B3LYP^57^ functional as implemented in the Gaussian 09 program.^58^ For geometry optimization, the 6-31G(d,p) basis sets were used for the C, N, O, S, and H elements and the LANL2DZ^59^ pseudopotential for Fe. The anti-ferromagnetically coupled singlet (high spin on each Fe) state was considered for all calculations, similarly to other diiron enzymes that have been studied before.^46–56^ On the basis of these optimized geometries, single-point calculations were performed at the B3LYP*^60^ level using larger basis sets, in which Fe was described by the LANL2TZ(f)^61^ basis sets (with pseudopotential) and the 6-311+G(2d,2p) for all other elements. It has been shown that B3LYP* performs better in describing relative energies in transition metal complexes.^60^ D3 dispersion corrections (with the original D3 damping function) proposed by Grimme^62^ were also added at single-points.

To estimate the polarization effects from the protein environment on the active site model, single-point calculations were carried out at the same level of theory as the geometry optimizations using the SMD^63^ solvation model method. The dielectric constant was chosen to be 4, which is the standard value used for the modeling of the enzyme surroundings. In the present case, the solvation effect is quite small, in the range of 0–2 kcal mol^–1^. In fact, it has been shown for several different classes of enzymes that the solvation effects vanish rapidly when the size of the active site model reaches 150–200 atoms.^64–67^

Analytic frequency calculations were performed at the same level of theory as the geometry optimizations to obtain zero-point energies (ZPE) and to establish the nature of the various stationary points. As discussed below, some atoms were kept fixed to their X-ray crystal structure positions during the geometry optimizations (marked with asterisks), to mimic the steric constraints imposed by the protein matrix. This coordinate locking scheme introduces a few small imaginary frequencies, usually on the order of 10–50i cm^–1^. These frequencies do not contribute significantly to the ZPE and thus can be disregarded. Here, the B3LYP*-D3 energies, including solvation, ZPE, and dispersion corrections from B3LYP are reported.

## 3. Results and discussion

In this section, we first establish the epoxidation mechanism and rationalize the underlying selectivity (Section 3.1). Next, the interconversion of the epoxide product and the oxepin product (Section 3.2) and the conversion of the epoxide product to the phenol product (Section 3.3) in both enzyme and water solution are presented. Finally, we present the deoxygenation reaction of the epoxide product (Section 3.4).

### 3.1 Epoxidation of benzoyl-CoA

**Fig. 2:**
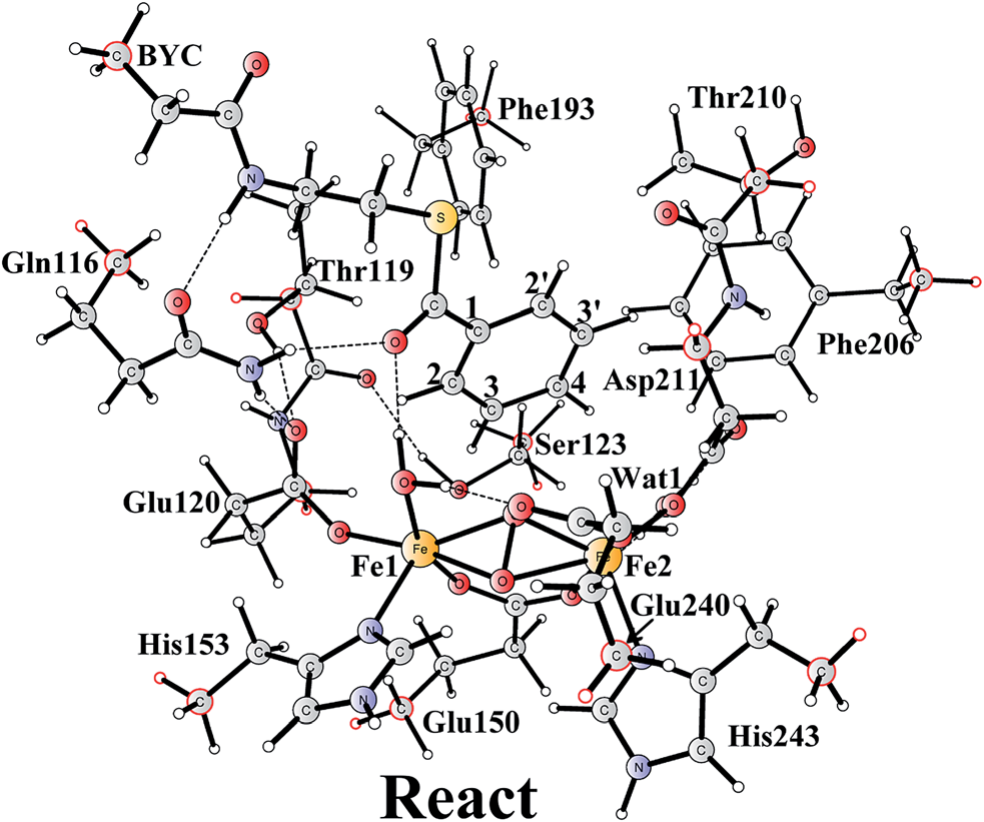
Optimized structure of the active site model of BoxB. Atoms marked with red were fixed at their X-ray structure positions during the geometry optimizations.

The optimized structure of the BoxB active site with the truncated benzoyl-CoA and O_2_ substrates bound, corresponding to the Michaelis complex (**React**), is shown in Fig. 2 (full model) and 3 (core part of the full model). Four different isomers (labeled as A, B, C, and D) have been located, depending on the coordination mode of the O_2_ moiety. In **React_A_**, O_2_ binds to the two metal sites in a side-on symmetric fashion, and this mode has the lowest energy, similarly to MMO.^47–53^ The Mulliken spin densities on Fe1 and Fe2 are 4.03 and –4.01, respectively, and the spin densities on the O_2_ moiety are quite small. These results suggest that during the O_2_ binding each Fe(ii) ion donates one electron to the O_2_ moiety to generate a peroxide (O_2_^2–^) and that the electronic structure of **React_A_** (open-shell singlet) can be interpreted as featuring an antiferromagnetic coupling between two high-spin ferric ions (*S* = 5/2). The binding of O_2_ to the active site has also been considered, which was calculated to be slightly endergonic by 2.2 kcal mol^–1^ including an empirical free energy (entropy) loss of 10 kcal mol^–1^. Similar results have been found for ribonucleotide reductase (RNR) with FeFe, FeMn, and MnMn sites, in which the binding of O_2_ was calculated to be close to thermoneutral.^50^ The energy cost for the binding of O_2_ is quite small, and since the error of calculating such a process is somewhat larger than the following reactions, this energy cost is not included in the energy diagram and the energy of **React_A_** is set to zero.

**Fig. 3:**
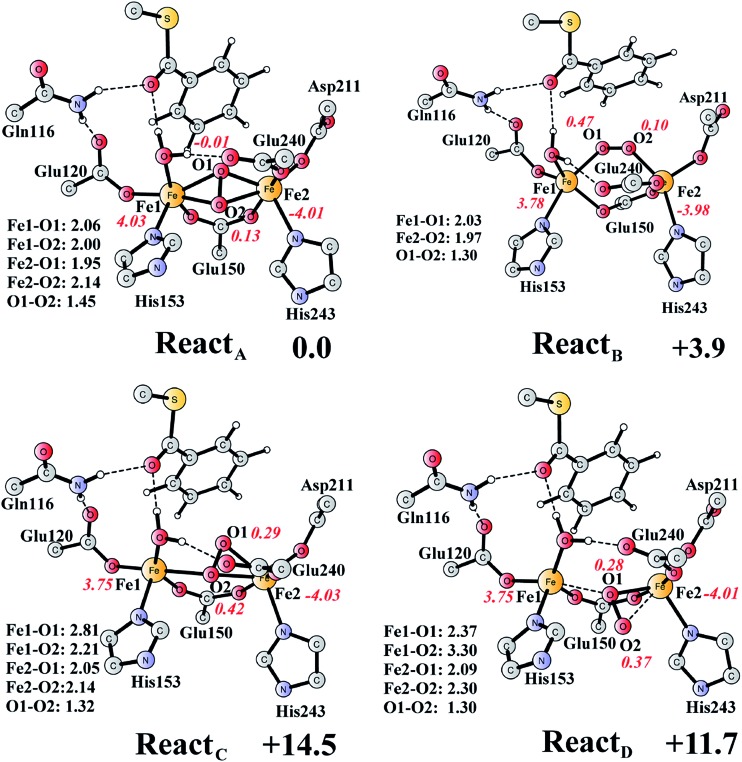
Optimized structures of the four different isomers of the reactant complex (**React**). Energies are given in kcal mol^–1^ relative to **React_A_**. Distances are given in Ångstrom, and Mulliken spin densities are shown in red italic. For clarity, only the core of the model is shown. For full model, see Fig. 2.

**React_B_**, with O_2_ in an end-on binding mode, is 3.9 kcal mol^–1^ higher than **React_A_**. Significant spin densities can be seen on the O_2_ moiety (0.57 in total), and the electronic structure of **React_B_** can be described as a high-spin ferrous ion (Fe1, *S* = 2) ferromagnetically coupled to the superoxide (O_2_^–^, *S* = 1/2) and antiferromagnetically coupled to a high-spin ferric ion (Fe2, *S* = 5/2). In the other two isomers (**React_C_** and **React_D_**), the O_2_ moiety binds in a side-on asymmetric manner, which is energetically unfavorable with energies that are more than 10 kcal mol^–1^ higher. This is different from the FeMn RNR, in which the side-on asymmetric complex is slightly favored compared with the symmetric one, and also different from the MnMn RNR, in which the end-on binding mode is preferred.^51^ It should be pointed out that the diiron myo-inositol oxygenase adopts an O_2_ binding mode that is different from these discussed above, and O_2_ coordinates to a ferrous ion in a side-on manner to generate a ferric superoxo.^68^

**Fig. 4:**
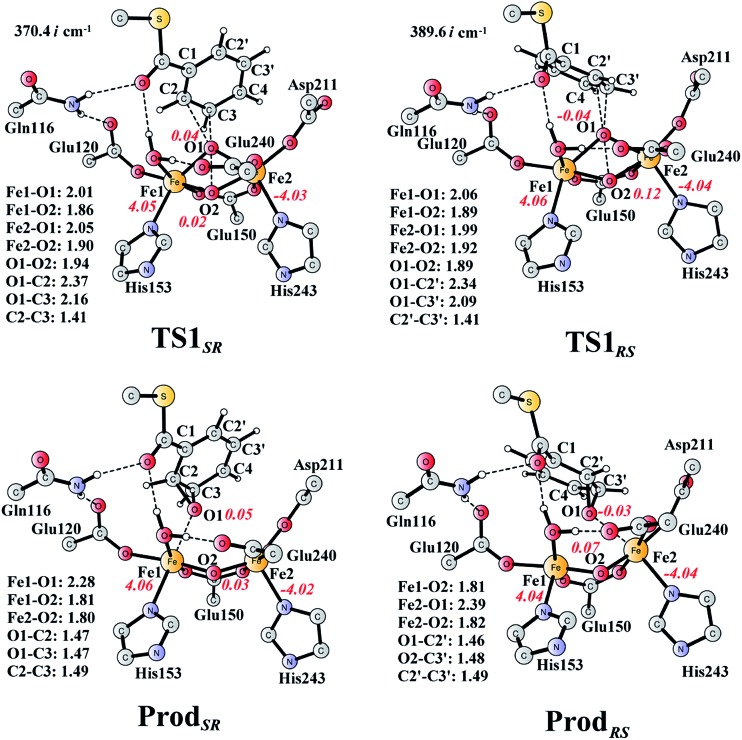
Optimized structures of transition states and products. Distances are given in Ångstrom, and spin densities are shown in red italic. The imaginary frequencies for **TS1_*SR*_** and **TS1_*RS*_** are indicated. For full model, see Fig. 2.

**Fig. 5:**
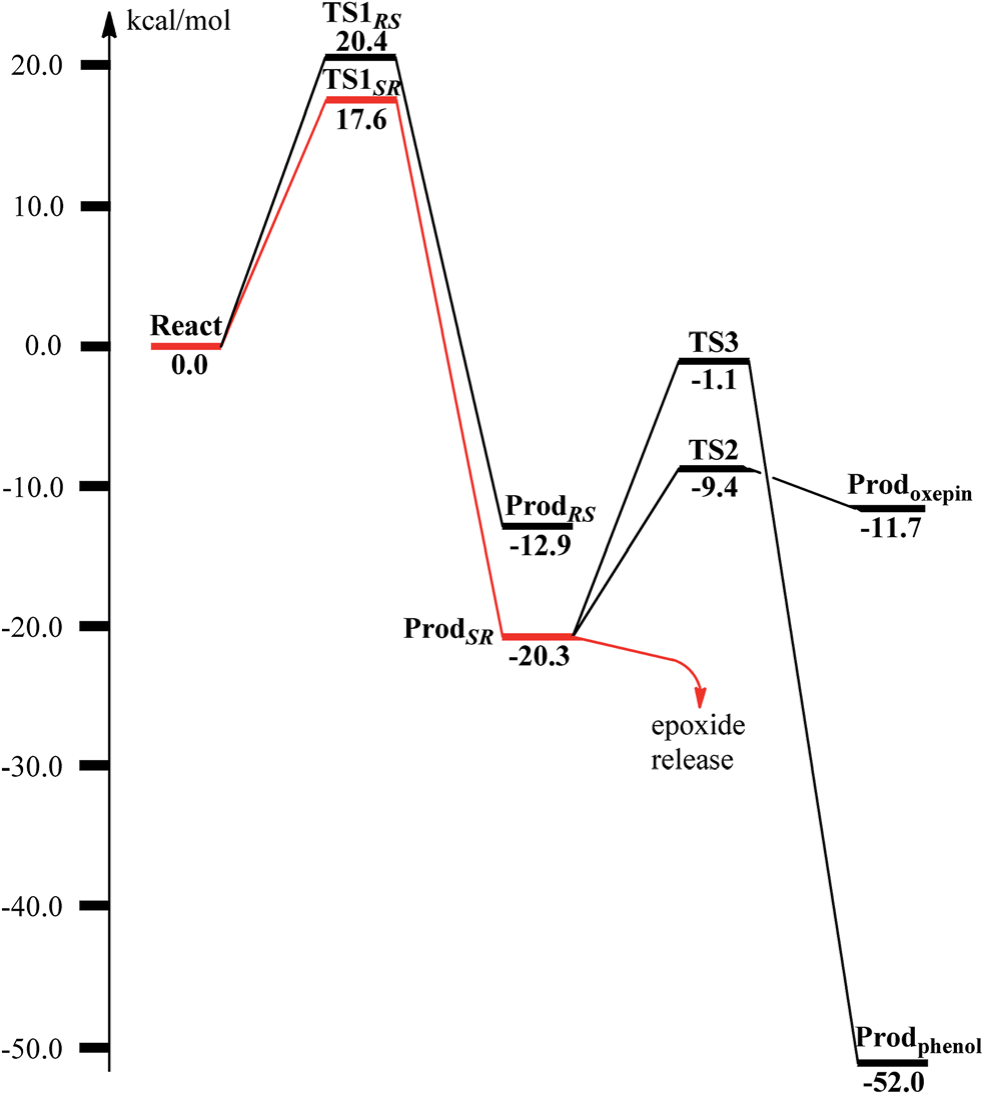
Calculated potential energy profile for the BoxB catalyzed benzoyl-CoA epoxidation.

From **React_A_**, the reaction was proposed to be initiated *via* O–O bond cleavage, followed by attack on the benzene ring.^17^ However, the calculations showed that the elongation of the O–O bond leads to a simultaneous attack on the aromatic carbon. No stable bis-μ-oxo-bridged diferryl intermediate (compound Q) can be located, which is different from the cases in MMO and RNR.^45^ In principle, there are six possibilities for the attack on the aromatic ring, but only two of them could be located, namely, attack on C2–C3 (**TS1_*SR*_**, *S* on C2 and *R* on C3, Fig. 4) and C2′–C3′ (**TS1_*RS*_**, *R* on C2′ and *S* on C3′, Fig. 4). The geometric constraints dictate that O1 (Fig. 3) is not able to reach C1, which rules out the attack on C1–C2 and C1–C2′. In addition, any attempt to locate transition states for the attack on C3–C4 or C3′–C4 leads to convergence to either **TS1_*SR*_** or **TS1_*RS*_**. The natures of **TS1_*SR*_** and **TS1_*RS*_** were confirmed to have imaginary frequencies of 370.4i and 386.9i cm^–1^, respectively, which mainly correspond to the cleavage of O1–O2 and the C–O bond formation. The barriers for **TS1_*SR*_** and **TS1_*RS*_** are 17.6 and 20.4 kcal mol^–1^, respectively (Fig. 5). The energy difference is 2.8 kcal mol^–1^, which can be translated into a diastereomeric excess of about 99 : 1 at room temperature using classical transition state theory. We also performed single-point calculations using the B3LYP-D3 and M06-D3^69^ functionals (including solvation and ZPE corrections from B3LYP). At the B3LYP-D3 level, the barriers for **TS1_*SR*_** and **TS1_*RS*_** are 20.1 and 22.4 kcal mol^–1^, respectively, while they are 22.9 and 25.6 kcal mol^–1^ at the M06-D3 level. All three functionals used predict that the attack on C2–C3 is preferred, which is consistent with the indication from the crystal structure.^17^ It should be pointed out that a definite and quantitative conclusion can only be made from measuring the product ratio ((2*S*,3*R*) *vs.* (2*R*,3*S*)). The formation of the product (**Prod_*SR*_**) is exergonic by 20.3 kcal mol^–1^, while it is less exergonic for the formation of **Prod_*RS*_**. These results suggest that the formation of (2*S*,3*R*)-epoxy-benzoyl-CoA is both kinetically and thermodynamically more favored.

At **TS1_*SR*_**, the scissile O1–O2 bond is 1.94 Å, which is significantly elongated from 1.45 Å in **React_A_**, and the two nascent C2–O1 and C3–O1 bonds are 2.37 and 2.16 Å, respectively. In addition, the Fe–Fe distance decreases from 3.68 Å in **React_A_** to 3.33 Å in **TS1_*SR*_**. O–O bond cleavage may lead to the formation of two ferryl (Fe^IV^) ions, however, the change of spin densities on both irons are very small, from **React_A_** to **TS1_*SR*_**, and to **Prod_*SR*_** (Figs. 3 and 4). This would suggest a heterolytic cleavage of the peroxide (O_2_^2–^) and a concerted oxygen atom transfer from the peroxide to the C–C moiety of the aromatic ring, while the metal keeps its oxidation at +3 during the epoxidation reaction.

**Fig. 6:**
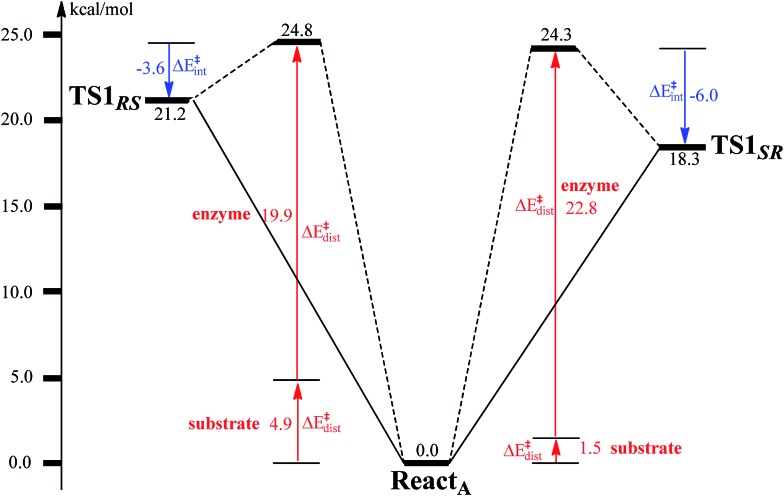
Distortion/interaction analysis for benzoyl-CoA epoxidation.

To unveil the source of regio- and stereo-selectivity of the enzyme, a distortion/interaction analysis was conducted, which has successfully been applied in the explanation of various reactivity trends.^70–73^ In the analysis, the full model is divided into two parts, the benzoyl-CoA substrate, and the enzyme (rest of the model). Single-point calculations were performed for each part in **React_A_**, **TS1_*SR*_**, and **TS1_*RS*_**. The total activation energy (without ZPE correction) is decomposed into the sum of the distortion energies of each part (Δ*E*‡dist) and the interaction energy (Δ*E*‡int) between the two distorted parts. As shown in Fig. 6, the distortion energy for the attack on C2–C3 is slightly lower (24.3 *vs.* 24.8 kcal mol^–1^) than that for the attack on C2′–C3′, while the interaction energy further favors the attack on C2–C3 by 2.4 kcal mol^–1^. The regio- and stereo-selectivity of epoxidation is thus mainly interaction-controlled. Inspection of the optimized structures of **TS1_*SR*_**, and **TS1_*RS*_** (Fig. S1†) indicates that the selectivity mainly originates from the steric repulsion between the aromatic ring and the Thr210-Asp211 peptide. The enzyme thus arranges its active-site residues to favor the attack on C2–C3 to generate the (2*S*,3*R*)-epoxy-benzoyl-CoA.

### 3.2 Interconversion of epoxide and oxepin products

**Fig. 7:**
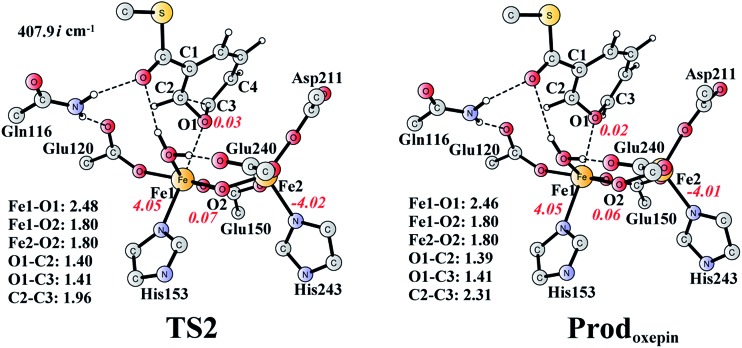
Optimized structures of **TS2** and the oxepin product (**Prod_oxepin_**). Distances are given in Ångstrom, and spin densities are shown in red italic. The imaginary frequency for **TS2** is indicated. For full model, see Fig. 2.

The (2*S*,3*R*)-epoxy-benzoyl-CoA can be converted into its oxepin isomer *via* direct epoxide C–C bond cleavage. Previous experimental and theoretical studies suggest that these two isomers are in fast equilibrium.^74–80^ Here, we considered this reaction inside the active site. The optimized structures of the transition state **TS2** and the oxepin product **Prod_oxepin_** are shown in Fig. 7. At **TS2**, the critical C2–C3 bond distance is 1.96 Å. The barrier was calculated to be 10.9 kcal mol^–1^, and the reaction is endergonic by 8.6 kcal mol^–1^, suggesting that this reaction cannot happen inside the active site.

**Fig. 8:**
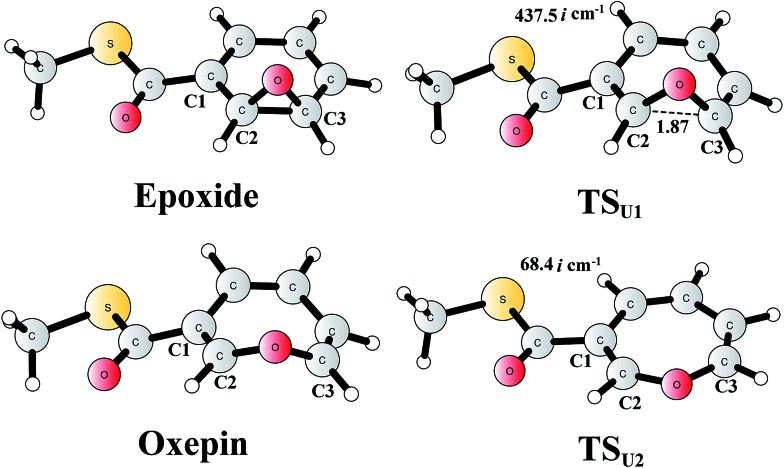
Optimized structures of the epoxide, oxepin, and transition states. Distances are given in Ångstrom. The imaginary frequencies for the transition states are indicated.

The reaction in the gas phase and water solution has also been calculated. Here, the substrate is further truncated with only one methyl group attached to the sulfur atom (Fig. 8). In the gas phase, the oxepin product is 1.0 kcal mol^–1^ (Gibbs free energy) lower in energy than the epoxide product, and the free energy barrier for the conversion (**TS_U1_**) is only 5.1 kcal mol^–1^ relative to the epoxide. In water solution, the oxepin becomes less stable, 2.1 kcal mol^–1^ higher than the epoxide, and the barrier increases slightly to 6.5 kcal mol^–1^. Further single-point calculations at the CCSD(T)/6-311+G(2d,2p) level^81^ (with solvation and free energy corrections from B3LYP) gave very similar results, with a barrier of 8.5 kcal mol^–1^ and an endergonic reaction of 2.8 kcal mol^–1^. The reason for the difference between the reaction in the enzyme and the reaction in water solution is that the size of the oxepin is larger than that of the epoxide, and steric repulsion can be observed between the enzyme active site and the oxepin product. In solution, the (2*S*,3*R*)-epoxy-benzoyl-CoA product and its enantiomer may interconvert *via* the inversion of the seven-membered ring of their oxepin form. The transition state (**TS_U2_**) has a planar structure on its seven-membered ring, and the barrier was calculated to be only 6.4 kcal mol^–1^ relative to the epoxide (8.0 kcal mol^–1^ at the CCSD(T) level). These results suggest that the epoxide is the major product, and a racemic mixture is obtained, even though the (2*S*,3*R*)-epoxy-benzoyl-CoA product is generated by the enzyme.

### 3.3 Conversion of epoxide to phenol

**Fig. 9:**
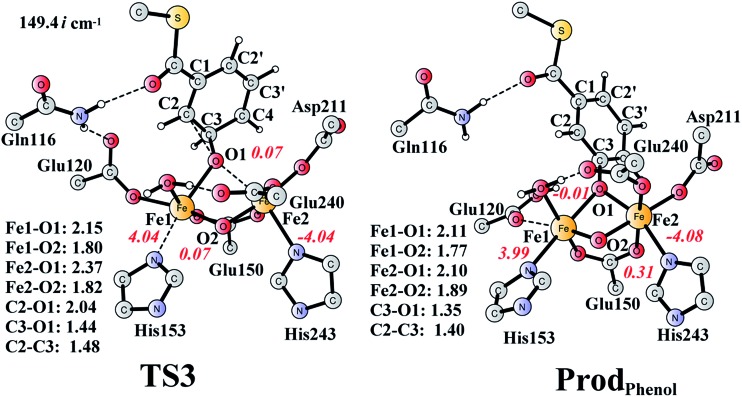
Optimized structures of **TS3** and the phenol product (**Prod_Phenol_**). Distances are given in Ångstrom, and spin densities are shown in red italic. The imaginary frequency for **TS3** is indicated. For full model, see Fig. 2.

It is known that phenol is more stable than epoxide, and the opening of the epoxide ring followed by proton transfer can generate phenol. If this reaction is faster than the product release, phenol will be seen as the major product. This undesired pathway is thus considered here. We found that the opening of the ring is coupled with proton transfer from C3 to Glu120, leading to a phenolate product bound to the di-iron center. The structures of the transition state **TS3** and the resulting product **Prod_Phenol_** are shown in Fig. 9. The barrier was calculated to be 19.2 kcal mol^–1^ relative to **Prod_*SR*_**, which is even slightly higher than that for epoxide formation (17.6 kcal mol^–1^). The reaction is exergonic by as much as 31.7 kcal mol^–1^. At **TS3**, both Fe keep their oxidation state at +3, and the cleavage of C2–O1 is a heterolytic fission. A carbocation is generated on the ring, and the proton at C3 can be easily released without the location of a stable intermediate. Considering the relatively high barrier for the ring opening to form phenol, the epoxide product release from **Prod_*SR*_** appears to be much more favored.

**Fig. 10:**
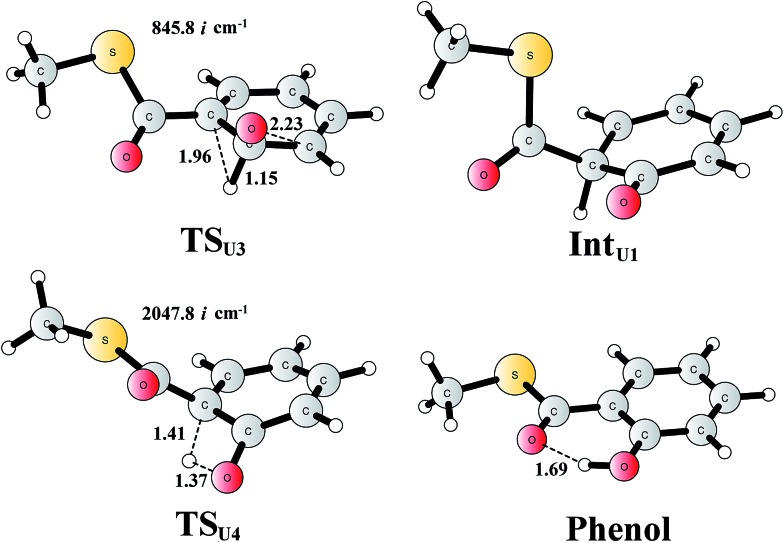
Optimized structures of the transition states, intermediates, and phenol product. Distances are given in Ångstrom. The imaginary frequencies for the transition states are indicated.

For comparison, we also considered epoxide phenol tautomerization in water solution. The calculations showed that the tautomerization takes place in two steps. First, the C–O bond cleavage and the [1,2]-hydride transfer take place concertedly (**TS_U3_**, Fig. 10), associated with a barrier of 29.0 kcal mol^–1^ (35.0 kcal mol^–1^ at the CCSD(T) level). This leads to the formation of a ketone intermediate, which lies at –23.7 kcal mol^–1^ (–24.2 kcal mol^–1^ at the CCSD(T) level) relative to **Epoxide**. Further [1,3]-proton transfer (**TS_U4_**) results in the formation of the phenol product, the barrier is calculated to be 46.9 kcal mol^–1^ (52.3 kcal mol^–1^ at the CCSD(T) level) relative to **Int_U1_**,^82^ and the whole reaction is exergonic by 48.0 kcal mol^–1^ (45.0 kcal mol^–1^ at the CCSD(T) level). The high barrier suggests that the epoxide is quite stable in water solution, which is also in agreement with previous experimental and theoretical studies.^79,83^ Therefore, the epoxide phenol tautomerization will not happen either in enzyme or in solution.

### 3.4 Deoxygenation of the (2*S*,3*R*)-epoxy-benzoyl-CoA

**Fig. 11:**
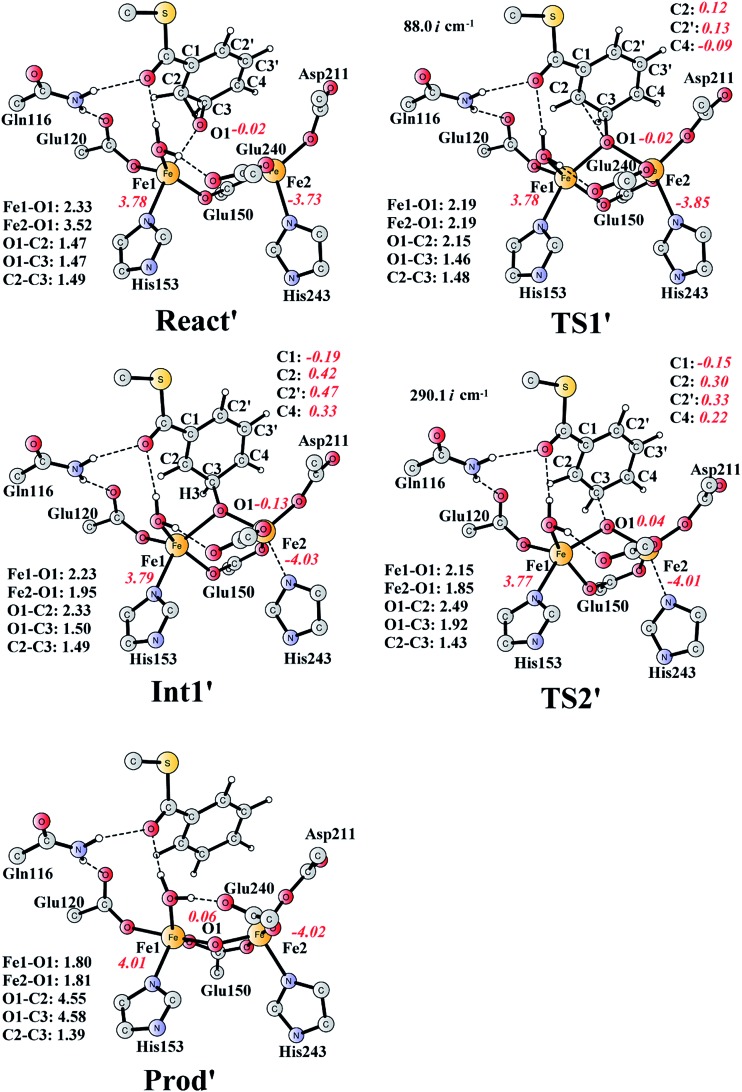
Optimized structures of various stationary points along the deoxygenation pathway. Distances are given in Ångstrom, and spin densities are shown in red italic. The imaginary frequencies for **TS1′** and **TS2′** are indicated. For full model, see Fig. 2. **React′** is derived from **Prod_*SR*_** by removing the O2 atom.

The deoxygenation of (2*S*,3*R*)-epoxy-benzoyl-CoA by the Fe_2_(ii,ii) form of the enzyme has been proposed as discussed in the Introduction part. To investigate this interesting reaction, the bridging oxygen atom is removed from **Prod_*SR*_**, and this new complex is labelled as **React′** (Fig. 11). **React′** is composed of two ferrous ions, with spin densities on Fe1 and Fe2 of 3.78 and –3.73 respectively.

**Fig. 12:**
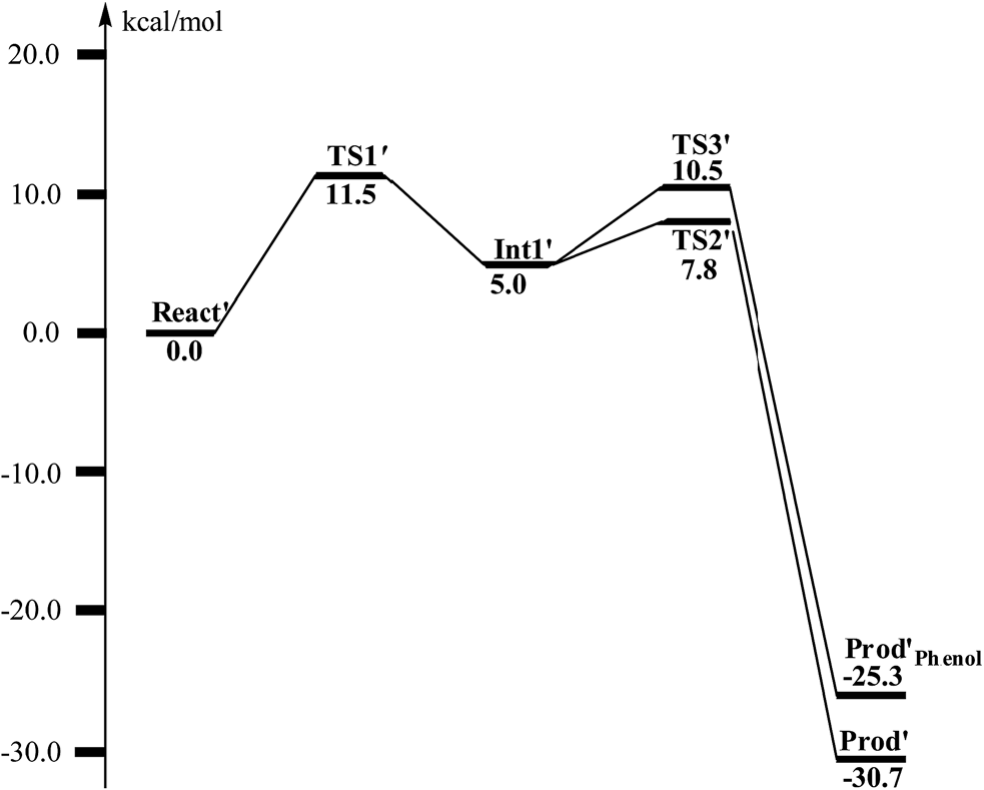
Calculated potential energy profile for the BoxB catalyzed deoxygenation of (2*S*,3*R*)-epoxy-benzoyl-CoA.

From **React′**, the deoxygenation reaction was calculated to take place *via* a stepwise mechanism, which is different from the concerted mechanism for the epoxidation reaction as discussed above. The reaction starts from the cleavage of the epoxide C2–O1 bond *via* **TS1′** (Fig. 11), and the barrier for this step was calculated to be only 11.5 kcal mol^–1^ (Fig. 12), which is much lower than that for epoxide ring cleavage from **Prod_*SR*_** *via* **TS3** (barrier of 19.2 kcal mol^–1^). The reason is that two ferrous ions are present in **React′**, and during the C2–O1 heterolytic cleavage, one electron is transferred from Fe1 to the six-membered ring, generating a ferric ion and a substrate radical. This is evidenced by the decrease of spin density on Fe1 and the increase of spin densities on the substrate (C2, C2′ and C4, see Fig. 11). Due to this electron transfer, a stable intermediate (**Int1′**) can be located, and its energy is 5.0 kcal mol^–1^ relative to **React′**. In contrast, **Prod_*SR*_** has a diferric center, and electron transfer from a ferric center to the substrate during the epoxide ring cleavage is energetically very unfavorable. At **TS1′**, the critical C2–O1 distance is 2.15 Å, which is further increased to 2.33 Å at **Int1′**.

From **Int1′**, the cleavage of the C3–O1 bond leads to the desired deoxygenation reaction, in which a benzene ring is formed. The optimized structures of the transition state **TS2′** and the resulting product **Prod′** are shown in Fig. 11. The energy of **TS2′** is only 2.8 kcal mol^–1^ higher than that of **Int1′**, *ca.* 7.8 kcal mol^–1^ higher than that of **React′**. In this step, one electron is transferred from Fe2 to the substrate, which facilitates the C3–O1 bond cleavage. Fe2 is thus oxidized from +2 to +3, and a diferric center is formed in **Prod′**. At **TS2′**, the distance between C3 and O1 is 1.92 Å. The overall reaction was calculated to be exergonic by 30.7 kcal mol^–1^.

**Fig. 13:**
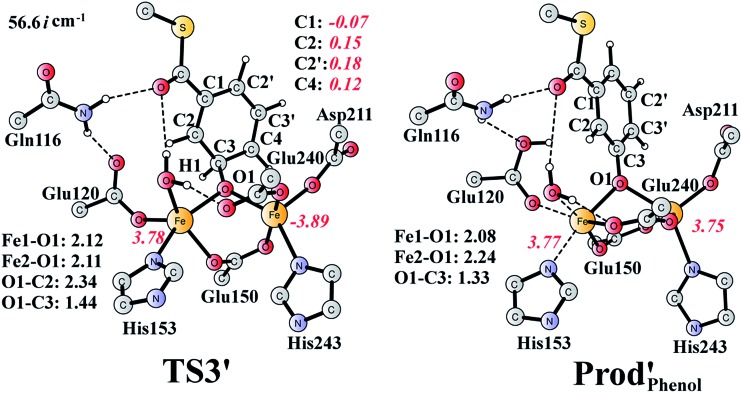
Optimized structures of **TS3′** and the phenol product (**Prod′_Phenol_**). Distances are given in Ångstrom, and spin densities are shown in red italic. The imaginary frequency for **TS3′** is indicated.

It should be pointed out that there is a competing pathway, in which a proton is released from C3 to Glu120 and a phenolate product is generated. This is very similar to the epoxide phenol isomerization as discussed in Section 3.3. In order to obtain the deoxygenation product, this pathway must have a higher barrier compared to that for the C3–O1 bond cleavage at **TS2′**. The transition state for this pathway (**TS3′**) has been located and is shown in Fig. 13. The major structural change from **Int1′** to **TS3′** is the decrease of the H3–C2–C3–C4 dihedral angle from 124.2° to 115.3°, which is coupled to a partial electron transfer from the substrate ring to Fe2, evidenced by the decrease of spin density on the substrate (Fig. 13). The barrier was calculated to be 5.5 kcal mol^–1^ relative to **Int1′**, which is 2.7 kcal mol^–1^ higher than that for the deoxygenation reaction. In addition, this step is exergonic by 30.3 kcal mol^–1^, however, this is also less than that for the formation of **Prod′**. These results suggest that the deoxygenation reaction is both kinetically and thermodynamically favored compared with the epoxide–phenol isomerization. Further experimental studies are needed to verify our proposal.

## 4. Conclusion

In this work, we have designed a quite large model of the active site of the di-Fe-dependent benzoyl-CoA epoxidase and used density functional theory calculations to investigate its reaction mechanism and selectivity. On the basis of our calculations, we have proposed a concerted oxygen atom transfer mechanism for the aromatic ring epoxidation reaction, and a stepwise pathway for the suggested epoxide deoxygenation reaction.

The substrate epoxidation reaction starts with the binding of an O_2_ molecule to the di-Fe(ii) center in a side-on symmetric fashion to generate a diferric-peroxide. O–O bond cleavage then takes place, concomitant with the formation of epoxide. The attack can proceed at either C2–C3 or C2′–C3′ positions, leading to either (2*S*,3*R*)-epoxy or (2*R*,3*S*)-epoxy products. The attack on C2–C3 is preferred with a barrier of 17.6 kcal mol^–1^, with the barrier for the attack on C2′–C3′ being 2.8 kcal mol^–1^ higher. Further conversion of epoxide to oxepin is thermodynamically unfavorable in the enzyme due to larger steric repulsion between the enzyme active site and the oxepin. However, this conversion is both kinetically and thermodynamically facile in water solution. In addition, the (2*S*,3*R*)-epoxide and the (2*R*,3*S*)-epoxide can interconvert *via* the inversion of the seven-membered ring of their oxepin forms. Thus, even though (2*S*,3*R*)-epoxide is generated by the enzyme, racemic product would be obtained due to very fast racemization in solution.

The isomerization of epoxide to phenol is found to have a barrier of 19.2 kcal mol^–1^, which should be much higher than that for the product release from the enzyme active site. The results therefore indicate that the epoxide is the sole product of this enzyme.

For the suggested unprecedented deoxygenation reaction, a stepwise pathway is suggested, which is different from the concerted pathway for the epoxidation reaction. The reaction starts with a C–O bond cleavage to generate a substrate radical intermediate, in which one electron is transferred from the diferrous center to the substrate ring. This is followed by the second C–O bond cleavage, coupled with a second electron transfer from the metal center to the substrate. The first step is calculated to be rate-limiting, with a barrier of only 11.5 kcal mol^–1^. The conversion of the substrate radical intermediate to phenol is found to have a higher barrier, suggesting that the deoxygenation reaction is plausible for BoxB. Further experimental studies are needed to verify our results.
