# Supplementary material for: Mechanism and selectivity of the dinuclear iron benzoyl-coenzyme A epoxidase BoxB
Source: Chem Sci. 2015 Mar 2;6(5):2754–64. doi: 10.1039/c5sc00313j (PMC5489048; doi:10.1039/c5sc00313j)
Supplement: Supplementary file 1 [file SC-006-C5SC00313J-s001.pdf]

Electronic Supplementary Material (ESI) for Chemical Science.
This journal is © The Royal Society of Chemistry 2015

Supporting information to:

Mechanism and Selectivity of the Dinuclear Iron Benzoyl-Coenzyme A Epoxidase BoxB

Rong-Zhen Liao[1], and Per E. M. Siegbahn[2]**

[1]Key Laboratory for Large-Format Battery Materials and System, Ministry of Education, School of Chemistry and Chemical Engineering, Huazhong University of Science and Technology, Wuhan 430074, China.

[2]Department of Organic Chemistry, Arrhenius Laboratory, Stockholm University, SE-10691 Stockholm, Sweden.

Corresponding author e-mail:

rongzhen@hust.edu.cn; ps@organ.su.se

Cartesian coordinates for all optimized structures.

React$_A$

E(B3LYP*/LB)= -5205.901452 Number of imaginary frequencies: 6

Center Number	Atomic Number	Atomic Type	Coordinates (Angstroms) X	Y	Z
1	26	0	-0.497484	-3.085848	0.718276
2	26	0	-3.622222	-1.613412	-0.537909
3	6	0	1.957996	0.125024	-2.074009
4	16	0	2.818057	1.270605	-3.133860
5	6	0	0.977649	0.747395	-1.144882
6	6	0	4.059808	0.180066	-3.932276
7	6	0	0.199399	1.857875	-1.504176
8	6	0	5.332090	0.072988	-3.073327
9	6	0	-0.745964	2.352240	-0.601934
10	7	0	6.358415	-0.753414	-3.679081
11	8	0	2.186683	-1.087245	-2.079172
12	6	0	-0.899399	1.773284	0.660387
13	6	0	7.436654	-0.195096	-4.309774
14	8	0	7.543402	1.014491	-4.497777
15	6	0	-0.080831	0.704567	1.040404
16	6	0	8.505215	-1.185893	-4.772089
17	6	0	0.841469	0.181121	0.135721
18	6	0	9.919279	-0.642252	-4.529170
19	6	0	7.692364	-1.948557	-0.043819
20	6	0	7.643195	-3.476901	-0.144820
21	6	0	6.218905	-4.026601	-0.314490
22	6	0	5.563396	-3.525519	-1.597623
23	8	0	6.182731	-3.492674	-2.668652
24	7	0	4.272110	-3.141075	-1.485693
25	6	0	4.537881	1.507910	3.187235
26	6	0	3.356118	0.963731	4.005976
27	8	0	2.426112	1.680820	4.394974
28	6	0	4.402667	1.097451	1.695980
29	8	0	4.769351	-0.294682	1.646252
30	6	0	5.344864	1.905506	0.809632
31	7	0	3.389295	-0.384058	4.222616
32	6	0	2.222960	-1.167336	4.675687
33	6	0	2.497586	-2.670434	4.569200
34	6	0	3.086510	-3.139620	3.217879
35	6	0	2.308065	-2.788536	1.944618
36	8	0	1.056009	-3.095028	1.938055
37	8	0	2.897364	-2.270814	0.976287
38	6	0	-0.819722	3.240586	5.692792
39	6	0	-0.934670	2.244001	4.534797
40	8	0	-0.338184	0.979637	4.811585
41	6	0	-1.893766	-4.045560	4.693279
42	6	0	-2.112900	-2.538900	4.921955
43	6	0	-3.073616	-1.847432	3.945296
44	6	0	-2.657169	-1.885841	2.481820
45	8	0	-1.489101	-2.346699	2.248766

46	8	0	-3.481655	-1.475070	1.617291
47	6	0	1.443479	-7.312037	3.368597
48	6	0	1.022698	-6.089523	2.534261
49	6	0	-0.019648	-6.327747	1.480177
50	7	0	-0.613066	-5.295689	0.747236
51	6	0	-0.465921	-7.520865	0.973453
52	6	0	-1.367882	-5.866169	-0.184244
53	7	0	-1.312957	-7.208542	-0.075229
54	6	0	3.515277	7.832481	3.753327
55	6	0	4.161742	6.448588	3.562979
56	6	0	3.748866	5.808602	2.253454
57	6	0	2.758016	4.817299	2.218214
58	6	0	4.304739	6.238420	1.038966
59	6	0	2.319009	4.286435	1.002326
60	6	0	3.874108	5.706125	-0.176818
61	6	0	2.872869	4.731678	-0.199608
62	6	0	-5.705837	7.387327	-1.281616
63	6	0	-5.347369	7.703791	0.187339
64	6	0	-4.149271	6.981961	0.767003
65	6	0	-2.917828	7.633588	0.920270
66	6	0	-4.257444	5.656416	1.212729
67	6	0	-1.830951	6.984775	1.510220
68	6	0	-3.175561	4.999631	1.802812
69	6	0	-1.956603	5.666418	1.953560
70	6	0	-1.967628	5.621154	-3.885586
71	6	0	-1.848216	4.091514	-3.903955
72	8	0	-0.871887	3.526164	-4.395694
73	6	0	-1.882696	6.294610	-2.501418
74	8	0	-2.321170	7.652145	-2.594054
75	6	0	-0.478494	6.205208	-1.907104
76	6	0	-8.562956	-1.952007	-2.122858
77	6	0	-7.109957	-1.781551	-1.649236
78	6	0	-6.681283	-2.755859	-0.594103
79	7	0	-5.387443	-2.784876	-0.079944
80	6	0	-7.416001	-3.721938	0.044307
81	6	0	-5.352198	-3.737711	0.838149
82	7	0	-6.558534	-4.332825	0.942863
83	7	0	-2.904673	3.371370	-3.406763
84	6	0	-2.860757	1.909073	-3.451254
85	6	0	-4.136340	1.232074	-2.928831
86	6	0	-4.234464	1.303783	-1.406570
87	8	0	-4.341509	2.386760	-0.835409
88	8	0	-4.197648	0.163731	-0.743355
89	6	0	-5.065818	-5.199839	-3.650567
90	6	0	-3.918625	-4.762165	-4.577343
91	6	0	-3.632967	-3.246837	-4.549451
92	6	0	-2.950795	-2.834344	-3.240710
93	8	0	-1.788797	-3.196888	-3.029470
94	8	0	-3.705573	-2.125983	-2.437656
95	8	0	-4.007356	1.673837	2.011880
96	8	0	0.462535	-3.146444	-1.174516
97	8	0	-1.698497	-1.896744	-0.458074
98	8	0	-2.241505	-3.226899	-0.259331
99	1	0	8.085033	-3.928784	0.751877

100	1	0	8.242276	-3.809076	-0.999127
101	1	0	6.248218	-5.122565	-0.372789
102	1	0	5.597540	-3.765446	0.550170
103	1	0	3.829967	-2.982317	-0.586372
104	1	0	3.811832	-2.727703	-2.284755
105	1	0	7.364127	-1.488762	-0.981417
106	1	0	3.369535	1.228883	1.350837
107	1	0	5.282635	1.555994	-0.224839
108	1	0	6.379521	1.780879	1.146966
109	1	0	5.089810	2.968411	0.829739
110	1	0	4.085793	-0.836106	1.214418
111	1	0	4.621202	2.584160	3.329680
112	1	0	1.553738	-3.192341	4.754693
113	1	0	3.197052	-2.981316	5.354557
114	1	0	3.157480	-4.233774	3.248700
115	1	0	4.108379	-2.777705	3.078336
116	1	0	1.356140	-0.913772	4.051439
117	1	0	-1.984712	2.057135	4.284274
118	1	0	-0.470432	2.688519	3.640984
119	1	0	0.625961	1.137419	4.808486
120	1	0	-1.008867	4.256400	5.322946
121	1	0	-2.509776	-2.385663	5.932894
122	1	0	-1.154007	-2.012840	4.888370
123	1	0	-4.085953	-2.268997	4.001746
124	1	0	-3.178701	-0.788219	4.212219
125	1	0	-1.384763	-4.238031	3.749010
126	1	0	0.694802	-5.278874	3.189560
127	1	0	1.905479	-5.699453	2.014004
128	1	0	-0.251057	-8.539608	1.251190
129	1	0	-1.781001	-7.864953	-0.681608
130	1	0	-1.923777	-5.331933	-0.940631
131	1	0	1.633327	-8.188515	2.739554
132	1	0	3.873197	5.794753	4.393821
133	1	0	5.253784	6.548875	3.606244
134	1	0	5.082887	6.998772	1.047668
135	1	0	2.338291	4.440621	3.148002
136	1	0	4.321780	6.048445	-1.105918
137	1	0	1.546127	3.523930	0.996050
138	1	0	2.538063	4.312596	-1.143863
139	1	0	2.427645	7.764165	3.644392
140	1	0	-6.223492	7.474961	0.808384
141	1	0	-5.187314	8.785014	0.277012
142	1	0	-2.813650	8.660160	0.578455
143	1	0	-5.206305	5.134200	1.112292
144	1	0	-0.887915	7.511662	1.631514
145	1	0	-3.295991	3.979825	2.156661
146	1	0	-1.110647	5.164616	2.415245
147	1	0	-4.919418	7.712462	-1.968130
148	1	0	-2.598706	5.831135	-1.813594
149	1	0	0.241997	6.724308	-2.552692
150	1	0	-0.143924	5.167705	-1.818493
151	1	0	-0.456287	6.660833	-0.915822
152	1	0	-1.660787	8.136129	-3.109150
153	1	0	-1.151032	5.984071	-4.517702

154	1	0	-6.418431	-1.869784	-2.495347
155	1	0	-6.965768	-0.767094	-1.256503
156	1	0	-8.447711	-4.016763	-0.054355
157	1	0	-6.794905	-5.076162	1.582401
158	1	0	-4.489515	-4.005662	1.426746
159	1	0	-9.272840	-1.840982	-1.296730
160	1	0	-4.130343	0.186491	-3.242622
161	1	0	-5.024410	1.717959	-3.351180
162	1	0	-2.006998	1.542659	-2.871044
163	1	0	3.592473	-0.792171	-4.103341
164	1	0	4.283610	0.641918	-4.896854
165	1	0	0.281443	2.296975	-2.494915
166	1	0	5.071666	-0.340498	-2.094075
167	1	0	5.753650	1.070918	-2.930953
168	1	0	-1.388502	3.176622	-0.890548
169	1	0	-1.678993	2.122114	1.329965
170	1	0	-0.170962	0.288588	2.039769
171	1	0	8.362520	-2.157183	-4.286085
172	1	0	8.353722	-1.343993	-5.848198
173	1	0	1.484331	-0.648707	0.411001
174	1	0	10.672446	-1.301651	-4.971236
175	1	0	10.129175	-0.557312	-3.457962
176	1	0	10.015734	0.353167	-4.968460
177	1	0	-5.004252	-4.691612	-2.681633
178	1	0	-4.144537	-5.066307	-5.606048
179	1	0	-2.991573	-5.277412	-4.300040
180	1	0	-4.555833	-2.675506	-4.688772
181	1	0	-2.945053	-2.996709	-5.364041
182	1	0	-4.190439	1.993237	1.109325
183	1	0	-3.899912	0.720180	1.885271
184	1	0	8.709633	-1.602536	0.140469
185	1	0	7.047686	-1.566317	0.755374
186	1	0	5.452280	0.972050	3.442169
187	1	0	1.950753	-0.869363	5.687875
188	1	0	2.384034	-7.077032	3.866753
189	1	0	0.695232	-7.582483	4.118867
190	1	0	-1.288825	-4.447536	5.506131
191	1	0	-2.845929	-4.592806	4.694298
192	1	0	-5.013101	-6.271647	-3.458781
193	1	0	-6.045473	-4.969430	-4.081568
194	1	0	-8.725714	-2.927550	-2.592107
195	1	0	-8.780981	-1.176584	-2.857130
196	1	0	-2.654761	1.599689	-4.475999
197	1	0	-5.881779	6.316273	-1.428144
198	1	0	-6.633117	7.921280	-1.490430
199	1	0	-2.905476	5.895944	-4.368298
200	1	0	3.875588	8.542712	3.001951
201	1	0	3.720263	8.246674	4.740545
202	1	0	0.195188	3.226131	6.104046
203	1	0	-1.530515	3.072744	6.502024
204	1	0	4.077815	-0.857378	3.649308
205	1	0	6.333626	-1.756875	-3.498157
206	1	0	-3.585440	3.803696	-2.795764
207	1	0	-0.280152	-3.069502	-1.823003

208	1	0	1.085275	-2.410016	-1.381544

React$_B$
E(B3LYP*/LB)= -5205.892693 Number of imaginary frequencies: 8

Center Number	Atomic Number	Atomic Type	Coordinates (Angstroms) X	Y	Z
1	26	0	-0.982374	-3.195115	0.652860
2	26	0	-4.029718	-0.782806	-0.434638
3	6	0	2.009442	-0.058980	-2.191613
4	16	0	2.983552	0.861079	-3.376591
5	6	0	1.253961	0.791933	-1.235080
6	6	0	4.042123	-0.461065	-4.079398
7	6	0	0.623420	1.983458	-1.628408
8	6	0	5.280065	-0.681970	-3.192517
9	6	0	-0.132647	2.693283	-0.695279
10	7	0	6.186682	-1.684262	-3.715375
11	8	0	2.002938	-1.290045	-2.143240
12	6	0	-0.220147	2.260325	0.631176
13	6	0	7.351709	-1.326670	-4.334699
14	8	0	7.645074	-0.158174	-4.578610
15	6	0	0.461058	1.111447	1.035046
16	6	0	8.277899	-2.484687	-4.703671
17	6	0	1.171375	0.362051	0.097666
18	6	0	9.740593	-2.144982	-4.389179
19	6	0	7.258424	-3.188587	0.030522
20	6	0	6.967168	-4.680611	-0.156851
21	6	0	5.470474	-4.978318	-0.343543
22	6	0	4.903988	-4.274521	-1.573078
23	8	0	5.497356	-4.308910	-2.659272
24	7	0	3.729458	-3.632305	-1.396933
25	6	0	4.613246	0.642371	3.296406
26	6	0	3.324852	0.270743	4.039429
27	8	0	2.470990	1.110821	4.351186
28	6	0	4.478366	0.320119	1.782374
29	8	0	4.549400	-1.114072	1.672649
30	6	0	5.620932	0.937368	0.982658
31	7	0	3.164287	-1.062451	4.273541
32	6	0	1.888730	-1.676279	4.686395
33	6	0	1.972497	-3.199842	4.573026
34	6	0	2.448034	-3.708247	3.194487
35	6	0	1.673190	-3.177823	1.987088
36	8	0	0.386170	-3.281496	2.060479
37	8	0	2.276236	-2.711985	1.002984
38	6	0	-0.459849	3.124390	5.763453
39	6	0	-0.734378	2.264890	4.527520
40	8	0	-0.373546	0.893711	4.694944
41	6	0	-2.620539	-3.886803	4.574867
42	6	0	-1.998116	-2.491682	4.404925
43	6	0	-2.817060	-1.443916	3.646380
44	6	0	-3.119426	-1.722763	2.190279
45	8	0	-2.800819	-2.822518	1.639298

46	8	0	-3.744119	-0.805375	1.553219
47	6	0	0.199608	-7.598845	3.221787
48	6	0	0.009945	-6.366233	2.324695
49	6	0	-1.127486	-6.429336	1.345426
50	7	0	-1.519002	-5.325060	0.587963
51	6	0	-1.883744	-7.506562	0.959849
52	6	0	-2.470872	-5.737967	-0.234671
53	7	0	-2.727173	-7.051323	-0.037867
54	6	0	4.562854	7.035267	3.998053
55	6	0	5.160829	5.644580	3.720702
56	6	0	4.816302	5.148903	2.331713
57	6	0	3.682569	4.354438	2.108950
58	6	0	5.591466	5.527591	1.225896
59	6	0	3.323312	3.965656	0.815883
60	6	0	5.241211	5.134619	-0.066648
61	6	0	4.100712	4.356109	-0.276585
62	6	0	-4.524242	8.120858	-1.181207
63	6	0	-4.089012	8.429987	0.268750
64	6	0	-2.997764	7.563064	0.859876
65	6	0	-1.697832	8.058078	1.031568
66	6	0	-3.274794	6.261621	1.303391
67	6	0	-0.707585	7.282202	1.637654
68	6	0	-2.290920	5.477972	1.909958
69	6	0	-1.001781	5.990712	2.079861
70	6	0	-1.054577	5.856652	-3.771283
71	6	0	-1.175780	4.330025	-3.864032
72	8	0	-0.328022	3.649527	-4.441271
73	6	0	-0.894504	6.490693	-2.375714
74	8	0	-1.217454	7.883112	-2.440415
75	6	0	0.508904	6.284035	-1.808099
76	6	0	-8.764202	-0.648289	-2.287127
77	6	0	-7.355197	-0.636399	-1.684948
78	6	0	-7.117915	-1.685929	-0.642503
79	7	0	-5.880027	-1.851297	-0.028787
80	6	0	-7.983560	-2.608609	-0.113330
81	6	0	-5.998711	-2.845892	0.838097
82	7	0	-7.257671	-3.333001	0.816626
83	7	0	-2.314962	3.764901	-3.354375
84	6	0	-2.514117	2.317588	-3.438739
85	6	0	-3.912021	1.887893	-2.971767
86	6	0	-4.092984	2.046658	-1.462788
87	8	0	-3.864251	3.115971	-0.904177
88	8	0	-4.528699	0.988957	-0.800236
89	6	0	-5.779912	-4.361799	-3.840051
90	6	0	-4.374340	-4.165113	-4.449579
91	6	0	-3.756648	-2.771266	-4.249386
92	6	0	-3.488306	-2.500285	-2.771931
93	8	0	-2.697018	-3.221503	-2.127193
94	8	0	-4.162043	-1.522869	-2.266354
95	8	0	-3.466305	2.350247	1.970680
96	8	0	-0.042216	-3.341825	-1.294401
97	8	0	-1.169868	-1.237549	0.130453
98	8	0	-2.074615	-0.708849	-0.634070
99	1	0	7.329865	-5.250453	0.707528

100	1	0	7.503455	-5.054560	-1.035239
101	1	0	5.321361	-6.056008	-0.488371
102	1	0	4.906628	-4.689733	0.551571
103	1	0	3.318581	-3.480056	-0.482442
104	1	0	3.335712	-3.090451	-2.154713
105	1	0	7.013463	-2.631860	-0.879864
106	1	0	3.520080	0.685208	1.396826
107	1	0	5.540731	0.650920	-0.069832
108	1	0	6.584192	0.574950	1.358072
109	1	0	5.602582	2.028514	1.045620
110	1	0	3.745004	-1.489245	1.275649
111	1	0	4.858584	1.687130	3.484436
112	1	0	0.981682	-3.608503	4.791001
113	1	0	2.656421	-3.594485	5.334106
114	1	0	2.352030	-4.800830	3.179735
115	1	0	3.506259	-3.493294	3.025342
116	1	0	1.079635	-1.297881	4.049832
117	1	0	-1.793302	2.287194	4.252757
118	1	0	-0.179813	2.692414	3.679366
119	1	0	0.603610	0.873194	4.704868
120	1	0	-0.435287	4.181933	5.470502
121	1	0	-1.803589	-2.061887	5.394231
122	1	0	-1.024117	-2.595970	3.919890
123	1	0	-3.783730	-1.261030	4.136451
124	1	0	-2.283734	-0.485140	3.684557
125	1	0	-2.553356	-4.483459	3.663227
126	1	0	-0.107583	-5.468527	2.936890
127	1	0	0.928088	-6.203969	1.743996
128	1	0	-1.889388	-8.532428	1.290171
129	1	0	-3.394005	-7.604652	-0.553564
130	1	0	-2.958389	-5.119700	-0.976752
131	1	0	0.229526	-8.524919	2.636860
132	1	0	4.790963	4.934325	4.469537
133	1	0	6.250562	5.688676	3.839143
134	1	0	6.479760	6.136310	1.381630
135	1	0	3.081998	4.023391	2.953637
136	1	0	5.859575	5.433350	-0.908838
137	1	0	2.438412	3.356188	0.664335
138	1	0	3.825874	4.044682	-1.280176
139	1	0	3.471758	7.011307	3.909286
140	1	0	-4.975682	8.356443	0.912787
141	1	0	-3.769780	9.478174	0.314389
142	1	0	-1.463561	9.063589	0.691580
143	1	0	-4.278597	5.858791	1.188090
144	1	0	0.291162	7.689769	1.772522
145	1	0	-2.540941	4.479180	2.255805
146	1	0	-0.231800	5.389649	2.556081
147	1	0	-3.709515	8.289469	-1.889868
148	1	0	-1.636235	6.079046	-1.682093
149	1	0	1.250643	6.775951	-2.451135
150	1	0	0.776863	5.225315	-1.757427
151	1	0	0.577511	6.707284	-0.804258
152	1	0	-0.522409	8.318216	-2.953440
153	1	0	-0.184921	6.111884	-4.385530

154	1	0	-6.604987	-0.768363	-2.472827
155	1	0	-7.145016	0.344290	-1.241499
156	1	0	-9.025828	-2.803809	-0.305239
157	1	0	-7.607533	-4.081729	1.394957
158	1	0	-5.203976	-3.236260	1.456033
159	1	0	-9.533551	-0.498763	-1.522160
160	1	0	-4.078692	0.844662	-3.248057
161	1	0	-4.677816	2.497543	-3.468321
162	1	0	-1.762924	1.792413	-2.836303
163	1	0	3.437488	-1.365114	-4.180771
164	1	0	4.335561	-0.116004	-5.073718
165	1	0	0.667812	2.323479	-2.659532
166	1	0	4.955355	-0.979048	-2.189885
167	1	0	5.836943	0.254604	-3.115317
168	1	0	-0.681574	3.575256	-1.002646
169	1	0	-0.851221	2.793856	1.332783
170	1	0	0.396623	0.782344	2.067862
171	1	0	7.963797	-3.404705	-4.198925
172	1	0	8.165911	-2.657982	-5.782136
173	1	0	1.639099	-0.574869	0.378015
174	1	0	10.412577	-2.924424	-4.761486
175	1	0	9.898431	-2.048280	-3.309970
176	1	0	10.008336	-1.192967	-4.852885
177	1	0	-5.880619	-3.858458	-2.872489
178	1	0	-4.407099	-4.391451	-5.521260
179	1	0	-3.683577	-4.889886	-4.003624
180	1	0	-4.405115	-1.987055	-4.648912
181	1	0	-2.797471	-2.724026	-4.778150
182	1	0	-3.662423	2.650255	1.066150
183	1	0	-3.587235	1.391420	1.935509
184	1	0	8.313173	-3.007100	0.237934
185	1	0	6.669023	-2.754456	0.845956
186	1	0	5.429759	-0.033272	3.551762
187	1	0	1.646893	-1.361689	5.701269
188	1	0	1.155986	-7.521926	3.739161
189	1	0	-0.597630	-7.690778	3.965572
190	1	0	-2.099293	-4.394579	5.386559
191	1	0	-3.677263	-3.823373	4.865877
192	1	0	-5.895530	-5.432648	-3.672995
193	1	0	-6.576935	-4.002752	-4.496684
194	1	0	-8.975571	-1.587380	-2.809133
195	1	0	-8.847815	0.167427	-3.005287
196	1	0	-2.339643	2.002312	-4.467497
197	1	0	-4.876616	7.089151	-1.284193
198	1	0	-5.354809	8.795294	-1.390791
199	1	0	-1.930156	6.282669	-4.261043
200	1	0	4.934633	7.769088	3.275162
201	1	0	4.810862	7.391950	4.997707
202	1	0	0.524362	2.882784	6.178998
203	1	0	-1.202588	3.050602	6.557898
204	1	0	3.823315	-1.642745	3.770709
205	1	0	5.993738	-2.662292	-3.498521
206	1	0	-2.868625	4.257321	-2.663644
207	1	0	-0.798854	-3.280558	-1.912468

208	1	0	0.568653	-2.602372	-1.493980

React$_C$

E(B3LYP*/LB)= -5205.876829 Number of imaginary frequencies: 9

Center Number	Atomic Number	Atomic Type	Coordinates (Angstroms) X	Y	Z
1	26	0	0.090435	-3.180533	0.679876
2	26	0	-3.675959	-1.756026	-0.485774
3	6	0	1.992398	0.295531	-2.102920
4	16	0	2.753594	1.521111	-3.140184
5	6	0	0.987094	0.814646	-1.140140
6	6	0	4.051998	0.527283	-3.974351
7	6	0	0.089795	1.838991	-1.478862
8	6	0	5.309921	0.419184	-3.095093
9	6	0	-0.877489	2.235965	-0.552722
10	7	0	6.368504	-0.353168	-3.714703
11	8	0	2.306808	-0.898885	-2.150166
12	6	0	-0.933542	1.648725	0.714035
13	6	0	7.389160	0.262436	-4.383768
14	8	0	7.406975	1.473169	-4.595573
15	6	0	-0.000808	0.669287	1.071949
16	6	0	8.515248	-0.659367	-4.848840
17	6	0	0.943769	0.237235	0.142178
18	6	0	9.890741	-0.040604	-4.566939
19	6	0	7.769047	-1.492591	-0.075349
20	6	0	7.826669	-3.019723	-0.193908
21	6	0	6.444998	-3.668401	-0.364016
22	6	0	5.746950	-3.189206	-1.632342
23	8	0	6.353721	-3.091940	-2.707179
24	7	0	4.434486	-2.893602	-1.502050
25	6	0	4.421445	1.754600	3.178378
26	6	0	3.264939	1.156827	3.992784
27	8	0	2.302905	1.828231	4.386869
28	6	0	4.321218	1.327703	1.687711
29	8	0	4.797538	-0.031159	1.649310
30	6	0	5.201693	2.200969	0.799707
31	7	0	3.367444	-0.185772	4.193203
32	6	0	2.282920	-1.062184	4.669799
33	6	0	2.764797	-2.520382	4.656034
34	6	0	3.449403	-2.966298	3.345962
35	6	0	2.629824	-2.712395	2.083430
36	8	0	1.396308	-3.054869	2.163670
37	8	0	3.156918	-2.225850	1.058184
38	6	0	-1.021724	3.146763	5.711673
39	6	0	-1.086736	2.163382	4.538588
40	8	0	-0.407664	0.935280	4.784646
41	6	0	-1.647660	-4.189498	4.697826
42	6	0	-1.837358	-2.667811	4.832101
43	6	0	-2.834736	-2.022352	3.858877
44	6	0	-2.449613	-2.090383	2.385193
45	8	0	-1.312107	-2.527126	2.096984

46	8	0	-3.343027	-1.686444	1.545871
47	6	0	1.879184	-7.240220	3.350130
48	6	0	1.494255	-6.100907	2.393158
49	6	0	0.414609	-6.374016	1.384797
50	7	0	-0.146020	-5.349996	0.620192
51	6	0	-0.119808	-7.564663	0.963273
52	6	0	-0.975633	-5.916863	-0.243540
53	7	0	-0.993831	-7.257009	-0.065504
54	6	0	3.012248	8.002484	3.765342
55	6	0	3.763448	6.675438	3.556686
56	6	0	3.387756	6.018284	2.244624
57	6	0	2.468066	4.960718	2.207446
58	6	0	3.903062	6.496419	1.030389
59	6	0	2.057447	4.411811	0.989701
60	6	0	3.501072	5.946333	-0.187250
61	6	0	2.569437	4.905238	-0.211995
62	6	0	-6.186257	6.999579	-1.230595
63	6	0	-5.867769	7.290020	0.252985
64	6	0	-4.601581	6.675577	0.811429
65	6	0	-3.435824	7.439170	0.962440
66	6	0	-4.578375	5.339488	1.237483
67	6	0	-2.285503	6.888544	1.531027
68	6	0	-3.431788	4.780435	1.806079
69	6	0	-2.280171	5.557821	1.955035
70	6	0	-2.357738	5.474093	-3.855392
71	6	0	-2.140705	3.955115	-3.889971
72	8	0	-1.143977	3.453978	-4.409150
73	6	0	-2.310488	6.139277	-2.465573
74	8	0	-2.824083	7.470784	-2.548142
75	6	0	-0.903327	6.123384	-1.871652
76	6	0	-8.464117	-2.496587	-2.083673
77	6	0	-7.020817	-2.265292	-1.623425
78	6	0	-6.571561	-3.179962	-0.525444
79	7	0	-5.298843	-3.098910	0.031297
80	6	0	-7.265426	-4.160506	0.136132
81	6	0	-5.236213	-3.997612	1.001854
82	7	0	-6.403560	-4.667602	1.093403
83	7	0	-3.140771	3.169875	-3.375455
84	6	0	-3.017582	1.712782	-3.426816
85	6	0	-4.270465	0.982652	-2.922569
86	6	0	-4.414023	1.070784	-1.405532
87	8	0	-4.489039	2.156349	-0.836404
88	8	0	-4.464583	-0.071520	-0.742898
89	6	0	-4.779564	-5.518311	-3.634959
90	6	0	-3.710774	-4.986303	-4.600517
91	6	0	-3.536285	-3.458718	-4.505016
92	6	0	-2.929935	-3.090090	-3.149355
93	8	0	-1.786597	-3.488001	-2.883795
94	8	0	-3.717272	-2.405311	-2.362455
95	8	0	-4.049592	1.406395	2.026216
96	8	0	0.726177	-2.995047	-1.304367
97	8	0	-1.659804	-1.477982	-0.707108
98	8	0	-1.790340	-2.761874	-0.406982
99	1	0	8.304372	-3.448646	0.695671

100	1	0	8.444352	-3.297876	-1.054316
101	1	0	6.553483	-4.758005	-0.444018
102	1	0	5.812276	-3.467881	0.508181
103	1	0	3.994262	-2.790517	-0.592581
104	1	0	3.946874	-2.487032	-2.288206
105	1	0	7.404441	-1.045698	-1.005621
106	1	0	3.282804	1.376215	1.338190
107	1	0	5.175484	1.838481	-0.231873
108	1	0	6.240706	2.162978	1.144482
109	1	0	4.861809	3.240009	0.808434
110	1	0	4.165058	-0.637895	1.226150
111	1	0	4.445775	2.835156	3.311521
112	1	0	1.897926	-3.159888	4.844794
113	1	0	3.472544	-2.680165	5.478166
114	1	0	3.620720	-4.049371	3.399123
115	1	0	4.435904	-2.513713	3.214675
116	1	0	1.395232	-0.946075	4.038215
117	1	0	-2.125371	1.914054	4.295462
118	1	0	-0.662620	2.653543	3.648664
119	1	0	0.544559	1.155578	4.782934
120	1	0	-1.266865	4.155950	5.356603
121	1	0	-2.181445	-2.438705	5.847931
122	1	0	-0.873699	-2.163552	4.715630
123	1	0	-3.840173	-2.453062	3.958553
124	1	0	-2.945061	-0.957599	4.101020
125	1	0	-1.181571	-4.449808	3.745432
126	1	0	1.216357	-5.213647	2.966474
127	1	0	2.387708	-5.803584	1.826942
128	1	0	0.047960	-8.579062	1.286761
129	1	0	-1.525488	-7.915842	-0.613788
130	1	0	-1.537780	-5.396369	-1.008030
131	1	0	2.077454	-8.178208	2.819639
132	1	0	3.535081	5.993625	4.383705
133	1	0	4.844649	6.860525	3.590408
134	1	0	4.626726	7.308791	1.040780
135	1	0	2.083562	4.546279	3.136437
136	1	0	3.916800	6.327095	-1.116251
137	1	0	1.340285	3.596816	0.982712
138	1	0	2.256957	4.472725	-1.157861
139	1	0	1.932187	7.849000	3.668517
140	1	0	-6.717460	6.946884	0.857656
141	1	0	-5.820968	8.377613	0.386676
142	1	0	-3.433922	8.476171	0.636437
143	1	0	-5.474302	4.730748	1.138470
144	1	0	-1.395517	7.501068	1.650878
145	1	0	-3.451060	3.749444	2.147038
146	1	0	-1.385201	5.132060	2.400378
147	1	0	-5.417371	7.402798	-1.895331
148	1	0	-2.998964	5.631425	-1.780916
149	1	0	-0.211083	6.680658	-2.516333
150	1	0	-0.514655	5.104714	-1.783925
151	1	0	-0.905482	6.577934	-0.879704
152	1	0	-2.189971	7.995746	-3.056053
153	1	0	-1.568589	5.895115	-4.486421

154	1	0	-6.325628	-2.366635	-2.464411
155	1	0	-6.904259	-1.233394	-1.270011
156	1	0	-8.272997	-4.524990	0.021994
157	1	0	-6.616442	-5.389098	1.765659
158	1	0	-4.388037	-4.170124	1.644972
159	1	0	-9.169407	-2.414888	-1.249747
160	1	0	-4.214856	-0.066406	-3.220053
161	1	0	-5.168874	1.421101	-3.374913
162	1	0	-2.153227	1.382194	-2.839738
163	1	0	3.629605	-0.451614	-4.210529
164	1	0	4.277354	1.055205	-4.903913
165	1	0	0.104065	2.281750	-2.471444
166	1	0	5.046671	-0.046095	-2.140915
167	1	0	5.695612	1.423272	-2.901564
168	1	0	-1.608630	2.990071	-0.823378
169	1	0	-1.726399	1.921219	1.403540
170	1	0	-0.026871	0.247868	2.072581
171	1	0	8.420859	-1.646945	-4.384698
172	1	0	8.392225	-0.800104	-5.930707
173	1	0	1.664423	-0.533623	0.400304
174	1	0	10.689601	-0.647343	-5.004246
175	1	0	10.073990	0.036047	-3.490171
176	1	0	9.938482	0.966387	-4.987617
177	1	0	-4.715099	-5.016417	-2.662794
178	1	0	-3.965198	-5.263461	-5.629841
179	1	0	-2.739583	-5.443037	-4.381591
180	1	0	-4.491731	-2.944348	-4.644745
181	1	0	-2.842377	-3.118567	-5.281394
182	1	0	-4.284034	1.711316	1.131624
183	1	0	-3.888687	0.459350	1.905587
184	1	0	8.763830	-1.084750	0.105380
185	1	0	7.107271	-1.163313	0.733116
186	1	0	5.368389	1.275759	3.427913
187	1	0	1.997691	-0.783923	5.684049
188	1	0	2.805725	-6.948661	3.844911
189	1	0	1.110302	-7.425925	4.105554
190	1	0	-1.015312	-4.555210	5.507068
191	1	0	-2.607050	-4.719259	4.771564
192	1	0	-4.660102	-6.585278	-3.446430
193	1	0	-5.790755	-5.348090	-4.019346
194	1	0	-8.592644	-3.479878	-2.547767
195	1	0	-8.732984	-1.734390	-2.815116
196	1	0	-2.797542	1.419282	-4.453306
197	1	0	-6.283449	5.924595	-1.416063
198	1	0	-7.145685	7.475692	-1.434014
199	1	0	-3.312915	5.691486	-4.333317
200	1	0	3.306062	8.743441	3.014650
201	1	0	3.195679	8.426270	4.752757
202	1	0	-0.005049	3.182633	6.117240
203	1	0	-1.717129	2.933393	6.523550
204	1	0	4.093592	-0.611451	3.629658
205	1	0	6.404551	-1.355037	-3.525234
206	1	0	-3.820833	3.555361	-2.732596
207	1	0	-0.025957	-3.049177	-1.929177

208	1	0	1.284961	-2.220971	-1.560286

React$_D$

E(B3LYP*/LB)= -5205.884885 Number of imaginary frequencies: 10

Center Number	Atomic Number	Atomic Type	Coordinates (Angstroms) X	Y	Z
1	26	0	0.018426	-3.330115	0.590546
2	26	0	-3.731818	-1.931794	-0.485013
3	6	0	2.025805	0.335935	-2.094191
4	16	0	2.864113	1.558564	-3.074462
5	6	0	1.003736	0.869148	-1.156523
6	6	0	4.158966	0.546716	-3.891906
7	6	0	0.148747	1.926527	-1.502926
8	6	0	5.404891	0.424733	-2.997279
9	6	0	-0.838710	2.331206	-0.601998
10	7	0	6.463476	-0.356568	-3.606029
11	8	0	2.294523	-0.868757	-2.155191
12	6	0	-0.957110	1.716860	0.647317
13	6	0	7.510015	0.249093	-4.243732
14	8	0	7.557432	1.462356	-4.435069
15	6	0	-0.065371	0.703846	1.014715
16	6	0	8.625489	-0.688682	-4.703638
17	6	0	0.900096	0.265280	0.110122
18	6	0	10.009272	-0.097938	-4.402370
19	6	0	7.784621	-1.569265	0.032744
20	6	0	7.803149	-3.094927	-0.104824
21	6	0	6.402455	-3.697305	-0.297142
22	6	0	5.732703	-3.191596	-1.571366
23	8	0	6.356025	-3.107728	-2.637559
24	7	0	4.424963	-2.864275	-1.458181
25	6	0	4.408770	1.683208	3.251910
26	6	0	3.219799	1.080733	4.011431
27	8	0	2.224330	1.741422	4.335755
28	6	0	4.351485	1.289430	1.747706
29	8	0	4.826953	-0.064740	1.681427
30	6	0	5.261398	2.183085	0.910847
31	7	0	3.323587	-0.256940	4.243596
32	6	0	2.211635	-1.124210	4.674100
33	6	0	2.660768	-2.591956	4.626684
34	6	0	3.377126	-3.015612	3.324191
35	6	0	2.593966	-2.750199	2.037438
36	8	0	1.399375	-3.205181	2.031477
37	8	0	3.125308	-2.155868	1.071597
38	6	0	-1.067793	3.110314	5.692023
39	6	0	-1.160137	2.178212	4.480037
40	8	0	-0.531923	0.913819	4.684312
41	6	0	-1.752054	-4.208798	4.595223
42	6	0	-1.741197	-2.671404	4.706377
43	6	0	-2.655355	-1.884604	3.759992
44	6	0	-2.323665	-2.008649	2.282324
45	8	0	-1.203621	-2.472949	1.965929

46	8	0	-3.212568	-1.597380	1.451998
47	6	0	1.767690	-7.284753	3.287028
48	6	0	1.481306	-6.183994	2.262088
49	6	0	0.477857	-6.490137	1.192649
50	7	0	-0.061031	-5.484172	0.390685
51	6	0	0.007560	-7.696984	0.744134
52	6	0	-0.818556	-6.079541	-0.519854
53	7	0	-0.805955	-7.416365	-0.340596
54	6	0	3.055181	7.940135	3.871810
55	6	0	3.795787	6.607245	3.663090
56	6	0	3.440809	5.971159	2.334966
57	6	0	2.506286	4.928518	2.263619
58	6	0	3.991079	6.455983	1.138850
59	6	0	2.115446	4.400574	1.030226
60	6	0	3.608839	5.926840	-0.094292
61	6	0	2.662377	4.900620	-0.153145
62	6	0	-6.053878	7.084638	-1.311944
63	6	0	-5.744491	7.381958	0.171978
64	6	0	-4.511121	6.724570	0.754067
65	6	0	-3.326233	7.449780	0.940222
66	6	0	-4.539642	5.384338	1.166771
67	6	0	-2.207613	6.857765	1.530337
68	6	0	-3.425387	4.784029	1.756843
69	6	0	-2.254027	5.523671	1.940831
70	6	0	-2.191187	5.542552	-3.876269
71	6	0	-2.000399	4.020257	-3.914324
72	8	0	-0.995544	3.508743	-4.407345
73	6	0	-2.162023	6.203282	-2.483713
74	8	0	-2.673394	7.535636	-2.568229
75	6	0	-0.763340	6.184942	-1.870290
76	6	0	-8.415889	-2.377698	-2.301182
77	6	0	-7.105435	-1.983505	-1.619870
78	6	0	-6.841747	-2.705772	-0.339358
79	7	0	-5.588774	-2.691544	0.249736
80	6	0	-7.694921	-3.399861	0.480911
81	6	0	-5.681532	-3.347639	1.396747
82	7	0	-6.940927	-3.794996	1.571764
83	7	0	-3.027100	3.241904	-3.439806
84	6	0	-2.899463	1.784782	-3.497124
85	6	0	-4.129238	1.022859	-2.980367
86	6	0	-4.209175	1.040972	-1.453624
87	8	0	-4.353304	2.106969	-0.855438
88	8	0	-4.115660	-0.110149	-0.826403
89	6	0	-4.734231	-5.424503	-3.809618
90	6	0	-3.560807	-4.867722	-4.627492
91	6	0	-3.458632	-3.325532	-4.590464
92	6	0	-2.909283	-2.832133	-3.253222
93	8	0	-1.684313	-2.856888	-3.062831
94	8	0	-3.801073	-2.436092	-2.390753
95	8	0	-4.085048	1.390568	1.975256
96	8	0	0.358062	-2.674557	-1.301489
97	8	0	-2.140495	-3.282547	-0.389840
98	8	0	-3.105550	-4.120541	-0.128331
99	1	0	8.256819	-3.549811	0.784465

100	1	0	8.421220	-3.381708	-0.962088
101	1	0	6.476667	-4.789434	-0.382662
102	1	0	5.766513	-3.482084	0.569192
103	1	0	3.979006	-2.734725	-0.553992
104	1	0	3.959370	-2.440010	-2.248975
105	1	0	7.444365	-1.103782	-0.897878
106	1	0	3.324160	1.353513	1.368088
107	1	0	5.263088	1.848473	-0.130235
108	1	0	6.289858	2.128681	1.284299
109	1	0	4.927434	3.223584	0.939462
110	1	0	4.162745	-0.676508	1.312744
111	1	0	4.436397	2.760618	3.409876
112	1	0	1.776302	-3.219473	4.769961
113	1	0	3.339655	-2.794451	5.463690
114	1	0	3.547509	-4.098787	3.370694
115	1	0	4.365646	-2.558810	3.227704
116	1	0	1.344511	-0.968356	4.021604
117	1	0	-2.204090	1.977711	4.216395
118	1	0	-0.705721	2.682845	3.614113
119	1	0	0.428901	1.093369	4.686437
120	1	0	-1.265337	4.142023	5.374131
121	1	0	-2.019659	-2.390367	5.728905
122	1	0	-0.718792	-2.312098	4.559959
123	1	0	-3.714039	-2.137494	3.898109
124	1	0	-2.565222	-0.816015	3.998000
125	1	0	-1.337886	-4.553926	3.645043
126	1	0	1.177269	-5.272138	2.778375
127	1	0	2.419643	-5.921119	1.754203
128	1	0	0.183125	-8.705233	1.081723
129	1	0	-1.306474	-8.088346	-0.902042
130	1	0	-1.380706	-5.579456	-1.292223
131	1	0	1.946990	-8.258649	2.817733
132	1	0	3.542022	5.918034	4.476518
133	1	0	4.878009	6.779472	3.722121
134	1	0	4.726515	7.256944	1.175850
135	1	0	2.094075	4.508729	3.178161
136	1	0	4.051742	6.312057	-1.008786
137	1	0	1.385914	3.597275	0.996948
138	1	0	2.365713	4.484110	-1.111173
139	1	0	1.975612	7.799592	3.752250
140	1	0	-6.614616	7.078554	0.768988
141	1	0	-5.657956	8.468412	0.293930
142	1	0	-3.283987	8.489002	0.624216
143	1	0	-5.451511	4.804969	1.040370
144	1	0	-1.301919	7.440656	1.677225
145	1	0	-3.484789	3.750459	2.085444
146	1	0	-1.383578	5.066057	2.402802
147	1	0	-5.271900	7.469190	-1.972352
148	1	0	-2.860778	5.693559	-1.810996
149	1	0	-0.063328	6.748036	-2.501365
150	1	0	-0.373958	5.166554	-1.784417
151	1	0	-0.779681	6.632641	-0.875275
152	1	0	-2.032469	8.061741	-3.066311
153	1	0	-1.384131	5.952173	-4.491729

154	1	0	-6.258770	-2.144554	-2.294956
155	1	0	-7.112952	-0.907433	-1.400098
156	1	0	-8.742696	-3.635288	0.395577
157	1	0	-7.273385	-4.314559	2.370034
158	1	0	-4.875948	-3.512870	2.092246
159	1	0	-9.250304	-2.412019	-1.592537
160	1	0	-4.076724	-0.010685	-3.327753
161	1	0	-5.049701	1.469719	-3.375765
162	1	0	-2.026562	1.466990	-2.915398
163	1	0	3.724860	-0.426647	-4.130016
164	1	0	4.402314	1.067874	-4.820751
165	1	0	0.207818	2.387220	-2.485431
166	1	0	5.125321	-0.038110	-2.046241
167	1	0	5.799333	1.424049	-2.797151
168	1	0	-1.540253	3.109510	-0.880245
169	1	0	-1.764230	1.996106	1.316770
170	1	0	-0.131374	0.266314	2.006114
171	1	0	8.508004	-1.678282	-4.249073
172	1	0	8.511683	-0.818796	-5.787900
173	1	0	1.592331	-0.527344	0.378435
174	1	0	10.801165	-0.715582	-4.837109
175	1	0	10.182863	-0.033986	-3.323197
176	1	0	10.079753	0.911661	-4.813461
177	1	0	-4.789521	-4.938054	-2.829477
178	1	0	-3.652775	-5.188464	-5.671569
179	1	0	-2.610443	-5.278512	-4.264964
180	1	0	-4.439336	-2.877232	-4.778480
181	1	0	-2.764504	-2.990061	-5.366477
182	1	0	-4.253097	1.706461	1.067837
183	1	0	-3.824913	0.468175	1.836363
184	1	0	8.779946	-1.173991	0.236774
185	1	0	7.112867	-1.234912	0.830958
186	1	0	5.345470	1.191645	3.515193
187	1	0	1.909320	-0.852534	5.685148
188	1	0	2.687472	-7.007972	3.802483
189	1	0	0.960590	-7.396963	4.016320
190	1	0	-1.139651	-4.588972	5.412963
191	1	0	-2.761659	-4.622498	4.711411
192	1	0	-4.629732	-6.494390	-3.629004
193	1	0	-5.689568	-5.253437	-4.316948
194	1	0	-8.350380	-3.346847	-2.803189
195	1	0	-8.662222	-1.605782	-3.030261
196	1	0	-2.662476	1.498583	-4.521854
197	1	0	-6.167400	6.009753	-1.488844
198	1	0	-7.004025	7.573022	-1.529381
199	1	0	-3.134466	5.774828	-4.370594
200	1	0	3.371502	8.685243	3.134480
201	1	0	3.223713	8.352522	4.866622
202	1	0	-0.055286	3.087145	6.108650
203	1	0	-1.781210	2.896832	6.488084
204	1	0	4.085683	-0.691717	3.739445
205	1	0	6.475451	-1.361812	-3.433349
206	1	0	-3.722804	3.628891	-2.815320
207	1	0	-0.395221	-2.643709	-1.944992

208	1	0	1.067661	-2.045810	-1.571989

TS1$_{SR}$

E(B3LYP*/LB)= -5205.872794 Number of imaginary frequencies: 7

Center Number	Atomic Number	Atomic Type	Coordinates (Angstroms) X	Y	Z
1	26	0	-0.638645	-3.085697	0.704840
2	26	0	-3.333499	-1.586743	-0.553641
3	6	0	1.938021	0.078868	-2.277439
4	16	0	2.791408	1.261418	-3.302067
5	6	0	0.901371	0.644500	-1.366389
6	6	0	4.105051	0.209630	-4.036122
7	6	0	0.201450	1.828381	-1.613515
8	6	0	5.336953	0.137364	-3.116781
9	6	0	-0.807733	2.251733	-0.722940
10	7	0	6.399549	-0.681352	-3.666428
11	8	0	2.195715	-1.123931	-2.281720
12	6	0	-1.129011	1.507513	0.399418
13	6	0	7.481038	-0.115885	-4.284423
14	8	0	7.565603	1.090756	-4.501109
15	6	0	-0.393431	0.331188	0.686121
16	6	0	8.580726	-1.095660	-4.694229
17	6	0	0.632028	-0.080077	-0.194019
18	6	0	9.977433	-0.513030	-4.444660
19	6	0	7.727551	-1.874342	0.012830
20	6	0	7.687958	-3.401333	-0.105369
21	6	0	6.264757	-3.954958	-0.271939
22	6	0	5.596928	-3.435486	-1.541338
23	8	0	6.202932	-3.401237	-2.620048
24	7	0	4.312713	-3.035449	-1.410470
25	6	0	4.521327	1.529824	3.248410
26	6	0	3.340365	0.963736	4.051446
27	8	0	2.393071	1.666707	4.425515
28	6	0	4.393320	1.139064	1.750962
29	8	0	4.753117	-0.250349	1.673987
30	6	0	5.340383	1.958751	0.880139
31	7	0	3.388068	-0.383152	4.269412
32	6	0	2.216739	-1.176095	4.697094
33	6	0	2.468069	-2.675938	4.531306
34	6	0	3.020001	-3.097128	3.147262
35	6	0	2.222543	-2.688749	1.900337
36	8	0	0.977916	-3.008169	1.873870
37	8	0	2.804248	-2.105296	0.960695
38	6	0	-0.868630	3.198823	5.727659
39	6	0	-0.950543	2.181704	4.584141
40	8	0	-0.345559	0.932018	4.897187
41	6	0	-1.876639	-4.087092	4.659848
42	6	0	-2.244829	-2.624393	4.966179
43	6	0	-3.218846	-1.970212	3.976757
44	6	0	-2.725991	-1.918647	2.536830
45	8	0	-1.568921	-2.422823	2.339097

46	8	0	-3.476238	-1.413198	1.655664
47	6	0	1.496633	-7.315680	3.333346
48	6	0	1.023537	-6.086311	2.536039
49	6	0	-0.084647	-6.330200	1.550986
50	7	0	-0.732331	-5.304413	0.857600
51	6	0	-0.570764	-7.527615	1.092570
52	6	0	-1.560099	-5.881525	-0.002987
53	7	0	-1.500068	-7.223942	0.113262
54	6	0	3.443730	7.841114	3.859008
55	6	0	4.005645	6.416415	3.704085
56	6	0	3.588831	5.773843	2.396150
57	6	0	2.623885	4.757278	2.367849
58	6	0	4.125756	6.216235	1.177230
59	6	0	2.194280	4.211880	1.154558
60	6	0	3.703569	5.670397	-0.035252
61	6	0	2.729782	4.668102	-0.051138
62	6	0	-5.735562	7.364412	-1.248916
63	6	0	-5.434928	7.572805	0.251039
64	6	0	-4.182500	6.909102	0.784138
65	6	0	-3.009375	7.648016	0.992089
66	6	0	-4.177806	5.548433	1.124509
67	6	0	-1.870188	7.049923	1.534329
68	6	0	-3.042790	4.942764	1.668594
69	6	0	-1.883840	5.695680	1.876074
70	6	0	-1.963918	5.649840	-3.839107
71	6	0	-1.814010	4.125129	-3.827686
72	8	0	-0.789913	3.575556	-4.238329
73	6	0	-1.887394	6.339970	-2.460058
74	8	0	-2.358210	7.684770	-2.562820
75	6	0	-0.477151	6.288503	-1.875345
76	6	0	-8.511160	-1.990005	-2.188832
77	6	0	-7.046141	-1.781792	-1.742111
78	6	0	-6.539019	-2.760409	-0.724988
79	7	0	-5.227041	-2.766795	-0.249204
80	6	0	-7.228605	-3.768192	-0.100200
81	6	0	-5.144356	-3.752009	0.630693
82	7	0	-6.331476	-4.383441	0.753955
83	7	0	-2.876332	3.388568	-3.379726
84	6	0	-2.830338	1.927119	-3.442185
85	6	0	-4.109734	1.244376	-2.937832
86	6	0	-4.214157	1.291959	-1.413065
87	8	0	-4.446492	2.357894	-0.839735
88	8	0	-4.036013	0.166747	-0.762928
89	6	0	-4.976600	-5.197076	-3.716842
90	6	0	-3.805376	-4.739228	-4.604897
91	6	0	-3.514584	-3.228276	-4.527534
92	6	0	-2.773476	-2.846593	-3.238593
93	8	0	-1.685149	-3.389618	-2.998962
94	8	0	-3.368872	-1.951580	-2.505008
95	8	0	-4.008659	1.636446	1.970947
96	8	0	0.373934	-3.344577	-1.192452
97	8	0	-1.325709	-1.412186	-0.177891
98	8	0	-2.255521	-3.117114	-0.223919
99	1	0	8.137618	-3.861172	0.783290

100	1	0	8.283046	-3.721101	-0.967235
101	1	0	6.297908	-5.049512	-0.348612
102	1	0	5.651449	-3.710706	0.603507
103	1	0	3.878113	-2.885951	-0.506277
104	1	0	3.839769	-2.622329	-2.203113
105	1	0	7.398022	-1.406076	-0.920148
106	1	0	3.360753	1.282907	1.406070
107	1	0	5.282083	1.624261	-0.159549
108	1	0	6.373100	1.824658	1.219522
109	1	0	5.089743	3.022406	0.915969
110	1	0	4.045482	-0.782117	1.265285
111	1	0	4.593406	2.604642	3.405767
112	1	0	1.519977	-3.190120	4.716957
113	1	0	3.179441	-3.029408	5.287602
114	1	0	3.082507	-4.191485	3.134710
115	1	0	4.041827	-2.737047	3.001096
116	1	0	1.356591	-0.892112	4.075695
117	1	0	-1.994151	1.979949	4.318518
118	1	0	-0.476309	2.616666	3.689583
119	1	0	0.616591	1.097371	4.887708
120	1	0	-1.080335	4.204841	5.343584
121	1	0	-2.700470	-2.570160	5.962664
122	1	0	-1.338366	-2.011624	5.010390
123	1	0	-4.186647	-2.491025	3.960168
124	1	0	-3.443763	-0.940960	4.280573
125	1	0	-1.311252	-4.172487	3.735233
126	1	0	0.724617	-5.285762	3.218081
127	1	0	1.870218	-5.684187	1.967113
128	1	0	-0.337297	-8.544034	1.364040
129	1	0	-2.013851	-7.885410	-0.448808
130	1	0	-2.172250	-5.350307	-0.717421
131	1	0	1.703994	-8.166545	2.675134
132	1	0	3.658078	5.795317	4.537154
133	1	0	5.100635	6.449647	3.774521
134	1	0	4.883613	6.996725	1.179420
135	1	0	2.221025	4.368236	3.299614
136	1	0	4.136477	6.023121	-0.967347
137	1	0	1.442521	3.427718	1.155648
138	1	0	2.405319	4.236959	-0.993874
139	1	0	2.358551	7.842162	3.711238
140	1	0	-6.296271	7.211530	0.827784
141	1	0	-5.371584	8.650769	0.443245
142	1	0	-2.993567	8.704082	0.734159
143	1	0	-5.077526	4.955370	0.978621
144	1	0	-0.975012	7.643913	1.700276
145	1	0	-3.080507	3.894741	1.950896
146	1	0	-0.998060	5.233676	2.303568
147	1	0	-4.932636	7.758053	-1.878478
148	1	0	-2.587595	5.864470	-1.764363
149	1	0	0.222737	6.830401	-2.525030
150	1	0	-0.112166	5.260198	-1.798251
151	1	0	-0.459201	6.739094	-0.881836
152	1	0	-1.706316	8.184472	-3.073641
153	1	0	-1.148619	6.015945	-4.471635

154	1	0	-6.383769	-1.822072	-2.615254
155	1	0	-6.930385	-0.769586	-1.334423
156	1	0	-8.254293	-4.088349	-0.182885
157	1	0	-6.529527	-5.151081	1.377275
158	1	0	-4.255249	-4.021160	1.178024
159	1	0	-9.211318	-1.890248	-1.353849
160	1	0	-4.092655	0.203345	-3.264454
161	1	0	-4.994035	1.736403	-3.359786
162	1	0	-1.982398	1.555546	-2.860205
163	1	0	3.675346	-0.775838	-4.227881
164	1	0	4.362163	0.678752	-4.988645
165	1	0	0.375597	2.404256	-2.518981
166	1	0	5.040227	-0.268032	-2.144809
167	1	0	5.735188	1.144243	-2.969981
168	1	0	-1.360676	3.159130	-0.938194
169	1	0	-1.945742	1.795571	1.056342
170	1	0	-0.495665	-0.135439	1.658576
171	1	0	8.450272	-2.053351	-4.178712
172	1	0	8.451896	-1.293716	-5.766690
173	1	0	1.267445	-0.920956	0.059225
174	1	0	10.752689	-1.166511	-4.856196
175	1	0	10.168070	-0.393386	-3.373160
176	1	0	10.058249	0.471828	-4.910128
177	1	0	-4.946809	-4.700646	-2.739609
178	1	0	-4.009310	-5.016591	-5.645619
179	1	0	-2.887073	-5.263028	-4.318234
180	1	0	-4.434971	-2.642974	-4.612976
181	1	0	-2.858304	-2.945348	-5.359075
182	1	0	-4.273439	1.933923	1.079974
183	1	0	-3.950150	0.673588	1.871212
184	1	0	8.740634	-1.521791	0.207581
185	1	0	7.076310	-1.506283	0.813539
186	1	0	5.438077	0.999186	3.505792
187	1	0	1.934850	-0.888729	5.709711
188	1	0	2.431469	-7.077360	3.840730
189	1	0	0.759555	-7.630562	4.077871
190	1	0	-1.274510	-4.490957	5.473917
191	1	0	-2.774663	-4.715002	4.579777
192	1	0	-4.916922	-6.270001	-3.533740
193	1	0	-5.946499	-4.970906	-4.171188
194	1	0	-8.660087	-2.965518	-2.661848
195	1	0	-8.729941	-1.210352	-2.918436
196	1	0	-2.614298	1.628199	-4.467949
197	1	0	-5.876137	6.303940	-1.483821
198	1	0	-6.665506	7.892695	-1.460330
199	1	0	-2.900259	5.921188	-4.326614
200	1	0	3.876035	8.521862	3.118533
201	1	0	3.637996	8.248698	4.851153
202	1	0	0.141785	3.212681	6.149662
203	1	0	-1.584089	3.018586	6.530086
204	1	0	4.099303	-0.851683	3.721592
205	1	0	6.391560	-1.680066	-3.458604
206	1	0	-3.639485	3.822675	-2.878122
207	1	0	-0.385515	-3.290624	-1.837114

208	1	0	0.995908	-2.639359	-1.454861

TS1$_{RS}$

E(B3LYP*/LB)= -5205.865656   Number of imaginary frequencies: 9

Center Number	Atomic Number	Atomic Type	Coordinates (Angstroms) X	Y	Z
1	26	0	-1.350471	-2.781166	0.702594
2	26	0	-3.830126	-0.848969	-0.522204
3	6	0	1.840042	-0.740993	-2.370551
4	16	0	3.056198	0.252881	-3.184351
5	6	0	1.085847	-0.000078	-1.315708
6	6	0	4.098900	-1.022415	-3.988511
7	6	0	-0.280599	0.249768	-1.442119
8	6	0	5.253222	-1.411443	-3.051082
9	6	0	-0.974700	0.837161	-0.358572
10	7	0	6.105832	-2.441498	-3.609470
11	8	0	1.617525	-1.925300	-2.614213
12	6	0	-0.300008	1.148074	0.844898
13	6	0	7.156205	-2.102461	-4.410963
14	8	0	7.361847	-0.942969	-4.767319
15	6	0	1.057568	0.912291	0.946916
16	6	0	8.093895	-3.238332	-4.801941
17	6	0	1.753127	0.356294	-0.142466
18	6	0	9.537728	-2.874589	-4.428783
19	6	0	7.058621	-3.592543	0.057053
20	6	0	6.584615	-5.043409	0.167736
21	6	0	5.058110	-5.167386	0.057101
22	6	0	4.530991	-4.674497	-1.288390
23	8	0	5.118362	-4.932003	-2.345490
24	7	0	3.372026	-3.975327	-1.243422
25	6	0	4.766316	0.525210	3.239379
26	6	0	3.412764	0.205710	3.848366
27	8	0	2.511702	1.057130	3.875176
28	6	0	4.762863	0.520011	1.690893
29	8	0	4.646999	-0.803464	1.175966
30	6	0	6.074287	1.079388	1.149688
31	7	0	3.217496	-1.055671	4.308259
32	6	0	1.897807	-1.536278	4.740711
33	6	0	1.744299	-3.047038	4.556167
34	6	0	2.203877	-3.563390	3.173430
35	6	0	1.554788	-2.949180	1.918491
36	8	0	0.283553	-3.105104	1.793557
37	8	0	2.282661	-2.390624	1.075871
38	6	0	-0.062170	3.459515	5.700981
39	6	0	-0.471624	2.596560	4.502903
40	8	0	-0.254717	1.203980	4.714104
41	6	0	-2.766782	-3.399646	4.764018
42	6	0	-2.665640	-1.881641	4.999396
43	6	0	-3.453404	-1.001491	4.020620
44	6	0	-3.066566	-1.159437	2.556143
45	8	0	-2.039278	-1.883728	2.333932

46	8	0	-3.778581	-0.581023	1.686261
47	6	0	-0.258615	-7.354445	3.487042
48	6	0	-0.422295	-6.068051	2.654816
49	6	0	-1.567116	-6.065776	1.682425
50	7	0	-1.957765	-4.939848	0.952734
51	6	0	-2.335594	-7.127213	1.277242
52	6	0	-2.915267	-5.333241	0.124359
53	7	0	-3.179843	-6.645495	0.293286
54	6	0	5.211400	6.921752	3.741077
55	6	0	5.600204	5.451975	3.510242
56	6	0	5.153009	4.975977	2.143321
57	6	0	3.896554	4.379389	1.965828
58	6	0	5.952234	5.193497	1.011264
59	6	0	3.443866	4.027923	0.691872
60	6	0	5.507557	4.835091	-0.262422
61	6	0	4.247735	4.255465	-0.427369
62	6	0	-3.853587	8.539346	-1.336941
63	6	0	-3.651286	8.522061	0.193021
64	6	0	-2.534148	7.636722	0.705547
65	6	0	-1.313689	8.192664	1.112673
66	6	0	-2.700504	6.247967	0.817953
67	6	0	-0.294367	7.391890	1.630875
68	6	0	-1.684818	5.439621	1.334369
69	6	0	-0.478999	6.012659	1.746448
70	6	0	-0.609250	5.940610	-3.904994
71	6	0	-0.871548	4.425564	-3.959344
72	8	0	-0.108219	3.658793	-4.539074
73	6	0	-0.400713	6.549766	-2.507327
74	8	0	-0.437834	7.976830	-2.585306
75	6	0	0.891134	6.059152	-1.855709
76	6	0	-8.762957	0.089468	-2.105560
77	6	0	-7.306800	-0.104290	-1.651869
78	6	0	-7.121573	-1.146638	-0.590977
79	7	0	-5.869798	-1.501848	-0.096565
80	6	0	-8.067277	-1.892041	0.066202
81	6	0	-6.062006	-2.428212	0.828148
82	7	0	-7.378284	-2.697422	0.957060
83	7	0	-2.050348	3.969901	-3.409873
84	6	0	-2.326667	2.534505	-3.440587
85	6	0	-3.739773	2.139117	-2.964999
86	6	0	-3.898123	2.176171	-1.445353
87	8	0	-3.779516	3.244284	-0.839872
88	8	0	-4.127418	1.037896	-0.828830
89	6	0	-6.094225	-3.885829	-3.584775
90	6	0	-4.908509	-3.756090	-4.556734
91	6	0	-4.247211	-2.365069	-4.558354
92	6	0	-3.496680	-2.121769	-3.246435
93	8	0	-2.461839	-2.767094	-3.025535
94	8	0	-4.043827	-1.240778	-2.461389
95	8	0	-3.187174	2.348914	1.871623
96	8	0	-0.526214	-3.297795	-1.190724
97	8	0	-1.860152	-1.059478	-0.313849
98	8	0	-3.022757	-2.544932	-0.139066
99	1	0	6.901277	-5.477309	1.124231

100	1	0	7.040100	-5.646085	-0.624958
101	1	0	4.759487	-6.220088	0.149683
102	1	0	4.567170	-4.622129	0.871958
103	1	0	3.020571	-3.563190	-0.383978
104	1	0	2.994134	-3.606201	-2.106365
105	1	0	6.871696	-3.203752	-0.948346
106	1	0	3.942734	1.159042	1.339611
107	1	0	6.070376	1.032198	0.057938
108	1	0	6.920669	0.485364	1.511564
109	1	0	6.210939	2.120740	1.451413
110	1	0	3.778608	-1.211312	1.352833
111	1	0	5.059720	1.514352	3.600374
112	1	0	0.689291	-3.292383	4.713695
113	1	0	2.312982	-3.590767	5.321830
114	1	0	2.012383	-4.641432	3.139330
115	1	0	3.282795	-3.431925	3.057788
116	1	0	1.164475	-1.031773	4.102937
117	1	0	-1.532825	2.727228	4.268272
118	1	0	0.086916	2.935127	3.616517
119	1	0	0.699028	1.061875	4.581896
120	1	0	0.056409	4.503334	5.383565
121	1	0	-3.023434	-1.651672	6.010716
122	1	0	-1.616634	-1.570905	4.968532
123	1	0	-4.534045	-1.182972	4.089717
124	1	0	-3.309083	0.056957	4.270592
125	1	0	-2.300500	-3.690367	3.823823
126	1	0	-0.510974	-5.199542	3.313424
127	1	0	0.491544	-5.898743	2.073092
128	1	0	-2.349591	-8.158985	1.587951
129	1	0	-3.848476	-7.182899	-0.237125
130	1	0	-3.400098	-4.695484	-0.599746
131	1	0	-0.267176	-8.247278	2.852452
132	1	0	5.145393	4.827074	4.288029
133	1	0	6.686879	5.340237	3.609202
134	1	0	6.931895	5.651304	1.130791
135	1	0	3.270067	4.179735	2.832205
136	1	0	6.144484	5.007442	-1.125654
137	1	0	2.464499	3.573307	0.575320
138	1	0	3.897568	3.978185	-1.417288
139	1	0	4.127933	7.050062	3.646375
140	1	0	-4.594130	8.215724	0.664781
141	1	0	-3.464180	9.549420	0.528328
142	1	0	-1.165953	9.267025	1.031811
143	1	0	-3.637133	5.788251	0.511855
144	1	0	0.640206	7.846354	1.949707
145	1	0	-1.847812	4.371139	1.432786
146	1	0	0.311216	5.388982	2.155893
147	1	0	-2.934786	8.819576	-1.860036
148	1	0	-1.245096	6.298092	-1.856768
149	1	0	1.759239	6.334618	-2.468927
150	1	0	0.894336	4.968985	-1.756404
151	1	0	1.005123	6.498640	-0.863225
152	1	0	0.346412	8.261281	-3.074763
153	1	0	0.278916	6.108861	-4.521589

154	1	0	-6.672270	-0.370071	-2.505252
155	1	0	-6.904102	0.842964	-1.273254
156	1	0	-9.141448	-1.917021	-0.015646
157	1	0	-7.785173	-3.352290	1.607180
158	1	0	-5.281869	-2.903295	1.401479
159	1	0	-9.409720	0.382048	-1.271810
160	1	0	-3.954485	1.128609	-3.318780
161	1	0	-4.475602	2.824064	-3.403048
162	1	0	-1.573344	2.000853	-2.850576
163	1	0	3.454385	-1.869330	-4.234344
164	1	0	4.486821	-0.582080	-4.909154
165	1	0	-0.804241	-0.003756	-2.355202
166	1	0	4.858324	-1.760483	-2.093947
167	1	0	5.867073	-0.526102	-2.865491
168	1	0	-1.967335	1.234946	-0.479079
169	1	0	-0.875694	1.592315	1.650449
170	1	0	1.586138	1.155240	1.862155
171	1	0	7.789587	-4.180809	-4.334210
172	1	0	8.017943	-3.370807	-5.887964
173	1	0	2.813106	0.147396	-0.070348
174	1	0	10.240284	-3.632634	-4.788761
175	1	0	9.654586	-2.793402	-3.343014
176	1	0	9.800502	-1.910542	-4.870073
177	1	0	-5.875356	-3.402406	-2.626608
178	1	0	-5.238976	-3.998832	-5.573503
179	1	0	-4.133547	-4.487194	-4.299532
180	1	0	-4.992215	-1.578960	-4.714536
181	1	0	-3.514381	-2.315959	-5.370795
182	1	0	-3.501809	2.744648	1.038177
183	1	0	-3.526659	1.441931	1.831655
184	1	0	8.127483	-3.485739	0.243311
185	1	0	6.529989	-2.937582	0.759853
186	1	0	5.533601	-0.202474	3.503999
187	1	0	1.698048	-1.173755	5.748765
188	1	0	0.709280	-7.335010	3.988020
189	1	0	-1.045813	-7.460976	4.239321
190	1	0	-2.271758	-3.920578	5.583742
191	1	0	-3.813232	-3.733133	4.752469
192	1	0	-6.288113	-4.939359	-3.382931
193	1	0	-7.004596	-3.429176	-3.986173
194	1	0	-9.171997	-0.819847	-2.558041
195	1	0	-8.796327	0.886517	-2.848300
196	1	0	-2.193707	2.176058	-4.461392
197	1	0	-4.175434	7.561387	-1.710045
198	1	0	-4.634078	9.268485	-1.555522
199	1	0	-1.458253	6.417089	-4.395112
200	1	0	5.680848	7.566896	2.990857
201	1	0	5.502652	7.288424	4.725427
202	1	0	0.901268	3.124117	6.099657
203	1	0	-0.794837	3.466495	6.508065
204	1	0	4.000370	-1.692176	4.305735
205	1	0	5.918133	-3.407349	-3.348392
206	1	0	-2.534824	4.515911	-2.708324
207	1	0	-1.253913	-3.056796	-1.829061

208	1	0	0.272460	-2.840726	-1.525608

Prod$_{SR}$

E(B3LYP*/LB)= -5205.936099 Number of imaginary frequencies: 7

Center Number	Atomic Number	Atomic Type	Coordinates (Angstroms) X	Y	Z
1	26	0	-0.981184	-2.896455	0.632150
2	26	0	-3.713763	-1.421610	-0.564576
3	6	0	2.010759	-0.035142	-2.171908
4	16	0	3.005350	1.068629	-3.152961
5	6	0	1.041409	0.600096	-1.250521
6	6	0	4.163494	-0.113040	-3.949651
7	6	0	0.405638	1.778947	-1.483765
8	6	0	5.378306	-0.385985	-3.044345
9	6	0	-0.546662	2.322550	-0.530821
10	7	0	6.320606	-1.320298	-3.628604
11	8	0	2.128493	-1.263082	-2.209113
12	6	0	-0.837733	1.699630	0.633966
13	6	0	7.464300	-0.883953	-4.238785
14	8	0	7.704522	0.307111	-4.423890
15	6	0	-0.226369	0.395265	0.955857
16	6	0	8.427819	-1.985562	-4.680500
17	6	0	0.735328	-0.175099	-0.027329
18	6	0	9.888075	-1.599442	-4.409656
19	6	0	7.470833	-2.731682	0.024221
20	6	0	7.261841	-4.242586	-0.118198
21	6	0	5.786891	-4.628172	-0.309538
22	6	0	5.197578	-4.013216	-1.575213
23	8	0	5.820275	-4.023592	-2.645525
24	7	0	3.962402	-3.479744	-1.450877
25	6	0	4.626251	0.982829	3.256569
26	6	0	3.374244	0.540852	4.024327
27	8	0	2.483801	1.336868	4.351699
28	6	0	4.490583	0.608465	1.752483
29	8	0	4.730860	-0.804697	1.673441
30	6	0	5.522725	1.345345	0.904804
31	7	0	3.283302	-0.799927	4.258682
32	6	0	2.032254	-1.465549	4.673731
33	6	0	2.126443	-2.985707	4.525555
34	6	0	2.657506	-3.478704	3.159182
35	6	0	1.939663	-2.977677	1.895429
36	8	0	0.672261	-3.127939	1.840786
37	8	0	2.630219	-2.475362	0.974706
38	6	0	-0.570755	3.211733	5.707490
39	6	0	-0.788705	2.289865	4.501623
40	8	0	-0.328480	0.957016	4.705586
41	6	0	-2.350873	-3.917538	4.591395
42	6	0	-2.536824	-2.421329	4.903216
43	6	0	-3.421355	-1.642026	3.920826
44	6	0	-2.909013	-1.592023	2.487214
45	8	0	-1.759532	-2.106400	2.287513

46	8	0	-3.639380	-1.055806	1.605158
47	6	0	0.664246	-7.484808	3.270298
48	6	0	0.362822	-6.192704	2.480079
49	6	0	-0.768249	-6.261913	1.492894
50	7	0	-1.274504	-5.148600	0.812184
51	6	0	-1.413679	-7.379491	1.029527
52	6	0	-2.181641	-5.605183	-0.041381
53	7	0	-2.300387	-6.945688	0.060730
54	6	0	4.231299	7.370521	3.895319
55	6	0	4.720806	5.923916	3.702088
56	6	0	4.266712	5.347488	2.376766
57	6	0	3.172912	4.473152	2.309202
58	6	0	4.892212	5.725659	1.178964
59	6	0	2.703409	4.008570	1.077685
60	6	0	4.431139	5.257883	-0.051936
61	6	0	3.328296	4.401560	-0.107373
62	6	0	-4.906968	7.912258	-1.279231
63	6	0	-4.562990	8.141145	0.208825
64	6	0	-3.432894	7.310031	0.778331
65	6	0	-2.154655	7.856701	0.957917
66	6	0	-3.650465	5.985226	1.183997
67	6	0	-1.128049	7.106742	1.535500
68	6	0	-2.629516	5.227871	1.762153
69	6	0	-1.362643	5.790590	1.939899
70	6	0	-1.322890	5.813252	-3.852657
71	6	0	-1.351661	4.280266	-3.839656
72	8	0	-0.385202	3.618783	-4.223553
73	6	0	-1.180035	6.486250	-2.471432
74	8	0	-1.480038	7.879504	-2.574675
75	6	0	0.203138	6.260279	-1.864427
76	6	0	-8.668460	-1.083240	-2.291445
77	6	0	-7.220301	-1.057479	-1.770075
78	6	0	-6.932671	-2.061950	-0.694123
79	7	0	-5.659738	-2.262521	-0.164935
80	6	0	-7.799376	-2.903557	-0.044638
81	6	0	-5.765388	-3.192734	0.772248
82	7	0	-7.043873	-3.609522	0.875303
83	7	0	-2.505659	3.665059	-3.429861
84	6	0	-2.588829	2.203870	-3.482842
85	6	0	-3.897606	1.604211	-2.950995
86	6	0	-3.952762	1.619778	-1.420597
87	8	0	-4.026977	2.700560	-0.829710
88	8	0	-3.914570	0.472223	-0.792127
89	6	0	-5.489096	-4.645635	-3.812899
90	6	0	-4.265015	-4.322275	-4.687044
91	6	0	-3.832874	-2.841801	-4.650562
92	6	0	-3.154276	-2.488001	-3.323924
93	8	0	-2.010053	-2.912387	-3.109266
94	8	0	-3.869426	-1.757300	-2.518175
95	8	0	-3.794850	2.001434	1.972833
96	8	0	-0.034943	-3.083975	-1.246930
97	8	0	-0.625454	-0.713142	0.077209
98	8	0	-2.563685	-2.763822	-0.228290
99	1	0	7.645058	-4.763488	0.767970

100	1	0	7.828158	-4.614454	-0.978460
101	1	0	5.696861	-5.717917	-0.407206
102	1	0	5.193402	-4.331784	0.563372
103	1	0	3.536212	-3.295199	-0.547544
104	1	0	3.555235	-2.994812	-2.239062
105	1	0	7.201971	-2.216955	-0.903778
106	1	0	3.481885	0.845870	1.387931
107	1	0	5.452887	1.023351	-0.137980
108	1	0	6.533051	1.117140	1.261068
109	1	0	5.367761	2.426845	0.944865
110	1	0	3.969295	-1.288392	1.300575
111	1	0	4.807413	2.043741	3.421938
112	1	0	1.123989	-3.391112	4.694296
113	1	0	2.777657	-3.403644	5.303035
114	1	0	2.592430	-4.572848	3.152774
115	1	0	3.718073	-3.241810	3.039138
116	1	0	1.213045	-1.099964	4.041316
117	1	0	-1.851106	2.226050	4.243471
118	1	0	-0.278395	2.732542	3.630881
119	1	0	0.647215	1.009625	4.727833
120	1	0	-0.624762	4.257910	5.380251
121	1	0	-2.980907	-2.315869	5.900870
122	1	0	-1.560884	-1.927519	4.946983
123	1	0	-4.441346	-2.048649	3.882072
124	1	0	-3.532176	-0.602272	4.252560
125	1	0	-1.798974	-4.066541	3.664234
126	1	0	0.180949	-5.364689	3.170517
127	1	0	1.259562	-5.907608	1.916937
128	1	0	-1.316894	-8.419889	1.293346
129	1	0	-2.906137	-7.526240	-0.498825
130	1	0	-2.736233	-4.989549	-0.733213
131	1	0	0.775400	-8.349532	2.607412
132	1	0	4.342214	5.299190	4.519020
133	1	0	5.815930	5.899044	3.768692
134	1	0	5.749686	6.394348	1.212908
135	1	0	2.694650	4.135156	3.225330
136	1	0	4.933667	5.557789	-0.967432
137	1	0	1.847897	3.340241	1.047148
138	1	0	2.970918	4.033758	-1.064815
139	1	0	3.145496	7.427181	3.763883
140	1	0	-5.467486	7.951478	0.801410
141	1	0	-4.326698	9.203039	0.347985
142	1	0	-1.966343	8.881338	0.647470
143	1	0	-4.636405	5.542604	1.062137
144	1	0	-0.147278	7.553160	1.678549
145	1	0	-2.836814	4.212967	2.088383
146	1	0	-0.563917	5.210925	2.394650
147	1	0	-4.074118	8.185775	-1.933045
148	1	0	-1.944265	6.098812	-1.788631
149	1	0	0.977328	6.706144	-2.502434
150	1	0	0.433850	5.194728	-1.776290
151	1	0	0.260357	6.710527	-0.872157
152	1	0	-0.770464	8.293258	-3.085564
153	1	0	-0.466137	6.082286	-4.478660

154	1	0	-6.511886	-1.223138	-2.589718
155	1	0	-6.988351	-0.059167	-1.377012
156	1	0	-8.861120	-3.054179	-0.149433
157	1	0	-7.387652	-4.299189	1.526250
158	1	0	-4.952521	-3.569365	1.372677
159	1	0	-9.389588	-0.916406	-1.484422
160	1	0	-3.972821	0.573899	-3.303692
161	1	0	-4.759866	2.165666	-3.330903
162	1	0	-1.757984	1.772831	-2.912527
163	1	0	3.607037	-1.024527	-4.177850
164	1	0	4.476084	0.359014	-4.883968
165	1	0	0.577857	2.330896	-2.405339
166	1	0	5.032416	-0.783916	-2.085531
167	1	0	5.908316	0.552347	-2.863591
168	1	0	-1.029956	3.264484	-0.766245
169	1	0	-1.550024	2.115037	1.340471
170	1	0	-0.176874	0.105907	2.002529
171	1	0	8.170316	-2.934707	-4.198154
172	1	0	8.281250	-2.127748	-5.759518
173	1	0	1.480002	-0.893787	0.300192
174	1	0	10.572748	-2.338335	-4.837097
175	1	0	10.085025	-1.536114	-3.334512
176	1	0	10.101664	-0.621448	-4.846783
177	1	0	-5.417275	-4.153784	-2.836329
178	1	0	-4.474778	-4.607021	-5.724709
179	1	0	-3.406528	-4.926096	-4.370302
180	1	0	-4.691832	-2.184367	-4.816530
181	1	0	-3.100619	-2.663522	-5.444692
182	1	0	-3.941245	2.318200	1.060190
183	1	0	-3.824430	1.038008	1.868607
184	1	0	8.514529	-2.491485	0.228052
185	1	0	6.857672	-2.304674	0.825545
186	1	0	5.478431	0.355110	3.517326
187	1	0	1.775409	-1.154392	5.686028
188	1	0	1.615564	-7.351320	3.785483
189	1	0	-0.112941	-7.710161	4.006311
190	1	0	-1.802055	-4.387995	5.407368
191	1	0	-3.317924	-4.432230	4.510306
192	1	0	-5.546981	-5.719601	-3.635377
193	1	0	-6.421843	-4.316082	-4.281835
194	1	0	-8.907355	-2.033931	-2.778520
195	1	0	-8.796358	-0.280961	-3.018112
196	1	0	-2.398626	1.888447	-4.508806
197	1	0	-5.177066	6.868273	-1.470754
198	1	0	-5.772874	8.538840	-1.494142
199	1	0	-2.220749	6.186467	-4.345165
200	1	0	4.685671	8.042141	3.159631
201	1	0	4.460916	7.749904	4.891097
202	1	0	0.428437	3.053825	6.127260
203	1	0	-1.307524	3.105863	6.503872
204	1	0	3.966865	-1.349143	3.752661
205	1	0	6.178207	-2.315034	-3.450752
206	1	0	-3.207586	4.168627	-2.904373
207	1	0	-0.756726	-2.948879	-1.920769

208	1	0	0.725118	-2.520370	-1.494501

Prod$_{RS}$

E(B3LYP*/LB)= -5205.920054   Number of imaginary frequencies: 10

Center Number	Atomic Number	Atomic Type	Coordinates (Angstroms) X	Y	Z
1	26	0	-1.773548	-2.768615	0.671199
2	26	0	-3.785231	-0.521659	-0.492395
3	6	0	1.561433	-0.853726	-2.263850
4	16	0	2.960078	-0.039574	-2.981175
5	6	0	0.931528	-0.067583	-1.170909
6	6	0	3.815817	-1.402374	-3.862394
7	6	0	-0.498272	0.297700	-1.225816
8	6	0	5.023060	-1.871140	-3.031144
9	6	0	-1.009452	1.124176	-0.091231
10	7	0	5.726518	-2.983125	-3.638563
11	8	0	1.136040	-1.956620	-2.614241
12	6	0	-0.121081	1.433798	1.034334
13	6	0	6.862842	-2.779071	-4.369080
14	8	0	7.288223	-1.655584	-4.629734
15	6	0	1.145565	0.973090	1.051679
16	6	0	7.589118	-4.039440	-4.831633
17	6	0	1.677353	0.244618	-0.081037
18	6	0	9.097599	-3.918926	-4.579025
19	6	0	6.599909	-4.380909	-0.069899
20	6	0	5.935649	-5.756247	0.012886
21	6	0	4.403404	-5.656752	-0.048271
22	6	0	3.903396	-5.053044	-1.359359
23	8	0	4.415737	-5.353015	-2.443857
24	7	0	2.847351	-4.210577	-1.255693
25	6	0	4.824840	-0.051098	3.161018
26	6	0	3.461767	-0.214706	3.813126
27	8	0	2.682335	0.745342	3.909286
28	6	0	4.776996	-0.064867	1.613963
29	8	0	4.466812	-1.361935	1.116354
30	6	0	6.135642	0.313021	1.033103
31	7	0	3.119332	-1.456752	4.238317
32	6	0	1.756139	-1.782905	4.682681
33	6	0	1.426899	-3.266560	4.507133
34	6	0	1.775755	-3.842504	3.114162
35	6	0	1.156582	-3.154846	1.879388
36	8	0	-0.107760	-3.291250	1.719442
37	8	0	1.913625	-2.561068	1.082290
38	6	0	0.387993	3.396763	5.693007
39	6	0	-0.108313	2.534842	4.531271
40	8	0	-0.027708	1.138373	4.794199
41	6	0	-3.089824	-3.103381	4.747590
42	6	0	-2.798029	-1.605078	4.956305
43	6	0	-3.459221	-0.640302	3.959146
44	6	0	-3.036635	-0.807197	2.505591
45	8	0	-2.118812	-1.659242	2.281715

46	8	0	-3.614442	-0.103174	1.623190
47	6	0	-1.061897	-7.309027	3.420391
48	6	0	-1.089807	-6.051870	2.543385
49	6	0	-2.266435	-5.951803	1.618547
50	7	0	-2.585603	-4.789418	0.917648
51	6	0	-3.149080	-6.931892	1.242164
52	6	0	-3.617121	-5.075910	0.133329
53	7	0	-3.990285	-6.360251	0.303148
54	6	0	6.000726	6.249682	3.693871
55	6	0	6.219044	4.746417	3.452372
56	6	0	5.727053	4.332412	2.080521
57	6	0	4.402524	3.913964	1.888137
58	6	0	6.562380	4.432543	0.958281
59	6	0	3.921476	3.622222	0.609526
60	6	0	6.088284	4.133497	-0.320218
61	6	0	4.762811	3.732417	-0.499989
62	6	0	-2.873658	8.923421	-1.273296
63	6	0	-2.529774	8.985975	0.229518
64	6	0	-1.569941	7.931423	0.739613
65	6	0	-0.253649	8.266683	1.084146
66	6	0	-1.984781	6.603173	0.920141
67	6	0	0.619225	7.311017	1.608044
68	6	0	-1.117877	5.640672	1.441945
69	6	0	0.187816	5.995837	1.791196
70	6	0	0.026576	5.990063	-3.892084
71	6	0	-0.404749	4.513979	-3.936091
72	8	0	0.277746	3.659993	-4.495323
73	6	0	0.322423	6.568091	-2.496334
74	8	0	0.431448	7.991767	-2.568770
75	6	0	1.568612	5.943268	-1.870972
76	6	0	-8.721152	1.092685	-2.034758
77	6	0	-7.276820	0.749088	-1.615611
78	6	0	-7.162405	-0.335875	-0.586785
79	7	0	-5.946112	-0.879019	-0.173711
80	6	0	-8.168135	-0.967748	0.099480
81	6	0	-6.223649	-1.806364	0.730594
82	7	0	-7.555837	-1.891923	0.927458
83	7	0	-1.631875	4.195511	-3.396426
84	6	0	-2.062592	2.798478	-3.427745
85	6	0	-3.509582	2.543554	-2.953725
86	6	0	-3.663558	2.547894	-1.432007
87	8	0	-3.454680	3.601935	-0.814253
88	8	0	-3.971423	1.424511	-0.844180
89	6	0	-6.538127	-3.150166	-3.566054
90	6	0	-5.345468	-3.141283	-4.540290
91	6	0	-4.485149	-1.868474	-4.457866
92	6	0	-3.609597	-1.843461	-3.200894
93	8	0	-2.851713	-2.805998	-2.986774
94	8	0	-3.716389	-0.783226	-2.463922
95	8	0	-3.022091	2.808725	1.898577
96	8	0	-0.988205	-3.069569	-1.185394
97	8	0	-1.415933	-0.286348	-0.245345
98	8	0	-3.337375	-2.236326	-0.067871
99	1	0	6.214071	-6.263404	0.944875

100	1	0	6.277036	-6.389412	-0.812601
101	1	0	3.959443	-6.658361	0.026839
102	1	0	4.023105	-5.074780	0.799738
103	1	0	2.568629	-3.780830	-0.377664
104	1	0	2.481457	-3.788463	-2.099129
105	1	0	6.450284	-3.945248	-1.062503
106	1	0	4.041609	0.678915	1.281727
107	1	0	6.093706	0.267643	-0.057874
108	1	0	6.904557	-0.389706	1.372432
109	1	0	6.420465	1.326345	1.327356
110	1	0	3.548809	-1.638494	1.305985
111	1	0	5.240307	0.898383	3.507832
112	1	0	0.355866	-3.388160	4.699008
113	1	0	1.952064	-3.869070	5.259808
114	1	0	1.460517	-4.891129	3.101032
115	1	0	2.859092	-3.836040	2.964342
116	1	0	1.075218	-1.196934	4.055470
117	1	0	-1.153030	2.764060	4.294321
118	1	0	0.480857	2.787260	3.636535
119	1	0	0.906119	0.900328	4.652782
120	1	0	0.581878	4.418584	5.342950
121	1	0	-3.132481	-1.313891	5.959691
122	1	0	-1.718300	-1.428224	4.932790
123	1	0	-4.554612	-0.717784	3.983970
124	1	0	-3.228015	0.395667	4.235525
125	1	0	-2.681533	-3.459612	3.801569
126	1	0	-1.049934	-5.151788	3.161048
127	1	0	-0.185326	-6.014853	1.924900
128	1	0	-3.247668	-7.961021	1.545992
129	1	0	-4.733570	-6.826027	-0.194770
130	1	0	-4.073279	-4.378001	-0.552215
131	1	0	-1.197054	-8.218962	2.824732
132	1	0	5.693242	4.172203	4.224527
133	1	0	7.285358	4.510351	3.553450
134	1	0	7.594286	4.751278	1.089159
135	1	0	3.744169	3.809292	2.747584
136	1	0	6.753589	4.212887	-1.175551
137	1	0	2.889525	3.308487	0.481307
138	1	0	4.389643	3.502573	-1.493637
139	1	0	4.938152	6.501135	3.610559
140	1	0	-3.465213	8.916997	0.800801
141	1	0	-2.113326	9.976466	0.448801
142	1	0	0.086556	9.290523	0.948727
143	1	0	-3.002101	6.315745	0.664814
144	1	0	1.633041	7.595680	1.877909
145	1	0	-1.474289	4.626762	1.595001
146	1	0	0.864298	5.252790	2.205357
147	1	0	-1.979794	9.033459	-1.893887
148	1	0	-0.532106	6.401363	-1.831607
149	1	0	2.450442	6.140018	-2.494789
150	1	0	1.466084	4.856768	-1.786266
151	1	0	1.740500	6.355768	-0.875236
152	1	0	1.229537	8.196439	-3.075457
153	1	0	0.920203	6.055577	-4.520154

154	1	0	-6.696020	0.449761	-2.496238
155	1	0	-6.776576	1.645307	-1.229748
156	1	0	-9.238225	-0.843914	0.071194
157	1	0	-8.017574	-2.510843	1.576133
158	1	0	-5.488102	-2.415321	1.231935
159	1	0	-9.316835	1.447672	-1.187767
160	1	0	-3.824623	1.571670	-3.338537
161	1	0	-4.167360	3.315401	-3.370585
162	1	0	-1.363938	2.188763	-2.847097
163	1	0	3.082072	-2.194939	-4.028997
164	1	0	4.138547	-1.010804	-4.829608
165	1	0	-0.963065	0.359340	-2.204137
166	1	0	4.693689	-2.155197	-2.027507
167	1	0	5.733260	-1.046055	-2.937384
168	1	0	-1.839711	1.797565	-0.227451
169	1	0	-0.537480	2.061508	1.812935
170	1	0	1.795111	1.177224	1.895815
171	1	0	7.174283	-4.929568	-4.346203
172	1	0	7.405153	-4.145299	-5.908481
173	1	0	2.711945	-0.072489	-0.061043
174	1	0	9.636806	-4.767812	-5.010555
175	1	0	9.315236	-3.888678	-3.506341
176	1	0	9.473693	-2.995216	-5.024200
177	1	0	-6.263786	-2.707177	-2.601655
178	1	0	-5.710311	-3.266497	-5.566225
179	1	0	-4.688076	-3.992947	-4.336810
180	1	0	-5.102559	-0.966121	-4.491104
181	1	0	-3.801085	-1.836928	-5.314703
182	1	0	-3.287247	3.191738	1.039425
183	1	0	-3.353954	1.899421	1.844330
184	1	0	7.675873	-4.397747	0.105016
185	1	0	6.162554	-3.681604	0.653297
186	1	0	5.507053	-0.863062	3.413013
187	1	0	1.609492	-1.406966	5.694947
188	1	0	-0.092839	-7.403410	3.910540
189	1	0	-1.841751	-7.289184	4.188012
190	1	0	-2.648806	-3.682911	5.558782
191	1	0	-4.169679	-3.303142	4.755833
192	1	0	-6.848615	-4.176217	-3.368098
193	1	0	-7.393309	-2.589540	-3.956188
194	1	0	-9.229582	0.234473	-2.485442
195	1	0	-8.671306	1.893522	-2.772512
196	1	0	-1.982024	2.434220	-4.452030
197	1	0	-3.362278	7.978498	-1.533410
198	1	0	-3.568259	9.738169	-1.479028
199	1	0	-0.767703	6.563447	-4.369992
200	1	0	6.534014	6.841791	2.942521
201	1	0	6.342178	6.574050	4.676976
202	1	0	1.328004	2.997432	6.088141
203	1	0	-0.330634	3.481527	6.508255
204	1	0	3.795937	-2.197967	4.135348
205	1	0	5.391142	-3.921336	-3.429586
206	1	0	-2.068602	4.799145	-2.711205
207	1	0	-1.712347	-2.915078	-1.874736

208	1	0	-0.149167	-2.715973	-1.553566

TS2

E(B3LYP*/LB)= -5205.914739 Number of imaginary frequencies: 9

Center Number	Atomic Number	Atomic Type	Coordinates (Angstroms) X	Y	Z
1	26	0	-1.259052	-2.844688	0.646923
2	26	0	-3.853743	-1.110606	-0.582965
3	6	0	2.099457	-0.201063	-2.231509
4	16	0	3.134510	0.951826	-3.104961
5	6	0	1.036618	0.368839	-1.361434
6	6	0	4.277926	-0.217888	-3.940733
7	6	0	0.431722	1.624199	-1.570441
8	6	0	5.404165	-0.677327	-2.997980
9	6	0	-0.242384	2.303821	-0.558398
10	7	0	6.255334	-1.689915	-3.591970
11	8	0	2.238325	-1.422709	-2.323107
12	6	0	-0.366730	1.827370	0.758066
13	6	0	7.431249	-1.358934	-4.203750
14	8	0	7.788013	-0.194230	-4.369859
15	6	0	-0.267930	0.500008	1.125969
16	6	0	8.277015	-2.543535	-4.670133
17	6	0	0.705922	-0.448938	-0.283958
18	6	0	9.760038	-2.338148	-4.336475
19	6	0	7.216143	-3.325004	0.061180
20	6	0	6.935450	-4.815742	-0.159748
21	6	0	5.451480	-5.127126	-0.405700
22	6	0	4.925019	-4.399225	-1.637972
23	8	0	5.559242	-4.392776	-2.701396
24	7	0	3.733570	-3.779823	-1.491092
25	6	0	4.637626	0.566222	3.308856
26	6	0	3.350986	0.216892	4.069097
27	8	0	2.531440	1.078247	4.415090
28	6	0	4.480734	0.215253	1.802037
29	8	0	4.588865	-1.216847	1.723837
30	6	0	5.587691	0.852152	0.967992
31	7	0	3.148713	-1.116631	4.276288
32	6	0	1.845597	-1.686446	4.673268
33	6	0	1.823527	-3.208001	4.507442
34	6	0	2.324937	-3.725781	3.139443
35	6	0	1.655741	-3.172355	1.871314
36	8	0	0.376119	-3.188755	1.824322
37	8	0	2.386886	-2.767192	0.937582
38	6	0	-0.394113	3.168979	5.736014
39	6	0	-0.643201	2.272318	4.521083
40	8	0	-0.301662	0.905129	4.733696
41	6	0	-2.714724	-3.786635	4.521537
42	6	0	-2.790626	-2.288126	4.870046
43	6	0	-3.602900	-1.417711	3.903401
44	6	0	-3.082774	-1.381283	2.473140
45	8	0	-1.959811	-1.956516	2.272887

46	8	0	-3.777658	-0.797983	1.595246
47	6	0	0.025562	-7.564791	3.188169
48	6	0	-0.181645	-6.235299	2.430265
49	6	0	-1.338014	-6.191713	1.471268
50	7	0	-1.763419	-5.031069	0.812123
51	6	0	-2.097980	-7.240514	1.022103
52	6	0	-2.735030	-5.395982	-0.014966
53	7	0	-2.969767	-6.720474	0.082348
54	6	0	4.736899	6.958028	4.016999
55	6	0	5.142650	5.489933	3.797803
56	6	0	4.712085	4.983786	2.436180
57	6	0	3.579367	4.170583	2.290284
58	6	0	5.408725	5.368710	1.280545
59	6	0	3.143983	3.771788	1.023282
60	6	0	4.979978	4.968245	0.014853
61	6	0	3.839325	4.172519	-0.119111
62	6	0	-4.277208	8.269062	-1.236989
63	6	0	-3.971809	8.388262	0.271650
64	6	0	-2.860316	7.508994	0.806696
65	6	0	-1.591219	8.041920	1.073270
66	6	0	-3.083906	6.154312	1.093844
67	6	0	-0.580469	7.251896	1.623675
68	6	0	-2.078631	5.356077	1.646067
69	6	0	-0.822695	5.907363	1.914599
70	6	0	-0.841960	5.923412	-3.800369
71	6	0	-0.999479	4.397491	-3.837697
72	8	0	-0.112867	3.672036	-4.290294
73	6	0	-0.669143	6.550698	-2.402038
74	8	0	-0.873186	7.963657	-2.472841
75	6	0	0.691249	6.216212	-1.792118
76	6	0	-8.720068	-0.392885	-2.387649
77	6	0	-7.287153	-0.473377	-1.833725
78	6	0	-7.105352	-1.487750	-0.744936
79	7	0	-5.864224	-1.783585	-0.186303
80	6	0	-8.051610	-2.243127	-0.100457
81	6	0	-6.065379	-2.684029	0.764152
82	7	0	-7.375440	-2.992799	0.846086
83	7	0	-2.188893	3.868810	-3.404627
84	6	0	-2.390035	2.420529	-3.483893
85	6	0	-3.748252	1.925991	-2.965772
86	6	0	-3.793610	1.932059	-1.436183
87	8	0	-3.771310	3.006126	-0.833376
88	8	0	-3.839083	0.775213	-0.816543
89	6	0	-5.813429	-4.176891	-3.919687
90	6	0	-4.540545	-3.939073	-4.750171
91	6	0	-4.004857	-2.492791	-4.693299
92	6	0	-3.341346	-2.193951	-3.346554
93	8	0	-2.216050	-2.664412	-3.121857
94	8	0	-4.047005	-1.463260	-2.534299
95	8	0	-3.504968	2.269220	1.966643
96	8	0	-0.341198	-3.031164	-1.239941
97	8	0	-0.608694	-0.494018	0.185817
98	8	0	-2.784923	-2.526245	-0.251768
99	1	0	7.274609	-5.396414	0.707047

100	1	0	7.505801	-5.169957	-1.024844
101	1	0	5.321544	-6.203025	-0.579508
102	1	0	4.849917	-4.865297	0.472841
103	1	0	3.324969	-3.612064	-0.577480
104	1	0	3.390979	-3.193463	-2.241028
105	1	0	6.983571	-2.749955	-0.840480
106	1	0	3.501165	0.543483	1.429944
107	1	0	5.501425	0.538339	-0.075970
108	1	0	6.567363	0.529682	1.336827
109	1	0	5.534603	1.943344	1.006513
110	1	0	3.817785	-1.619393	1.284753
111	1	0	4.903100	1.607619	3.483231
112	1	0	0.792106	-3.537615	4.667193
113	1	0	2.436313	-3.682922	5.283449
114	1	0	2.183220	-4.812988	3.123031
115	1	0	3.399926	-3.565030	3.023887
116	1	0	1.061990	-1.252777	4.038970
117	1	0	-1.692111	2.300677	4.209029
118	1	0	-0.051801	2.675617	3.683982
119	1	0	0.674457	0.878914	4.778654
120	1	0	-0.372969	4.217587	5.412477
121	1	0	-3.234620	-2.175623	5.866936
122	1	0	-1.780114	-1.872348	4.934573
123	1	0	-4.653628	-1.734380	3.854314
124	1	0	-3.625493	-0.378674	4.255010
125	1	0	-2.161374	-3.956404	3.598975
126	1	0	-0.280509	-5.410341	3.141235
127	1	0	0.723535	-6.014079	1.852291
128	1	0	-2.087376	-8.287838	1.275887
129	1	0	-3.641721	-7.239580	-0.461971
130	1	0	-3.250460	-4.722388	-0.682735
131	1	0	0.079995	-8.417648	2.503305
132	1	0	4.693542	4.866018	4.578858
133	1	0	6.230627	5.394568	3.906042
134	1	0	6.297108	5.989555	1.375692
135	1	0	3.043941	3.827565	3.172812
136	1	0	5.537293	5.273455	-0.866616
137	1	0	2.263892	3.142559	0.929515
138	1	0	3.505101	3.855050	-1.102474
139	1	0	3.657230	7.081018	3.880952
140	1	0	-4.892416	8.169196	0.828300
141	1	0	-3.726991	9.434645	0.490834
142	1	0	-1.398893	9.089703	0.856152
143	1	0	-4.061221	5.717845	0.902025
144	1	0	0.392429	7.688311	1.834805
145	1	0	-2.291779	4.318538	1.887613
146	1	0	-0.036971	5.297467	2.352921
147	1	0	-3.402991	8.519919	-1.844348
148	1	0	-1.462878	6.200090	-1.733030
149	1	0	1.497700	6.622338	-2.416860
150	1	0	0.848500	5.135307	-1.728081
151	1	0	0.773770	6.639752	-0.789668
152	1	0	-0.130345	8.340995	-2.964169
153	1	0	0.041678	6.139727	-4.409062

154	1	0	-6.572620	-0.697515	-2.633742
155	1	0	-6.990998	0.507084	-1.438421
156	1	0	-9.120095	-2.306722	-0.223919
157	1	0	-7.788344	-3.638725	1.501756
158	1	0	-5.300344	-3.111972	1.391882
159	1	0	-9.445876	-0.183621	-1.594748
160	1	0	-3.909799	0.909677	-3.330097
161	1	0	-4.559898	2.563308	-3.336945
162	1	0	-1.609163	1.911215	-2.911756
163	1	0	3.684075	-1.061955	-4.297625
164	1	0	4.687465	0.322279	-4.797400
165	1	0	0.595709	2.139707	-2.513936
166	1	0	4.966994	-1.083630	-2.082017
167	1	0	6.028614	0.181497	-2.739092
168	1	0	-0.553765	3.325481	-0.750710
169	1	0	-0.479674	2.543855	1.564492
170	1	0	-0.200871	0.159838	2.154789
171	1	0	7.898960	-3.477924	-4.241845
172	1	0	8.154244	-2.617109	-5.758724
173	1	0	1.342920	-1.256913	0.061643
174	1	0	10.374042	-3.127855	-4.780254
175	1	0	9.924549	-2.348977	-3.253974
176	1	0	10.096228	-1.370721	-4.716730
177	1	0	-5.738554	-3.697885	-2.936905
178	1	0	-4.734828	-4.204680	-5.795862
179	1	0	-3.738507	-4.604263	-4.409071
180	1	0	-4.811109	-1.776340	-4.878393
181	1	0	-3.239999	-2.363740	-5.465501
182	1	0	-3.629099	2.611240	1.061956
183	1	0	-3.603495	1.314576	1.842192
184	1	0	8.273264	-3.169326	0.277506
185	1	0	6.626078	-2.905792	0.883369
186	1	0	5.435369	-0.129070	3.570423
187	1	0	1.603520	-1.367028	5.686675
188	1	0	0.979079	-7.511698	3.713791
189	1	0	-0.770515	-7.753371	3.914475
190	1	0	-2.212874	-4.307336	5.337234
191	1	0	-3.715899	-4.226391	4.418455
192	1	0	-5.957184	-5.244856	-3.755169
193	1	0	-6.702700	-3.775730	-4.416467
194	1	0	-9.014169	-1.319467	-2.890611
195	1	0	-8.777369	0.424666	-3.106256
196	1	0	-2.214544	2.102150	-4.511566
197	1	0	-4.605680	7.257852	-1.500040
198	1	0	-5.089209	8.963784	-1.452860
199	1	0	-1.702752	6.371045	-4.297015
200	1	0	5.234204	7.614521	3.295412
201	1	0	4.985212	7.307604	5.019177
202	1	0	0.586035	2.945893	6.171307
203	1	0	-1.145071	3.112634	6.524143
204	1	0	3.779675	-1.706317	3.747150
205	1	0	6.013680	-2.669765	-3.441112
206	1	0	-2.810881	4.403000	-2.812028
207	1	0	-1.037015	-2.813918	-1.925841

208	1	0	0.509936	-2.647113	-1.516429

Prod$_{oxepin}$

E(B3LYP*/LB)= -5205.920586 Number of imaginary frequencies: 10

Center Number	Atomic Number	Atomic Type	Coordinates (Angstroms) X	Y	Z
1	26	0	-1.165706	-2.849652	0.650510
2	26	0	-3.812832	-1.174677	-0.611630
3	6	0	2.231276	-0.142398	-2.347222
4	16	0	3.219343	1.123340	-3.115184
5	6	0	1.130086	0.314814	-1.443769
6	6	0	4.446707	0.047708	-3.956750
7	6	0	0.477375	1.601082	-1.599908
8	6	0	5.514201	-0.466750	-2.975273
9	6	0	-0.178909	2.241283	-0.595132
10	7	0	6.375856	-1.472293	-3.564070
11	8	0	2.430119	-1.340350	-2.538304
12	6	0	-0.320709	1.777051	0.770516
13	6	0	7.526178	-1.124513	-4.209735
14	8	0	7.848092	0.046539	-4.404333
15	6	0	-0.316210	0.504857	1.196717
16	6	0	8.398056	-2.293646	-4.661153
17	6	0	0.845661	-0.588681	-0.478453
18	6	0	9.854570	-2.087762	-4.227887
19	6	0	7.285231	-3.155890	0.135856
20	6	0	7.045049	-4.648059	-0.119376
21	6	0	5.574859	-4.986847	-0.405866
22	6	0	5.064427	-4.252289	-1.641019
23	8	0	5.734839	-4.196075	-2.680120
24	7	0	3.844767	-3.682082	-1.524392
25	6	0	4.580596	0.657756	3.372960
26	6	0	3.294849	0.279552	4.124836
27	8	0	2.458154	1.121998	4.476797
28	6	0	4.441053	0.294846	1.867200
29	8	0	4.584600	-1.135911	1.804241
30	6	0	5.535847	0.954390	1.034714
31	7	0	3.119112	-1.060176	4.315630
32	6	0	1.826658	-1.666091	4.694746
33	6	0	1.851022	-3.187246	4.523203
34	6	0	2.397491	-3.689580	3.166456
35	6	0	1.736068	-3.173840	1.878238
36	8	0	0.457953	-3.220228	1.815257
37	8	0	2.473903	-2.781375	0.944343
38	6	0	-0.537251	3.130514	5.755595
39	6	0	-0.767331	2.247855	4.527004
40	8	0	-0.395082	0.885658	4.718879
41	6	0	-2.681546	-3.870825	4.481226
42	6	0	-2.779535	-2.376029	4.840899
43	6	0	-3.592465	-1.506101	3.875628
44	6	0	-3.055657	-1.441984	2.452938
45	8	0	-1.905092	-1.967426	2.262813

46	8	0	-3.764289	-0.883342	1.571142
47	6	0	0.160723	-7.577457	3.160664
48	6	0	-0.072868	-6.240648	2.424400
49	6	0	-1.230494	-6.201828	1.466746
50	7	0	-1.672201	-5.040010	0.820687
51	6	0	-1.977679	-7.255626	1.007923
52	6	0	-2.641815	-5.408312	-0.006605
53	7	0	-2.861429	-6.736348	0.078951
54	6	0	4.523094	7.046442	4.113156
55	6	0	4.992217	5.599770	3.879665
56	6	0	4.569269	5.084810	2.519044
57	6	0	3.478420	4.214857	2.382371
58	6	0	5.226758	5.515990	1.356865
59	6	0	3.046298	3.804295	1.118139
60	6	0	4.800178	5.104880	0.093835
61	6	0	3.701395	4.250827	-0.030883
62	6	0	-4.459664	8.174596	-1.236112
63	6	0	-4.214375	8.226346	0.286485
64	6	0	-3.105642	7.337642	0.811701
65	6	0	-1.850009	7.871065	1.134934
66	6	0	-3.319051	5.967965	1.025788
67	6	0	-0.843287	7.065505	1.669917
68	6	0	-2.318049	5.153723	1.562107
69	6	0	-1.076173	5.705545	1.888600
70	6	0	-0.942234	5.922490	-3.771783
71	6	0	-1.060361	4.393715	-3.809832
72	8	0	-0.143933	3.689728	-4.236519
73	6	0	-0.796680	6.546478	-2.368238
74	8	0	-1.020456	7.956311	-2.434962
75	6	0	0.559847	6.229307	-1.741073
76	6	0	-8.686489	-0.582209	-2.478962
77	6	0	-7.255825	-0.626977	-1.913982
78	6	0	-7.061125	-1.617566	-0.805703
79	7	0	-5.821313	-1.869457	-0.221552
80	6	0	-7.998508	-2.382896	-0.160508
81	6	0	-6.017159	-2.753039	0.746322
82	7	0	-7.319809	-3.094073	0.813241
83	7	0	-2.242725	3.834895	-3.398818
84	6	0	-2.411786	2.383026	-3.489834
85	6	0	-3.767930	1.857810	-2.995752
86	6	0	-3.851227	1.858327	-1.467862
87	8	0	-4.006290	2.918201	-0.862948
88	8	0	-3.728997	0.703635	-0.848556
89	6	0	-5.675559	-4.289809	-3.996325
90	6	0	-4.414772	-4.003286	-4.827896
91	6	0	-3.925412	-2.542609	-4.736930
92	6	0	-3.291219	-2.257609	-3.373843
93	8	0	-2.165229	-2.722331	-3.138827
94	8	0	-4.020110	-1.551940	-2.560580
95	8	0	-4.017725	2.160519	1.973794
96	8	0	-0.306350	-3.132873	-1.274375
97	8	0	-0.324053	-0.567701	0.276233
98	8	0	-2.679404	-2.533172	-0.271466
99	1	0	7.378164	-5.237376	0.743892

100	1	0	7.642772	-4.971424	-0.977977
101	1	0	5.470579	-6.062770	-0.596400
102	1	0	4.946236	-4.748030	0.460124
103	1	0	3.401276	-3.556229	-0.620519
104	1	0	3.512334	-3.088413	-2.273693
105	1	0	7.049365	-2.568421	-0.757120
106	1	0	3.455287	0.593543	1.486612
107	1	0	5.464389	0.631549	-0.007647
108	1	0	6.521662	0.661288	1.411443
109	1	0	5.453033	2.043985	1.064781
110	1	0	3.849752	-1.559231	1.324643
111	1	0	4.822007	1.705183	3.544470
112	1	0	0.826223	-3.546595	4.659452
113	1	0	2.460175	-3.646796	5.311159
114	1	0	2.293860	-4.781487	3.151469
115	1	0	3.468842	-3.494439	3.071992
116	1	0	1.039375	-1.252399	4.051326
117	1	0	-1.819055	2.255823	4.221189
118	1	0	-0.188846	2.676262	3.693636
119	1	0	0.580970	0.880299	4.776716
120	1	0	-0.527235	4.183668	5.447133
121	1	0	-3.233446	-2.278083	5.834804
122	1	0	-1.774481	-1.949560	4.918268
123	1	0	-4.638509	-1.834620	3.810653
124	1	0	-3.633111	-0.471743	4.239603
125	1	0	-2.120300	-4.028100	3.561042
126	1	0	-0.187052	-5.430529	3.149952
127	1	0	0.827869	-5.992007	1.851063
128	1	0	-1.952238	-8.305384	1.250453
129	1	0	-3.529243	-7.257996	-0.467993
130	1	0	-3.165006	-4.734644	-0.667942
131	1	0	0.244205	-8.416940	2.462381
132	1	0	4.578843	4.950969	4.660127
133	1	0	6.084383	5.552125	3.976584
134	1	0	6.082871	6.181631	1.444433
135	1	0	2.973467	3.837353	3.268773
136	1	0	5.326998	5.447065	-0.792784
137	1	0	2.201902	3.127157	1.032204
138	1	0	3.369027	3.924023	-1.011696
139	1	0	3.439420	7.124005	3.974922
140	1	0	-5.149398	7.961377	0.796914
141	1	0	-3.997879	9.264116	0.567522
142	1	0	-1.664838	8.930305	0.974202
143	1	0	-4.285442	5.529986	0.788404
144	1	0	0.118849	7.501285	1.926666
145	1	0	-2.529925	4.103958	1.746798
146	1	0	-0.293379	5.085526	2.318163
147	1	0	-3.570977	8.477986	-1.796932
148	1	0	-1.593654	6.179922	-1.711935
149	1	0	1.369054	6.655198	-2.348806
150	1	0	0.734595	5.150868	-1.684400
151	1	0	0.618777	6.645113	-0.733765
152	1	0	-0.275629	8.346830	-2.912787
153	1	0	-0.057727	6.161020	-4.370764

154	1	0	-6.532608	-0.849560	-2.706365
155	1	0	-6.983504	0.366100	-1.532662
156	1	0	-9.063072	-2.474468	-0.298802
157	1	0	-7.727563	-3.732802	1.479110
158	1	0	-5.254492	-3.146418	1.398728
159	1	0	-9.423476	-0.391350	-1.691806
160	1	0	-3.893916	0.837055	-3.362484
161	1	0	-4.587149	2.474250	-3.383740
162	1	0	-1.629845	1.889486	-2.906544
163	1	0	3.898335	-0.778486	-4.413286
164	1	0	4.903040	0.658106	-4.738930
165	1	0	0.575014	2.106071	-2.558094
166	1	0	5.026589	-0.905387	-2.101659
167	1	0	6.133396	0.373056	-2.648722
168	1	0	-0.577733	3.231293	-0.798313
169	1	0	-0.439358	2.532436	1.540786
170	1	0	-0.374116	0.200888	2.236767
171	1	0	8.003203	-3.241521	-4.280777
172	1	0	8.344494	-2.334012	-5.756533
173	1	0	1.490144	-1.430285	-0.249557
174	1	0	10.501605	-2.866458	-4.643543
175	1	0	9.948169	-2.117098	-3.137202
176	1	0	10.209985	-1.112794	-4.569847
177	1	0	-5.610490	-3.818703	-3.009011
178	1	0	-4.605636	-4.251158	-5.878464
179	1	0	-3.591997	-4.650802	-4.503016
180	1	0	-4.751008	-1.847376	-4.917291
181	1	0	-3.154481	-2.373498	-5.495291
182	1	0	-4.070218	2.512115	1.066869
183	1	0	-3.870468	1.216445	1.819827
184	1	0	8.335951	-2.976809	0.364886
185	1	0	6.675764	-2.768049	0.959223
186	1	0	5.391330	-0.020148	3.640167
187	1	0	1.565872	-1.357563	5.706884
188	1	0	1.106797	-7.504919	3.697296
189	1	0	-0.636864	-7.798788	3.876072
190	1	0	-2.176881	-4.383891	5.300018
191	1	0	-3.675397	-4.324774	4.370671
192	1	0	-5.796247	-5.361651	-3.838623
193	1	0	-6.577457	-3.905841	-4.484184
194	1	0	-8.953795	-1.514158	-2.986584
195	1	0	-8.754753	0.237452	-3.194200
196	1	0	-2.217429	2.074063	-4.516985
197	1	0	-4.743906	7.166930	-1.558226
198	1	0	-5.285149	8.851366	-1.457791
199	1	0	-1.807596	6.352542	-4.275965
200	1	0	4.993145	7.731345	3.399817
201	1	0	4.751958	7.396566	5.119764
202	1	0	0.442143	2.913332	6.195285
203	1	0	-1.295477	3.052717	6.534890
204	1	0	3.760406	-1.625008	3.771162
205	1	0	6.150203	-2.454662	-3.405814
206	1	0	-2.915593	4.368006	-2.864560
207	1	0	-1.019258	-2.907339	-1.942510

208	1	0	0.538944	-2.787331	-1.599767

TS3

E(B3LYP*/LB)= -5205.906827 Number of imaginary frequencies: 6

Center Number	Atomic Number	Atomic Type	Coordinates (Angstroms) X	Y	Z
1	26	0	-1.779277	-2.700637	0.560220
2	26	0	-3.693143	-0.563994	-0.566803
3	6	0	2.136469	-0.325295	-2.444159
4	16	0	3.310049	0.711706	-3.310225
5	6	0	1.203529	0.352679	-1.488674
6	6	0	4.332822	-0.616952	-4.066448
7	6	0	0.888663	1.709247	-1.512991
8	6	0	5.324028	-1.225814	-3.057546
9	6	0	-0.029933	2.295786	-0.581538
10	7	0	5.999628	-2.396031	-3.583116
11	8	0	2.120069	-1.539109	-2.569646
12	6	0	-0.660806	1.535412	0.352278
13	6	0	7.230828	-2.306357	-4.169017
14	8	0	7.798809	-1.232488	-4.357442
15	6	0	-0.371866	0.071309	0.459361
16	6	0	7.849717	-3.641786	-4.583457
17	6	0	0.650376	-0.441910	-0.475035
18	6	0	9.355648	-3.683016	-4.297090
19	6	0	6.683445	-4.322025	0.088253
20	6	0	6.136973	-5.723066	-0.194220
21	6	0	4.613144	-5.725624	-0.396034
22	6	0	4.189908	-4.861795	-1.581447
23	8	0	4.799320	-4.910843	-2.657888
24	7	0	3.103552	-4.078651	-1.390629
25	6	0	4.686076	-0.121740	3.360603
26	6	0	3.356961	-0.294990	4.104477
27	8	0	2.655646	0.670739	4.434857
28	6	0	4.499083	-0.394469	1.841654
29	8	0	4.370590	-1.811437	1.683822
30	6	0	5.710403	0.085747	1.047733
31	7	0	2.972068	-1.586098	4.320650
32	6	0	1.593535	-1.955891	4.703200
33	6	0	1.320811	-3.444458	4.483586
34	6	0	1.709541	-3.983373	3.083878
35	6	0	1.102000	-3.286611	1.850711
36	8	0	-0.166422	-3.325508	1.721278
37	8	0	1.881850	-2.761491	1.016084
38	6	0	0.077221	3.164686	5.798023
39	6	0	-0.310736	2.267101	4.617783
40	8	0	-0.166536	0.879242	4.895630
41	6	0	-3.222899	-3.372868	4.528570
42	6	0	-3.082925	-1.877711	4.872853
43	6	0	-3.738741	-0.898374	3.888773
44	6	0	-3.182121	-0.940417	2.472871
45	8	0	-2.218627	-1.747726	2.271167

46	8	0	-3.703555	-0.197212	1.589986
47	6	0	-1.053939	-7.498598	3.171952
48	6	0	-1.077371	-6.203441	2.351015
49	6	0	-2.271279	-6.048190	1.456320
50	7	0	-2.567135	-4.865345	0.779019
51	6	0	-3.208434	-6.990656	1.115560
52	6	0	-3.646974	-5.104665	0.047054
53	7	0	-4.068141	-6.376093	0.222488
54	6	0	5.707407	6.183236	4.115968
55	6	0	5.838122	4.663566	3.910047
56	6	0	5.275146	4.223064	2.574256
57	6	0	4.032137	3.580116	2.492696
58	6	0	5.962010	4.499623	1.382176
59	6	0	3.482382	3.242928	1.252717
60	6	0	5.419161	4.158563	0.143350
61	6	0	4.171269	3.533591	0.073976
62	6	0	-3.007510	8.822124	-1.143869
63	6	0	-2.700085	8.864302	0.367779
64	6	0	-1.721087	7.826067	0.875439
65	6	0	-0.387189	8.166893	1.141083
66	6	0	-2.131322	6.509068	1.129494
67	6	0	0.507337	7.225296	1.653318
68	6	0	-1.243188	5.561493	1.644778
69	6	0	0.081885	5.919551	1.908408
70	6	0	0.059400	6.022193	-3.718673
71	6	0	-0.291907	4.533106	-3.676696
72	8	0	0.550015	3.672110	-3.952996
73	6	0	0.353173	6.666697	-2.344410
74	8	0	0.340608	8.089492	-2.465451
75	6	0	1.668825	6.173233	-1.746497
76	6	0	-8.653772	0.902576	-2.366543
77	6	0	-7.224780	0.639635	-1.847142
78	6	0	-7.123522	-0.426784	-0.799390
79	7	0	-5.911491	-0.908398	-0.309474
80	6	0	-8.142548	-1.088952	-0.162838
81	6	0	-6.203957	-1.832672	0.592072
82	7	0	-7.541540	-1.973571	0.714286
83	7	0	-1.570132	4.181720	-3.348988
84	6	0	-1.979832	2.777822	-3.430271
85	6	0	-3.405921	2.489952	-2.942414
86	6	0	-3.504575	2.509723	-1.412631
87	8	0	-3.388428	3.588145	-0.816149
88	8	0	-3.688083	1.374963	-0.803965
89	6	0	-6.321150	-3.251103	-3.921005
90	6	0	-5.063414	-3.171997	-4.803941
91	6	0	-4.274661	-1.860979	-4.633903
92	6	0	-3.491296	-1.828248	-3.316492
93	8	0	-2.685975	-2.752308	-3.096585
94	8	0	-3.724581	-0.815712	-2.548261
95	8	0	-3.191231	2.733735	1.920203
96	8	0	-0.910958	-3.263512	-1.283150
97	8	0	-1.352680	-0.743729	-0.210963
98	8	0	-3.379846	-2.312824	-0.174408
99	1	0	6.380548	-6.403570	0.630924

100	1	0	6.604693	-6.131181	-1.096275
101	1	0	4.265350	-6.746562	-0.601018
102	1	0	4.104891	-5.389921	0.515950
103	1	0	2.749876	-3.858970	-0.465132
104	1	0	2.834779	-3.433043	-2.123630
105	1	0	6.542193	-3.675287	-0.783771
106	1	0	3.597749	0.116133	1.475084
107	1	0	5.587953	-0.160430	-0.010612
108	1	0	6.616028	-0.415360	1.406379
109	1	0	5.843093	1.167072	1.140703
110	1	0	3.472259	-2.059471	1.384816
111	1	0	5.095985	0.865331	3.570502
112	1	0	0.251193	-3.607397	4.650353
113	1	0	1.856499	-4.045923	5.228470
114	1	0	1.403653	-5.034576	3.041982
115	1	0	2.795231	-3.975907	2.952281
116	1	0	0.898238	-1.379234	4.077832
117	1	0	-1.353484	2.434934	4.327117
118	1	0	0.306734	2.544220	3.747641
119	1	0	0.795056	0.711491	4.895698
120	1	0	0.224558	4.194153	5.447323
121	1	0	-3.524914	-1.696042	5.860546
122	1	0	-2.023756	-1.614045	4.958723
123	1	0	-4.821204	-1.070466	3.807596
124	1	0	-3.631090	0.132446	4.247150
125	1	0	-2.700437	-3.620880	3.606048
126	1	0	-1.019459	-5.332362	3.008016
127	1	0	-0.177641	-6.152214	1.725201
128	1	0	-3.336198	-8.016489	1.420292
129	1	0	-4.859946	-6.802076	-0.234379
130	1	0	-4.116381	-4.376260	-0.597091
131	1	0	-1.147907	-8.383958	2.533058
132	1	0	5.311768	4.139545	4.715652
133	1	0	6.894661	4.376413	3.986435
134	1	0	6.933140	4.987906	1.426846
135	1	0	3.497352	3.318192	3.402232
136	1	0	5.969949	4.375860	-0.767594
137	1	0	2.514884	2.750022	1.214817
138	1	0	3.753273	3.263816	-0.891630
139	1	0	4.669815	6.500859	3.967765
140	1	0	-3.644767	8.757879	0.916618
141	1	0	-2.315609	9.861292	0.614692
142	1	0	-0.050400	9.183492	0.953023
143	1	0	-3.161839	6.219460	0.938644
144	1	0	1.533860	7.514275	1.864215
145	1	0	-1.601997	4.560689	1.866405
146	1	0	0.777504	5.190841	2.315776
147	1	0	-2.101283	8.959291	-1.740967
148	1	0	-0.466593	6.448422	-1.651274
149	1	0	2.508969	6.440732	-2.400808
150	1	0	1.678974	5.084752	-1.640605
151	1	0	1.829235	6.618652	-0.763371
152	1	0	1.122915	8.347662	-2.972405
153	1	0	0.954758	6.089968	-4.345542

154	1	0	-6.569157	0.365032	-2.682040
155	1	0	-6.806091	1.568735	-1.439508
156	1	0	-9.213352	-1.010245	-0.254877
157	1	0	-8.012917	-2.601295	1.347205
158	1	0	-5.477094	-2.410047	1.140211
159	1	0	-9.331033	1.202818	-1.560771
160	1	0	-3.692734	1.501612	-3.306163
161	1	0	-4.104857	3.232162	-3.346691
162	1	0	-1.281191	2.164280	-2.853769
163	1	0	3.646271	-1.372719	-4.451893
164	1	0	4.862351	-0.149514	-4.899601
165	1	0	1.292912	2.349640	-2.295239
166	1	0	4.789118	-1.514841	-2.149118
167	1	0	6.081107	-0.481292	-2.799478
168	1	0	-0.250714	3.354646	-0.660870
169	1	0	-1.401770	1.964616	1.029263
170	1	0	-0.244673	-0.224010	1.509033
171	1	0	7.327242	-4.471427	-4.095293
172	1	0	7.679820	-3.751711	-5.662933
173	1	0	0.938989	-1.481554	-0.365522
174	1	0	9.806979	-4.590704	-4.709283
175	1	0	9.552087	-3.665388	-3.220094
176	1	0	9.842997	-2.811098	-4.739443
177	1	0	-6.132027	-2.821943	-2.929697
178	1	0	-5.345977	-3.292836	-5.856131
179	1	0	-4.383034	-3.996358	-4.566368
180	1	0	-4.934366	-0.990117	-4.691006
181	1	0	-3.535190	-1.775368	-5.439297
182	1	0	-3.357031	3.120226	1.036416
183	1	0	-3.528285	1.827991	1.828146
184	1	0	7.751239	-4.322849	0.308341
185	1	0	6.166036	-3.844026	0.927869
186	1	0	5.373962	-0.927059	3.618299
187	1	0	1.397564	-1.612449	5.718807
188	1	0	-0.104021	-7.587803	3.699186
189	1	0	-1.862457	-7.524965	3.909371
190	1	0	-2.802779	-3.966705	5.340633
191	1	0	-4.277141	-3.662263	4.423063
192	1	0	-6.618374	-4.288198	-3.764809
193	1	0	-7.163518	-2.706173	-4.359099
194	1	0	-9.072877	0.027133	-2.871610
195	1	0	-8.590224	1.725190	-3.078671
196	1	0	-1.850062	2.445053	-4.460484
197	1	0	-3.472045	7.872347	-1.429835
198	1	0	-3.709640	9.628629	-1.356065
199	1	0	-0.726246	6.593116	-4.213272
200	1	0	6.324346	6.734492	3.398916
201	1	0	6.000753	6.486061	5.121206
202	1	0	1.023174	2.826524	6.234102
203	1	0	-0.676217	3.212036	6.584400
204	1	0	3.525274	-2.269744	3.820231
205	1	0	5.575257	-3.308325	-3.414092
206	1	0	-2.209253	4.852052	-2.944594
207	1	0	-1.592314	-2.987762	-1.984696

208	1	0	-0.745114	-4.206976	-1.401108

Prod$_{phenol}$
E(B3LYP*/LB)= -5205.990549   Number of imaginary frequencies: 15

Center Number	Atomic Number	Atomic Type	Coordinates (Angstroms) X	Y	Z
1	26	0	-2.682387	-2.418281	0.268163
2	26	0	-3.909542	0.095882	-0.668288
3	6	0	2.413834	-0.655817	-2.585402
4	16	0	3.784832	0.293816	-3.251154
5	6	0	1.449024	0.051104	-1.700347
6	6	0	4.688816	-1.105009	-4.018012
7	6	0	1.717216	1.292533	-1.103763
8	6	0	5.422508	-1.977882	-2.980917
9	6	0	0.774375	1.854914	-0.242953
10	7	0	5.738013	-3.294253	-3.497086
11	8	0	2.286482	-1.843419	-2.861467
12	6	0	-0.443587	1.218930	0.010395
13	6	0	6.961051	-3.592248	-4.021158
14	8	0	7.869924	-2.769000	-4.112107
15	6	0	-0.740223	-0.001769	-0.633930
16	6	0	7.126289	-5.039482	-4.479768
17	6	0	0.228182	-0.593812	-1.461237
18	6	0	8.420893	-5.644666	-3.928685
19	6	0	5.605459	-5.626804	0.413354
20	6	0	4.943972	-6.797401	-0.319150
21	6	0	3.474924	-6.519656	-0.677584
22	6	0	3.351860	-5.399201	-1.706581
23	8	0	3.877697	-5.501856	-2.818812
24	7	0	2.627079	-4.316982	-1.333074
25	6	0	4.479828	-1.039468	3.580278
26	6	0	3.083625	-0.922066	4.177871
27	8	0	2.491196	0.160860	4.214585
28	6	0	4.379889	-1.045877	2.030887
29	8	0	3.521926	-2.111932	1.590405
30	6	0	5.742965	-1.243763	1.384980
31	7	0	2.486198	-2.083156	4.567387
32	6	0	1.051701	-2.161294	4.891158
33	6	0	0.486716	-3.529760	4.534333
34	6	0	0.735613	-3.905782	3.041731
35	6	0	0.055796	-3.036593	2.000002
36	8	0	-0.830656	-3.434149	1.255464
37	8	0	0.557822	-1.799292	1.926566
38	6	0	0.622614	3.183563	5.876291
39	6	0	0.043932	2.422013	4.679577
40	8	0	-0.152737	1.034701	4.955462
41	6	0	-3.951814	-2.543622	4.644585
42	6	0	-3.088020	-1.277411	4.799403
43	6	0	-3.367010	-0.123854	3.835168
44	6	0	-3.248167	-0.475437	2.364129
45	8	0	-2.637179	-1.569055	2.081545

46	8	0	-3.746180	0.318421	1.522544
47	6	0	-2.672943	-7.056806	3.394778
48	6	0	-2.450689	-5.848544	2.472570
49	6	0	-3.636849	-5.532074	1.603683
50	7	0	-3.738922	-4.389095	0.808192
51	6	0	-4.777541	-6.277742	1.441067
52	6	0	-4.912740	-4.459851	0.192105
53	7	0	-5.568997	-5.586231	0.543452
54	6	0	6.785425	4.926305	4.257361
55	6	0	6.934233	3.416748	3.997431
56	6	0	6.478347	3.024744	2.608894
57	6	0	5.119488	2.807327	2.339886
58	6	0	7.390856	2.926007	1.549326
59	6	0	4.684932	2.513941	1.046431
60	6	0	6.960912	2.624214	0.255578
61	6	0	5.604068	2.419504	-0.001813
62	6	0	-1.100159	9.225713	-1.211507
63	6	0	-0.971510	9.142073	0.323935
64	6	0	-0.246999	7.927610	0.869450
65	6	0	1.084680	8.023589	1.298060
66	6	0	-0.897899	6.691504	1.000220
67	6	0	1.741688	6.926864	1.858191
68	6	0	-0.248851	5.589056	1.562690
69	6	0	1.073570	5.708174	1.998424
70	6	0	1.353079	5.797561	-3.682134
71	6	0	0.650837	4.431673	-3.785345
72	8	0	1.182846	3.481848	-4.353172
73	6	0	1.661877	6.305557	-2.263125
74	8	0	2.040410	7.684477	-2.303127
75	6	0	2.729840	5.456994	-1.574150
76	6	0	-8.258428	2.638293	-2.420502
77	6	0	-6.944970	2.102939	-1.835605
78	6	0	-7.134720	1.116477	-0.728841
79	7	0	-6.090803	0.355511	-0.223485
80	6	0	-8.279301	0.800136	-0.041056
81	6	0	-6.592898	-0.396735	0.740928
82	7	0	-7.914953	-0.159925	0.887263
83	7	0	-0.636385	4.346249	-3.304494
84	6	0	-1.323993	3.057176	-3.383343
85	6	0	-2.797449	3.067991	-2.940819
86	6	0	-2.957076	3.039916	-1.419220
87	8	0	-2.548760	3.995601	-0.748611
88	8	0	-3.521585	1.987266	-0.890645
89	6	0	-6.822401	-1.940081	-3.864516
90	6	0	-5.703819	-2.085785	-4.906837
91	6	0	-4.661105	-0.954283	-4.826930
92	6	0	-3.899379	-1.049533	-3.505433
93	8	0	-3.153278	-2.039490	-3.351059
94	8	0	-4.122865	-0.108547	-2.657810
95	8	0	-2.641578	3.044894	1.949087
96	8	0	-1.909984	-3.212913	-1.499360
97	8	0	-1.940525	-0.599457	-0.489761
98	8	0	-4.200856	-1.742746	-0.326407
99	1	0	4.993856	-7.702968	0.297403

100	1	0	5.485093	-7.012467	-1.246614
101	1	0	3.028633	-7.411950	-1.131250
102	1	0	2.897573	-6.289380	0.226664
103	1	0	2.421295	-4.153995	-0.360434
104	1	0	2.531744	-3.525382	-1.965901
105	1	0	5.597928	-4.720066	-0.200406
106	1	0	3.963568	-0.083456	1.713057
107	1	0	5.649387	-1.217136	0.296491
108	1	0	6.166459	-2.213193	1.669979
109	1	0	6.433707	-0.452197	1.688664
110	1	0	2.610783	-1.791047	1.645441
111	1	0	5.075847	-0.185047	3.908812
112	1	0	-0.585900	-3.542468	4.751864
113	1	0	0.940604	-4.321098	5.143958
114	1	0	0.401338	-4.928557	2.863413
115	1	0	1.810369	-3.853386	2.833167
116	1	0	0.565438	-1.412228	4.259707
117	1	0	-0.923325	2.835675	4.377176
118	1	0	0.717503	2.536813	3.817701
119	1	0	0.726923	0.631630	4.847961
120	1	0	1.022153	4.150395	5.544829
121	1	0	-3.203843	-0.890533	5.818754
122	1	0	-2.032854	-1.553457	4.704542
123	1	0	-4.362275	0.309039	3.989145
124	1	0	-2.644883	0.681412	4.023650
125	1	0	-3.772313	-3.050118	3.695398
126	1	0	-2.209185	-4.960485	3.065155
127	1	0	-1.580263	-6.020580	1.828376
128	1	0	-5.090268	-7.212459	1.876505
129	1	0	-6.477843	-5.866555	0.207532
130	1	0	-5.294167	-3.700600	-0.474950
131	1	0	-2.966533	-7.941610	2.818704
132	1	0	6.352742	2.865651	4.746965
133	1	0	7.982273	3.126235	4.137872
134	1	0	8.449373	3.087241	1.741416
135	1	0	4.395125	2.859989	3.149712
136	1	0	7.685318	2.546334	-0.550446
137	1	0	3.627421	2.350956	0.861991
138	1	0	5.268695	2.177567	-1.006127
139	1	0	5.741701	5.240060	4.155371
140	1	0	-1.978835	9.176831	0.758962
141	1	0	-0.456928	10.043853	0.677615
142	1	0	1.606641	8.972641	1.201787
143	1	0	-1.930248	6.586495	0.675699
144	1	0	2.770057	7.028172	2.195772
145	1	0	-0.784998	4.652923	1.684719
146	1	0	1.577148	4.858322	2.452234
147	1	0	-0.120951	9.210413	-1.698677
148	1	0	0.752356	6.298686	-1.652671
149	1	0	3.670130	5.490105	-2.139914
150	1	0	2.420360	4.408682	-1.517548
151	1	0	2.912431	5.824831	-0.562696
152	1	0	2.874797	7.740862	-2.789180
153	1	0	2.281637	5.695791	-4.252104

154	1	0	-6.339593	1.630680	-2.617498
155	1	0	-6.339074	2.936627	-1.458428
156	1	0	-9.291008	1.161160	-0.125480
157	1	0	-8.520363	-0.592728	1.567327
158	1	0	-6.034064	-1.113684	1.318869
159	1	0	-8.889600	3.094011	-1.650197
160	1	0	-3.288813	2.188662	-3.362345
161	1	0	-3.299826	3.964535	-3.324255
162	1	0	-0.781171	2.313607	-2.783624
163	1	0	3.950690	-1.697983	-4.560207
164	1	0	5.388320	-0.664278	-4.731992
165	1	0	2.650548	1.806620	-1.303564
166	1	0	4.795005	-2.098526	-2.094242
167	1	0	6.357970	-1.496629	-2.686101
168	1	0	0.974006	2.811882	0.230319
169	1	0	-1.173242	1.684344	0.667533
170	1	0	0.073762	-1.263857	1.263572
171	1	0	6.256175	-5.639193	-4.193651
172	1	0	7.166804	-5.026008	-5.576397
173	1	0	0.008218	-1.537263	-1.946407
174	1	0	8.601199	-6.639309	-4.348416
175	1	0	8.377545	-5.741286	-2.838341
176	1	0	9.267622	-4.999047	-4.174041
177	1	0	-6.420616	-1.621675	-2.896586
178	1	0	-6.132393	-2.117755	-5.915407
179	1	0	-5.173909	-3.033145	-4.759584
180	1	0	-5.138776	0.026178	-4.910777
181	1	0	-3.937825	-1.061709	-5.642171
182	1	0	-2.679789	3.471727	1.069928
183	1	0	-3.151794	2.231010	1.816259
184	1	0	6.645852	-5.846294	0.654000
185	1	0	5.084540	-5.393292	1.349758
186	1	0	4.979668	-1.965732	3.863962
187	1	0	0.915001	-1.764334	5.896984
188	1	0	-1.771163	-7.332094	3.941931
189	1	0	-3.463309	-6.857509	4.125723
190	1	0	-3.678097	-3.196168	5.474009
191	1	0	-5.022310	-2.320691	4.731035
192	1	0	-7.332372	-2.889010	-3.697837
193	1	0	-7.566221	-1.196263	-4.169662
194	1	0	-8.833745	1.854210	-2.922228
195	1	0	-8.013103	3.415845	-3.143824
196	1	0	-1.250498	2.684809	-4.405283
197	1	0	-1.691402	8.393051	-1.607328
198	1	0	-1.614763	10.156854	-1.449793
199	1	0	0.712331	6.510623	-4.200948
200	1	0	7.377606	5.506328	3.542228
201	1	0	7.120039	5.180470	5.263205
202	1	0	1.447570	2.619649	6.324655
203	1	0	-0.116507	3.402477	6.647016
204	1	0	3.036247	-2.928718	4.550918
205	1	0	5.027105	-4.021029	-3.429412
206	1	0	-0.959876	4.991517	-2.594459
207	1	0	-2.392730	-2.668451	-2.232964

208	1	0	-2.067653	-4.147770	-1.679907

Epoxide

E(B3LYP*/LB)= -858.0327938

Center Number	Atomic Number	Atomic Type	Coordinates (Angstroms) X	Y	Z
1	6	0	-1.098796	-0.443340	-0.080406
2	16	0	-2.350154	0.856520	0.086794
3	6	0	0.320939	-0.023717	0.009778
4	6	0	-3.832792	-0.197759	-0.038305
5	6	0	0.728750	1.267597	0.167984
6	6	0	2.103677	1.661665	-0.016294
7	8	0	-1.426360	-1.601893	-0.270554
8	6	0	3.058009	0.760324	-0.357229
9	6	0	2.770244	-0.678529	-0.327949
10	6	0	1.325497	-1.095888	-0.130930
11	8	0	2.292959	-1.210587	0.915165
12	1	0	-3.847604	-0.927049	0.772076
13	1	0	-4.691786	0.470783	0.039681
14	1	0	-0.003387	2.052406	0.335820
15	1	0	2.339850	2.721226	0.014960
16	1	0	4.043841	1.087677	-0.675408
17	1	0	3.440416	-1.348327	-0.865317
18	1	0	0.979031	-2.045024	-0.531312
19	1	0	-3.843859	-0.718309	-0.995982

TS_{U1}

E(B3LYP*/LB)= -858.0236521

Center Number	Atomic Number	Atomic Type	Coordinates (Angstroms) X	Y	Z
1	6	0	1.110642	-0.504728	0.013512
2	16	0	2.305707	0.855841	-0.027652
3	6	0	-0.310778	-0.136189	-0.225505
4	6	0	3.828833	-0.130322	0.155249
5	6	0	-0.738596	1.188743	-0.436353
6	6	0	-1.996162	1.644374	-0.039374
7	8	0	1.472192	-1.646249	0.238304
8	6	0	-2.933979	0.813405	0.588116
9	6	0	-2.923950	-0.566352	0.424370
10	6	0	-1.238490	-1.177722	-0.093327
11	8	0	-2.445799	-1.107846	-0.773294
12	1	0	3.986077	-0.757833	-0.722856
13	1	0	4.648061	0.583162	0.258206
14	1	0	-0.009118	1.930236	-0.749233
15	1	0	-2.169308	2.716978	-0.042375
16	1	0	-3.640044	1.239516	1.296674
17	1	0	-3.511070	-1.245646	1.037991
18	1	0	-0.949502	-2.155809	0.280865

19	1	0	3.757319	-0.758557	1.042957

Oxepin

E(B3LYP*/LB)= -858.0330746

Center Number	Atomic Number	Atomic Type	Coordinates (Angstroms) X	Y	Z
1	6	0	1.159249	-0.548268	-0.021883
2	16	0	2.273639	0.877673	0.016456
3	6	0	-0.284161	-0.223534	-0.235753
4	6	0	3.842544	-0.025853	0.232829
5	6	0	-0.727229	1.151865	-0.483670
6	6	0	-1.934780	1.651121	-0.145702
7	8	0	1.589088	-1.677139	0.128611
8	6	0	-2.988591	0.901621	0.528829
9	6	0	-3.182174	-0.416957	0.423116
10	6	0	-1.113550	-1.291057	-0.200516
11	8	0	-2.462617	-1.245642	-0.443303
12	1	0	4.051215	-0.639681	-0.644016
13	1	0	4.620090	0.729532	0.357649
14	1	0	-0.005995	1.819731	-0.946922
15	1	0	-2.127887	2.704469	-0.334622
16	1	0	-3.666198	1.445628	1.182677
17	1	0	-3.947450	-0.958583	0.971774
18	1	0	-0.727033	-2.292397	-0.031399
19	1	0	3.785437	-0.662839	1.115593

TS_{U2}

E(B3LYP*/LB)= -858.0294306

Center Number	Atomic Number	Atomic Type	Coordinates (Angstroms) X	Y	Z
1	6	0	1.183498	-0.519662	0.000014
2	16	0	2.332225	0.886091	-0.000051
3	6	0	-0.268273	-0.174236	0.000009
4	6	0	3.890015	-0.060110	-0.000020
5	6	0	-0.699696	1.240313	-0.000044
6	6	0	-1.958914	1.699397	-0.000054
7	8	0	1.622728	-1.657281	0.000059
8	6	0	-3.190769	0.897926	-0.000016
9	6	0	-3.345158	-0.426941	0.000036
10	6	0	-1.055601	-1.276135	0.000056
11	8	0	-2.398653	-1.447058	0.000070
12	1	0	3.948538	-0.687404	-0.889686
13	1	0	4.696308	0.675376	-0.000054
14	1	0	0.095633	1.978074	-0.000076
15	1	0	-2.105255	2.775707	-0.000095
16	1	0	-4.125438	1.451841	-0.000033
17	1	0	-4.326702	-0.885849	0.000059
18	1	0	-0.570429	-2.246465	0.000092

19	1	0	3.948546	-0.687332	0.889696

TS$_{U3}$
E(B3LYP*/LB)= -857.9647111

Center Number	Atomic Number	Atomic Type	Coordinates (Angstroms) X	Y	Z
1	6	0	1.030576	0.325779	-0.363642
2	16	0	2.184210	-0.782253	0.482151
3	6	0	-0.387470	-0.078065	-0.196267
4	6	0	3.757937	0.038025	0.056230
5	6	0	-0.816286	-1.374423	-0.291174
6	6	0	-2.189359	-1.704242	-0.103434
7	8	0	1.368764	1.321854	-0.968659
8	6	0	-3.177447	-0.735101	0.044031
9	6	0	-2.806682	0.602701	-0.059768
10	6	0	-1.396636	1.059031	-0.052413
11	8	0	-1.331539	1.952310	0.927515
12	1	0	3.508952	0.969303	-0.457998
13	1	0	4.306437	0.247080	0.975181
14	1	0	-0.107037	-2.172162	-0.492731
15	1	0	-2.468052	-2.755103	-0.104983
16	1	0	-4.210615	-1.029418	0.203164
17	1	0	-3.531849	1.406543	-0.001297
18	1	0	-1.177322	1.462748	-1.111706
19	1	0	4.346531	-0.608485	-0.596269

Int1$_{U1}$
E(B3LYP*/LB)= -858.0661065

Center Number	Atomic Number	Atomic Type	Coordinates (Angstroms) X	Y	Z
1	6	0	-1.030667	0.288476	0.662962
2	16	0	-1.611439	-0.797296	-0.656479
3	6	0	0.491751	0.212725	0.872788
4	6	0	-3.376783	-0.332973	-0.695683
5	6	0	1.024264	-1.190782	0.971021
6	6	0	2.140725	-1.572017	0.326818
7	8	0	-1.749115	1.010759	1.309702
8	6	0	2.862974	-0.649165	-0.538339
9	6	0	2.440735	0.616308	-0.760768
10	6	0	1.203928	1.129815	-0.159583
11	8	0	0.747977	2.230319	-0.426319
12	1	0	-3.549825	0.355689	0.134228
13	1	0	-3.604631	0.156196	-1.643527
14	1	0	0.492370	-1.881282	1.619760
15	1	0	2.524919	-2.580972	0.442556
16	1	0	3.765316	-1.008814	-1.027065
17	1	0	2.963657	1.293165	-1.428874
18	1	0	0.645371	0.709395	1.842896

19	1	0	-3.986620	-1.229595	-0.578673

TS_{U4}

E(B3LYP*/LB)= -857.9920925

Center Number	Atomic Number	Atomic Type	Coordinates (Angstroms) X	Y	Z
1	6	0	0.910379	0.426979	0.070830
2	16	0	2.195094	-0.832419	-0.206450
3	6	0	-0.447463	0.031012	-0.473250
4	6	0	3.653597	0.079862	0.403558
5	6	0	-0.803612	-1.373711	-0.516266
6	6	0	-2.005716	-1.773482	0.002355
7	8	0	1.147585	1.485763	0.606886
8	6	0	-2.901093	-0.831502	0.611869
9	6	0	-2.676115	0.526813	0.617769
10	6	0	-1.547770	0.948007	-0.134627
11	8	0	-1.387972	2.025770	-0.805081
12	1	0	3.291527	0.996214	0.874255
13	1	0	4.176296	-0.539746	1.133136
14	1	0	-0.096581	-2.108448	-0.888804
15	1	0	-2.280291	-2.823006	-0.002025
16	1	0	-3.821404	-1.211360	1.049009
17	1	0	-3.399712	1.241024	0.994540
18	1	0	4.315488	0.320642	-0.429407
19	1	0	-0.476969	1.147240	-1.335357

Phenol

E(B3LYP*/LB)= -858.1184042

Center Number	Atomic Number	Atomic Type	Coordinates (Angstroms) X	Y	Z
1	6	0	0.955608	0.346051	-0.000805
2	16	0	2.196216	-0.963451	-0.016683
3	6	0	-0.454352	-0.071312	0.001331
4	6	0	3.724139	0.034115	0.018704
5	6	0	-0.850656	-1.425482	0.006585
6	6	0	-2.187364	-1.784475	0.006779
7	8	0	1.295528	1.533344	0.003324
8	6	0	-3.169469	-0.779750	0.002525
9	6	0	-2.815681	0.559681	-0.001475
10	6	0	-1.461010	0.936359	-0.001627
11	8	0	-1.181320	2.247075	-0.004811
12	1	0	3.446247	1.076887	-0.143478
13	1	0	4.213868	-0.076627	0.987075
14	1	0	-0.091076	-2.200684	0.012319
15	1	0	-2.472363	-2.831155	0.011212
16	1	0	-4.220987	-1.053023	0.003193
17	1	0	-3.561192	1.347574	-0.003935
18	1	0	-0.197938	2.331356	-0.001918

19	1	0	4.383033	-0.313588	-0.777769

React’

E(B3LYP*/LB)= -5130.738309 Number of imaginary frequencies: 8

Center Number	Atomic Number	Atomic Type	Coordinates (Angstroms) X	Y	Z
1	26	0	0.420206	-3.219496	0.618159
2	26	0	-3.546765	-2.250476	-0.404182
3	6	0	1.857646	0.439383	-2.261814
4	16	0	2.419572	1.809195	-3.240751
5	6	0	0.753563	0.719273	-1.318819
6	6	0	3.907605	1.090884	-4.042674
7	6	0	-0.261660	1.591791	-1.557167
8	6	0	5.142755	1.258391	-3.138751
9	6	0	-1.318212	1.798922	-0.582530
10	7	0	6.348979	0.704968	-3.720091
11	8	0	2.370744	-0.685676	-2.311371
12	6	0	-1.331031	1.164250	0.612296
13	6	0	7.260372	1.506559	-4.350616
14	8	0	7.059098	2.700879	-4.559056
15	6	0	-0.282174	0.180958	0.948127
16	6	0	8.552484	0.812994	-4.780645
17	6	0	0.785236	-0.060533	-0.058944
18	6	0	9.779483	1.692840	-4.507164
19	6	0	7.918575	-0.140195	-0.039372
20	6	0	8.231768	-1.636210	-0.151129
21	6	0	6.976594	-2.507706	-0.306464
22	6	0	6.195711	-2.163468	-1.570518
23	8	0	6.769542	-1.969466	-2.650442
24	7	0	4.853097	-2.100719	-1.432148
25	6	0	4.035161	2.447510	3.210296
26	6	0	2.989617	1.634435	3.985856
27	8	0	1.883766	2.094875	4.300222
28	6	0	4.006341	2.020749	1.712139
29	8	0	4.685369	0.753686	1.650663
30	6	0	4.734729	3.029905	0.830991
31	7	0	3.345130	0.342351	4.226042
32	6	0	2.414500	-0.710644	4.669798
33	6	0	3.105464	-2.076696	4.623734
34	6	0	3.778482	-2.402010	3.276440
35	6	0	2.874432	-2.250033	2.052353
36	8	0	1.709550	-2.769044	2.147434
37	8	0	3.310657	-1.664930	1.029411
38	6	0	-1.582378	2.847253	5.713732
39	6	0	-1.558678	1.960106	4.463878
40	8	0	-0.699322	0.825091	4.579729
41	6	0	-0.908412	-4.477965	4.651417
42	6	0	-0.874007	-2.951933	4.452998
43	6	0	-2.020743	-2.303748	3.666838
44	6	0	-2.103661	-2.567370	2.173658
45	8	0	-1.319468	-3.406896	1.617614

46	8	0	-3.002933	-1.946736	1.527870
47	6	0	3.106081	-6.854269	3.310139
48	6	0	2.510898	-5.799719	2.365073
49	6	0	1.485980	-6.285930	1.383704
50	7	0	0.740758	-5.406105	0.600752
51	6	0	1.160667	-7.568805	1.024675
52	6	0	-0.000390	-6.145397	-0.211788
53	7	0	0.220247	-7.460112	0.014408
54	6	0	1.550431	8.347917	3.824974
55	6	0	2.465150	7.122739	3.644568
56	6	0	2.216212	6.418383	2.326192
57	6	0	1.473879	5.230207	2.271872
58	6	0	2.673906	6.972870	1.121018
59	6	0	1.176467	4.627396	1.046179
60	6	0	2.385896	6.369882	-0.103609
61	6	0	1.627827	5.196655	-0.145963
62	6	0	-7.301026	5.786122	-1.241232
63	6	0	-7.042333	6.165904	0.234500
64	6	0	-5.655200	5.902742	0.781787
65	6	0	-4.740703	6.951753	0.950734
66	6	0	-5.267705	4.615963	1.182550
67	6	0	-3.482864	6.726551	1.513008
68	6	0	-4.010281	4.381762	1.743945
69	6	0	-3.113930	5.440172	1.912970
70	6	0	-3.249316	4.973128	-3.848261
71	6	0	-2.745623	3.523562	-3.878044
72	8	0	-1.651244	3.225075	-4.362041
73	6	0	-3.337336	5.626435	-2.454595
74	8	0	-4.066222	6.852800	-2.541858
75	6	0	-1.957990	5.844367	-1.835095
76	6	0	-7.876102	-3.955680	-2.167345
77	6	0	-6.543557	-3.446474	-1.611522
78	6	0	-5.929806	-4.330196	-0.566597
79	7	0	-4.706375	-4.015842	0.012893
80	6	0	-6.379255	-5.502574	-0.013922
81	6	0	-4.426739	-4.970052	0.885928
82	7	0	-5.415298	-5.892195	0.901573
83	7	0	-3.592901	2.571172	-3.389173
84	6	0	-3.242907	1.151400	-3.447014
85	6	0	-4.390382	0.267732	-2.944321
86	6	0	-4.622032	0.435084	-1.435677
87	8	0	-4.825306	1.570459	-0.974036
88	8	0	-4.601085	-0.633071	-0.703993
89	6	0	-3.710335	-6.274809	-3.711069
90	6	0	-2.533812	-5.541623	-4.386797
91	6	0	-2.542867	-4.013705	-4.217113
92	6	0	-2.228515	-3.586987	-2.782191
93	8	0	-1.199704	-4.029625	-2.218302
94	8	0	-3.068257	-2.784142	-2.240730
95	8	0	-4.286676	1.062065	1.872650
96	8	0	1.118430	-3.072466	-1.328709
97	8	0	-0.278499	-1.050127	0.141059
98	1	0	8.780361	-1.974033	0.736766
99	1	0	8.881421	-1.812644	-1.014802

100	1	0	7.265283	-3.564312	-0.381428
101	1	0	6.327312	-2.412002	0.571687
102	1	0	4.399172	-2.089580	-0.523473
103	1	0	4.292095	-1.807798	-2.220471
104	1	0	7.490002	0.235942	-0.973688
105	1	0	2.969589	1.914724	1.365867
106	1	0	4.762226	2.676569	-0.203765
107	1	0	5.767993	3.152735	1.172805
108	1	0	4.235240	4.002327	0.851309
109	1	0	4.130045	0.048981	1.271405
110	1	0	3.862794	3.513018	3.353958
111	1	0	2.353879	-2.838335	4.849357
112	1	0	3.866079	-2.132111	5.411821
113	1	0	4.113294	-3.446731	3.297336
114	1	0	4.676881	-1.801593	3.109872
115	1	0	1.523554	-0.712582	4.032500
116	1	0	-2.557764	1.580225	4.228612
117	1	0	-1.246065	2.575778	3.606246
118	1	0	0.212770	1.175623	4.605894
119	1	0	-1.939594	3.847367	5.437520
120	1	0	-0.877634	-2.469978	5.437785
121	1	0	0.074891	-2.680836	3.981987
122	1	0	-2.997800	-2.582191	4.086489
123	1	0	-1.952711	-1.215376	3.787478
124	1	0	-0.618123	-5.015897	3.745573
125	1	0	2.075967	-4.979451	2.941950
126	1	0	3.325399	-5.344421	1.784674
127	1	0	1.511641	-8.524031	1.379755
128	1	0	-0.205228	-8.223165	-0.489154
129	1	0	-0.670743	-5.755043	-0.967224
130	1	0	3.472100	-7.731942	2.766015
131	1	0	2.299034	6.419686	4.468671
132	1	0	3.513720	7.440436	3.709330
133	1	0	3.261276	7.888282	1.144011
134	1	0	1.142602	4.757325	3.193122
135	1	0	2.753570	6.813503	-1.024811
136	1	0	0.588465	3.714206	1.025975
137	1	0	1.401900	4.727290	-1.099022
138	1	0	0.500773	8.063216	3.696427
139	1	0	-7.771941	5.631648	0.857338
140	1	0	-7.266902	7.232799	0.354972
141	1	0	-5.022482	7.956375	0.645172
142	1	0	-5.959835	3.785156	1.068877
143	1	0	-2.793692	7.557006	1.644476
144	1	0	-3.753527	3.375452	2.060381
145	1	0	-2.134654	5.266405	2.351199
146	1	0	-6.617046	6.309061	-1.915646
147	1	0	-3.940842	5.004086	-1.784763
148	1	0	-1.357995	6.514233	-2.465097
149	1	0	-1.405335	4.904061	-1.747489
150	1	0	-2.053684	6.283737	-0.840977
151	1	0	-3.527709	7.474492	-3.050955
152	1	0	-2.545288	5.539845	-4.466710
153	1	0	-5.816129	-3.327497	-2.423782

154	1	0	-6.667175	-2.443270	-1.185643
155	1	0	-7.274628	-6.078689	-0.181156
156	1	0	-5.450587	-6.709354	1.491863
157	1	0	-3.535826	-5.020611	1.495013
158	1	0	-8.625358	-4.065120	-1.375610
159	1	0	-4.159173	-0.775855	-3.164849
160	1	0	-5.318155	0.534062	-3.466635
161	1	0	-2.347958	0.945721	-2.848964
162	1	0	3.698024	0.042453	-4.264387
163	1	0	4.036832	1.638135	-4.979236
164	1	0	-0.315039	2.140348	-2.495978
165	1	0	4.956557	0.769215	-2.177825
166	1	0	5.314334	2.323262	-2.962795
167	1	0	-2.115983	2.492548	-0.825226
168	1	0	-2.126940	1.349074	1.329801
169	1	0	-0.087569	0.003749	2.003291
170	1	0	8.644853	-0.162733	-4.291414
171	1	0	8.472181	0.622020	-5.859000
172	1	0	1.750075	-0.442100	0.262305
173	1	0	10.684444	1.241629	-4.925243
174	1	0	9.933855	1.829102	-3.431813
175	1	0	9.635988	2.679997	-4.952268
176	1	0	-3.957369	-5.830769	-2.739819
177	1	0	-2.519909	-5.784699	-5.455397
178	1	0	-1.587201	-5.907316	-3.974198
179	1	0	-3.501265	-3.582206	-4.518837
180	1	0	-1.765967	-3.577465	-4.857796
181	1	0	-4.567759	1.290988	0.963986
182	1	0	-4.100456	0.114325	1.812650
183	1	0	8.825485	0.434148	0.150773
184	1	0	7.202016	0.072100	0.761867
185	1	0	5.049841	2.140029	3.463578
186	1	0	2.078474	-0.493978	5.683591
187	1	0	3.964370	-6.408856	3.813036
188	1	0	2.385187	-7.190115	4.061237
189	1	0	-0.226260	-4.733000	5.462755
190	1	0	-1.906680	-4.822009	4.951492
191	1	0	-3.407047	-7.305672	-3.527977
192	1	0	-4.615431	-6.266458	-4.325188
193	1	0	-7.762191	-4.924998	-2.664307
194	1	0	-8.269904	-3.247311	-2.896100
195	1	0	-2.968841	0.908374	-4.473619
196	1	0	-7.204628	4.708218	-1.405604
197	1	0	-8.327817	6.088381	-1.448509
198	1	0	-4.224961	5.023439	-4.331568
199	1	0	1.773523	9.120129	3.081590
200	1	0	1.651047	8.790181	4.816194
201	1	0	-0.567737	2.966018	6.108961
202	1	0	-2.234411	2.509629	6.519424
203	1	0	4.177189	0.051315	3.728818
204	1	0	6.566611	-0.271358	-3.519592
205	1	0	-4.338326	2.815952	-2.746477
206	1	0	0.346659	-3.321734	-1.893650
207	1	0	1.515098	-2.234573	-1.654643

TS1'

E(B3LYP*/LB)= -5130.715922 Number of imaginary frequencies: 10

Center Number	Atomic Number	Atomic Type	Coordinates (Angstroms) X	Y	Z
1	26	0	-0.707254	-3.172878	0.570326
2	26	0	-3.562503	-1.291422	-0.368859
3	6	0	1.938823	0.064594	-2.280345
4	16	0	2.883033	1.316834	-3.114841
5	6	0	0.867512	0.549471	-1.358173
6	6	0	4.188732	0.295005	-3.909577
7	6	0	0.183939	1.770074	-1.493505
8	6	0	5.404573	0.111980	-2.982454
9	6	0	-0.869695	2.126832	-0.605810
10	7	0	6.419531	-0.741078	-3.568041
11	8	0	2.147682	-1.141262	-2.406260
12	6	0	-1.325897	1.250754	0.337780
13	6	0	7.540707	-0.217913	-4.153647
14	8	0	7.710894	0.990400	-4.297210
15	6	0	-0.672035	-0.082432	0.526138
16	6	0	8.563802	-1.249148	-4.631988
17	6	0	0.540116	-0.283784	-0.296867
18	6	0	10.005483	-0.774931	-4.406639
19	6	0	7.670999	-2.175253	-0.005105
20	6	0	7.577466	-3.692004	-0.196239
21	6	0	6.130472	-4.182236	-0.363748
22	6	0	5.456111	-3.554242	-1.578848
23	8	0	6.037466	-3.485417	-2.670194
24	7	0	4.198729	-3.098047	-1.390893
25	6	0	4.527874	1.242393	3.277755
26	6	0	3.288437	0.682011	3.979606
27	8	0	2.293400	1.379666	4.217971
28	6	0	4.430781	0.969436	1.748216
29	8	0	4.686088	-0.426310	1.541235
30	6	0	5.476252	1.775888	0.985078
31	7	0	3.322127	-0.652494	4.247787
32	6	0	2.132546	-1.429869	4.639137
33	6	0	2.379914	-2.928593	4.477049
34	6	0	2.871532	-3.327793	3.070079
35	6	0	2.008756	-2.834626	1.903340
36	8	0	0.748846	-3.068425	1.978836
37	8	0	2.562560	-2.251187	0.942104
38	6	0	-0.838890	3.005811	5.741880
39	6	0	-1.052493	2.191742	4.459345
40	8	0	-0.550531	0.858573	4.536156
41	6	0	-2.040285	-4.221674	4.492235
42	6	0	-1.730778	-2.715251	4.477426
43	6	0	-2.752996	-1.801164	3.800401
44	6	0	-2.936904	-1.991720	2.310303
45	8	0	-2.465662	-3.020739	1.722765
46	8	0	-3.601522	-1.108450	1.682129

47	6	0	1.254106	-7.513111	3.123831
48	6	0	0.850031	-6.277881	2.297256
49	6	0	-0.329703	-6.464172	1.384548
50	7	0	-0.894675	-5.419378	0.650174
51	6	0	-0.984650	-7.625508	1.062748
52	6	0	-1.850082	-5.951979	-0.096382
53	7	0	-1.943015	-7.283150	0.126473
54	6	0	3.622290	7.565835	4.026360
55	6	0	4.189535	6.149523	3.823153
56	6	0	3.797042	5.566514	2.480402
57	6	0	2.787068	4.599445	2.380882
58	6	0	4.400617	6.022681	1.298283
59	6	0	2.378650	4.117511	1.133944
60	6	0	3.999405	5.539584	0.052582
61	6	0	2.980409	4.587379	-0.034581
62	6	0	-5.511913	7.469675	-1.182636
63	6	0	-5.182030	7.696264	0.308335
64	6	0	-3.921724	7.036258	0.827796
65	6	0	-2.776889	7.795870	1.105985
66	6	0	-3.882292	5.658358	1.087641
67	6	0	-1.630620	7.202266	1.638453
68	6	0	-2.740625	5.057855	1.623567
69	6	0	-1.609895	5.830924	1.901735
70	6	0	-1.762887	5.709283	-3.774797
71	6	0	-1.637034	4.183207	-3.756543
72	8	0	-0.587288	3.619190	-4.083420
73	6	0	-1.682535	6.394347	-2.392164
74	8	0	-2.109371	7.753036	-2.502068
75	6	0	-0.285293	6.300459	-1.782416
76	6	0	-8.540215	-1.777605	-2.370567
77	6	0	-7.129101	-1.566175	-1.787951
78	6	0	-6.713775	-2.575218	-0.759285
79	7	0	-5.458104	-2.566699	-0.156152
80	6	0	-7.441717	-3.617591	-0.242907
81	6	0	-5.440411	-3.580374	0.695396
82	7	0	-6.619567	-4.244310	0.675550
83	7	0	-2.738688	3.466929	-3.393834
84	6	0	-2.738112	2.004509	-3.474514
85	6	0	-4.058243	1.401206	-2.983006
86	6	0	-4.212913	1.612689	-1.471810
87	8	0	-4.452849	2.748525	-1.034799
88	8	0	-4.033651	0.586106	-0.702651
89	6	0	-5.081343	-5.047497	-3.938520
90	6	0	-3.704165	-4.644856	-4.509964
91	6	0	-3.288975	-3.191081	-4.249718
92	6	0	-2.979265	-2.923510	-2.775812
93	8	0	-2.229140	-3.708653	-2.140548
94	8	0	-3.521486	-1.881925	-2.273421
95	8	0	-4.049218	1.941914	1.945822
96	8	0	0.325622	-3.378120	-1.327715
97	8	0	-1.397957	-1.198391	-0.077937
98	1	0	8.023594	-4.212021	0.660373
99	1	0	8.146136	-3.989433	-1.083477
100	1	0	6.120203	-5.269435	-0.514118

101	1	0	5.547250	-3.976479	0.541622
102	1	0	3.776319	-2.998789	-0.473647
103	1	0	3.724335	-2.633143	-2.153748
104	1	0	7.355731	-1.651844	-0.913163
105	1	0	3.429368	1.235817	1.385515
106	1	0	5.419374	1.550323	-0.083007
107	1	0	6.481348	1.513052	1.332482
108	1	0	5.323205	2.849677	1.122705
109	1	0	3.882506	-0.908648	1.275937
110	1	0	4.626179	2.303699	3.504068
111	1	0	1.442703	-3.446981	4.698697
112	1	0	3.117985	-3.273142	5.211907
113	1	0	2.901477	-4.421904	3.011979
114	1	0	3.892582	-2.982497	2.887138
115	1	0	1.292680	-1.127241	4.002427
116	1	0	-2.116051	2.118503	4.211087
117	1	0	-0.570097	2.724104	3.624217
118	1	0	0.422590	0.935578	4.544410
119	1	0	-0.904171	4.075705	5.506709
120	1	0	-1.636268	-2.360761	5.510660
121	1	0	-0.754587	-2.559413	4.008761
122	1	0	-3.746707	-1.914526	4.257742
123	1	0	-2.464976	-0.755963	3.959189
124	1	0	-1.797819	-4.706825	3.546811
125	1	0	0.648493	-5.429610	2.957871
126	1	0	1.700840	-5.970209	1.674923
127	1	0	-0.851268	-8.639697	1.402091
128	1	0	-2.575656	-7.914813	-0.339676
129	1	0	-2.446216	-5.407106	-0.816053
130	1	0	1.421585	-8.386597	2.484248
131	1	0	3.827811	5.493304	4.622821
132	1	0	5.282912	6.180858	3.913423
133	1	0	5.193320	6.765473	1.356072
134	1	0	2.328875	4.200810	3.282447
135	1	0	4.483237	5.902655	-0.849933
136	1	0	1.588941	3.373019	1.082002
137	1	0	2.670948	4.209148	-1.004756
138	1	0	2.537101	7.568230	3.879074
139	1	0	-6.034107	7.346789	0.906310
140	1	0	-5.110375	8.776237	0.485475
141	1	0	-2.788946	8.865463	0.910719
142	1	0	-4.755072	5.044152	0.881697
143	1	0	-0.757745	7.812530	1.856489
144	1	0	-2.759828	3.995727	1.848303
145	1	0	-0.719814	5.371412	2.323520
146	1	0	-4.705735	7.829523	-1.828454
147	1	0	-2.408557	5.939440	-1.709715
148	1	0	0.447060	6.802845	-2.428088
149	1	0	0.039171	5.260902	-1.678100
150	1	0	-0.270332	6.768811	-0.797049
151	1	0	-1.437161	8.230653	-3.007724
152	1	0	-0.931529	6.061002	-4.395320
153	1	0	-6.386376	-1.572745	-2.594882
154	1	0	-7.063561	-0.566611	-1.339357

155	1	0	-8.446746	-3.954778	-0.437609
156	1	0	-6.858895	-5.039138	1.247935
157	1	0	-4.600786	-3.858423	1.316108
158	1	0	-9.310636	-1.732984	-1.594190
159	1	0	-4.059807	0.333444	-3.209744
160	1	0	-4.901217	1.879008	-3.495392
161	1	0	-1.914027	1.593543	-2.883213
162	1	0	3.733367	-0.659772	-4.180310
163	1	0	4.471884	0.828100	-4.819993
164	1	0	0.412878	2.443449	-2.318185
165	1	0	5.078558	-0.320274	-2.031095
166	1	0	5.860021	1.085630	-2.786172
167	1	0	-1.359337	3.086717	-0.728417
168	1	0	-2.162502	1.513452	0.983349
169	1	0	-0.512154	-0.253024	1.601517
170	1	0	8.381361	-2.216431	-4.151173
171	1	0	8.390679	-1.395214	-5.706773
172	1	0	1.150512	-1.156245	-0.083401
173	1	0	10.718734	-1.466517	-4.865066
174	1	0	10.235464	-0.708040	-3.338366
175	1	0	10.143571	0.218133	-4.840176
176	1	0	-5.272180	-4.576323	-2.967749
177	1	0	-3.688384	-4.838039	-5.588789
178	1	0	-2.930501	-5.282787	-4.069337
179	1	0	-4.053843	-2.484340	-4.583572
180	1	0	-2.372509	-2.971304	-4.813417
181	1	0	-4.309679	2.164313	1.033317
182	1	0	-4.075108	0.972454	1.959031
183	1	0	8.691542	-1.856309	0.207914
184	1	0	7.028365	-1.825495	0.810852
185	1	0	5.426528	0.680049	3.531665
186	1	0	1.848270	-1.158300	5.655457
187	1	0	2.189899	-7.313268	3.645938
188	1	0	0.490812	-7.772787	3.863916
189	1	0	-1.458911	-4.661315	5.302879
190	1	0	-3.097641	-4.412636	4.716782
191	1	0	-5.054062	-6.125582	-3.780255
192	1	0	-5.906853	-4.794027	-4.609263
193	1	0	-8.625563	-2.735768	-2.892959
194	1	0	-8.729100	-0.975263	-3.083735
195	1	0	-2.519746	1.723519	-4.504833
196	1	0	-5.689104	6.411084	-1.398714
197	1	0	-6.424415	8.028857	-1.390642
198	1	0	-2.686001	6.018445	-4.265069
199	1	0	4.051802	8.270135	3.306481
200	1	0	3.817424	7.944506	5.029735
201	1	0	0.164978	2.822401	6.139709
202	1	0	-1.567636	2.827326	6.532682
203	1	0	4.109654	-1.149910	3.856571
204	1	0	6.331684	-1.747595	-3.424577
205	1	0	-3.510658	3.902373	-2.902865
206	1	0	-0.456965	-3.504726	-1.909237
207	1	0	0.848260	-2.638236	-1.686548

Int1’

E(B3LYP*/LB)= -5130.725242 Number of imaginary frequencies: 7

Center Number	Atomic Number	Atomic Type	Coordinates (Angstroms) X	Y	Z
1	26	0	-0.664585	-3.115835	0.660826
2	26	0	-3.496011	-1.230259	-0.297210
3	6	0	1.970165	0.093094	-2.310896
4	16	0	2.883527	1.353908	-3.173990
5	6	0	0.884486	0.570268	-1.404093
6	6	0	4.187347	0.339123	-3.973431
7	6	0	0.200230	1.796722	-1.568135
8	6	0	5.394292	0.145100	-3.039546
9	6	0	-0.841575	2.140084	-0.661777
10	7	0	6.414824	-0.705732	-3.618355
11	8	0	2.208747	-1.108972	-2.429514
12	6	0	-1.291518	1.265049	0.288083
13	6	0	7.494808	-0.175340	-4.265907
14	8	0	7.617709	1.030528	-4.471202
15	6	0	-0.684740	-0.090895	0.438009
16	6	0	8.544338	-1.188554	-4.720962
17	6	0	0.557956	-0.266897	-0.356018
18	6	0	9.966592	-0.682941	-4.448363
19	6	0	7.694083	-2.006557	0.008997
20	6	0	7.625898	-3.529728	-0.139938
21	6	0	6.189274	-4.052261	-0.297722
22	6	0	5.510803	-3.476575	-1.536795
23	8	0	6.101571	-3.423146	-2.623978
24	7	0	4.241626	-3.045975	-1.371163
25	6	0	4.540959	1.449320	3.241863
26	6	0	3.338300	0.899009	4.025646
27	8	0	2.380021	1.607198	4.359969
28	6	0	4.396100	1.083720	1.738197
29	8	0	4.665857	-0.326014	1.632854
30	6	0	5.398438	1.854954	0.885610
31	7	0	3.374812	-0.444555	4.264292
32	6	0	2.193368	-1.219173	4.690965
33	6	0	2.437646	-2.721353	4.559207
34	6	0	2.914360	-3.164407	3.160061
35	6	0	2.053107	-2.736392	1.964452
36	8	0	0.784994	-2.946722	2.054425
37	8	0	2.614379	-2.240728	0.963654
38	6	0	-0.822796	3.204411	5.718481
39	6	0	-0.987291	2.279092	4.508571
40	8	0	-0.453101	0.974151	4.717346
41	6	0	-1.945156	-4.065199	4.653352
42	6	0	-1.663566	-2.554658	4.585743
43	6	0	-2.708493	-1.685067	3.882074
44	6	0	-2.911195	-1.956803	2.407802
45	8	0	-2.400558	-2.983646	1.860805
46	8	0	-3.635923	-1.135747	1.749599
47	6	0	1.376747	-7.347282	3.329726

48	6	0	0.949665	-6.142351	2.469583
49	6	0	-0.219498	-6.380015	1.554740
50	7	0	-0.807385	-5.370067	0.788860
51	6	0	-0.839438	-7.567076	1.257888
52	6	0	-1.740772	-5.949287	0.049304
53	7	0	-1.798986	-7.276546	0.306045
54	6	0	3.563037	7.777128	3.849273
55	6	0	4.101296	6.342896	3.696576
56	6	0	3.689974	5.710512	2.381798
57	6	0	2.698262	4.720569	2.335643
58	6	0	4.261533	6.136980	1.172967
59	6	0	2.275864	4.186457	1.114543
60	6	0	3.846867	5.602216	-0.046995
61	6	0	2.846220	4.627241	-0.080917
62	6	0	-5.620816	7.443293	-1.261715
63	6	0	-5.286603	7.699182	0.223884
64	6	0	-4.033310	7.036497	0.756477
65	6	0	-2.867752	7.780981	0.984224
66	6	0	-4.020626	5.671094	1.077674
67	6	0	-1.727478	7.184088	1.525723
68	6	0	-2.884049	5.066151	1.619479
69	6	0	-1.732283	5.824789	1.846100
70	6	0	-1.875907	5.668104	-3.849748
71	6	0	-1.748263	4.140556	-3.853103
72	8	0	-0.742126	3.574751	-4.283848
73	6	0	-1.795851	6.347635	-2.466772
74	8	0	-2.231710	7.704581	-2.569163
75	6	0	-0.394259	6.258464	-1.866338
76	6	0	-8.543677	-1.866561	-2.198359
77	6	0	-7.108403	-1.630612	-1.672945
78	6	0	-6.619554	-2.629270	-0.663845
79	7	0	-5.348391	-2.586270	-0.084990
80	6	0	-7.296163	-3.702561	-0.142405
81	6	0	-5.277780	-3.609858	0.753014
82	7	0	-6.432365	-4.312422	0.747928
83	7	0	-2.820314	3.422882	-3.397972
84	6	0	-2.801208	1.959782	-3.451512
85	6	0	-4.107527	1.334968	-2.942483
86	6	0	-4.280860	1.540615	-1.438140
87	8	0	-4.519409	2.650177	-0.969352
88	8	0	-4.156752	0.479343	-0.663007
89	6	0	-5.059623	-5.129846	-3.723679
90	6	0	-3.700796	-4.717424	-4.328918
91	6	0	-3.313800	-3.249695	-4.107727
92	6	0	-3.007204	-2.945810	-2.641997
93	8	0	-2.266268	-3.707459	-1.977365
94	8	0	-3.553981	-1.884510	-2.171849
95	8	0	-4.104169	1.898622	2.036421
96	8	0	0.335467	-3.331647	-1.251350
97	8	0	-1.557169	-1.206184	-0.067380
98	1	0	8.079206	-4.016921	0.732125
99	1	0	8.202480	-3.841169	-1.017235
100	1	0	6.200913	-5.143990	-0.411104
101	1	0	5.596828	-3.826613	0.596790

102	1	0	3.807967	-2.946012	-0.459107
103	1	0	3.764775	-2.601125	-2.144844
104	1	0	7.364645	-1.514234	-0.911388
105	1	0	3.377202	1.301506	1.393802
106	1	0	5.322548	1.545360	-0.160138
107	1	0	6.419220	1.649070	1.225399
108	1	0	5.216103	2.931695	0.941631
109	1	0	3.915369	-0.809530	1.245399
110	1	0	4.631763	2.521687	3.407660
111	1	0	1.502499	-3.232284	4.806836
112	1	0	3.185243	-3.049121	5.291967
113	1	0	2.955121	-4.259933	3.144530
114	1	0	3.932823	-2.822501	2.957867
115	1	0	1.339242	-0.936601	4.061665
116	1	0	-2.044563	2.156007	4.250853
117	1	0	-0.503233	2.747832	3.637327
118	1	0	0.516911	1.082506	4.724535
119	1	0	-0.956081	4.246812	5.402146
120	1	0	-1.569806	-2.159207	5.603612
121	1	0	-0.694402	-2.395609	4.104480
122	1	0	-3.693816	-1.781667	4.360499
123	1	0	-2.427762	-0.630393	3.984077
124	1	0	-1.703939	-4.573767	3.719901
125	1	0	0.725305	-5.281915	3.106495
126	1	0	1.796643	-5.832382	1.843529
127	1	0	-0.680268	-8.568064	1.624147
128	1	0	-2.408032	-7.938438	-0.149465
129	1	0	-2.349822	-5.442280	-0.686545
130	1	0	1.550200	-8.236982	2.714412
131	1	0	3.733843	5.725855	4.524193
132	1	0	5.195820	6.357568	3.779480
133	1	0	5.040323	6.896452	1.189263
134	1	0	2.268320	4.343111	3.260025
135	1	0	4.306448	5.942354	-0.970970
136	1	0	1.501714	3.424153	1.100688
137	1	0	2.526314	4.206270	-1.029639
138	1	0	2.478413	7.797114	3.698955
139	1	0	-6.141727	7.371714	0.829878
140	1	0	-5.203929	8.782031	0.376769
141	1	0	-2.858393	8.840764	0.741532
142	1	0	-4.914386	5.073038	0.917131
143	1	0	-0.837919	7.782584	1.705288
144	1	0	-2.918183	4.014424	1.887857
145	1	0	-0.845585	5.362983	2.271917
146	1	0	-4.821797	7.799162	-1.918243
147	1	0	-2.514996	5.887146	-1.780541
148	1	0	0.331882	6.763278	-2.517046
149	1	0	-0.066144	5.219880	-1.763113
150	1	0	-0.373246	6.726781	-0.881202
151	1	0	-1.563929	8.188110	-3.075028
152	1	0	-1.054275	6.029375	-4.477010
153	1	0	-6.404711	-1.621654	-2.514321
154	1	0	-7.044892	-0.629147	-1.228648
155	1	0	-8.294447	-4.069312	-0.317007

156	1	0	-6.631533	-5.121166	1.316445
157	1	0	-4.416082	-3.862205	1.353947
158	1	0	-9.286887	-1.801632	-1.398007
159	1	0	-4.094874	0.267070	-3.167071
160	1	0	-4.965400	1.795146	-3.446580
161	1	0	-1.966216	1.573020	-2.860976
162	1	0	3.734704	-0.613586	-4.255657
163	1	0	4.481467	0.880708	-4.875268
164	1	0	0.423325	2.456837	-2.400914
165	1	0	5.059663	-0.297593	-2.096897
166	1	0	5.845305	1.118011	-2.829003
167	1	0	-1.318309	3.110992	-0.753854
168	1	0	-2.108849	1.535790	0.951569
169	1	0	-0.521745	-0.302946	1.504340
170	1	0	8.369573	-2.160819	-4.247731
171	1	0	8.407521	-1.330323	-5.801160
172	1	0	1.187089	-1.120650	-0.133248
173	1	0	10.711217	-1.356501	-4.883483
174	1	0	10.159891	-0.613021	-3.372908
175	1	0	10.095395	0.313629	-4.876875
176	1	0	-5.241881	-4.639578	-2.760784
177	1	0	-3.699062	-4.933112	-5.403346
178	1	0	-2.907365	-5.330068	-3.887973
179	1	0	-4.093883	-2.566708	-4.455433
180	1	0	-2.403672	-3.022367	-4.678468
181	1	0	-4.361335	2.135921	1.129335
182	1	0	-4.080622	0.929564	2.026829
183	1	0	8.712577	-1.669972	0.203851
184	1	0	7.052979	-1.645321	0.821181
185	1	0	5.449125	0.904377	3.499813
186	1	0	1.915754	-0.926922	5.703372
187	1	0	2.315102	-7.123442	3.837287
188	1	0	0.622858	-7.595066	4.083342
189	1	0	-1.350202	-4.478104	5.468196
190	1	0	-2.997160	-4.267700	4.893459
191	1	0	-5.017089	-6.203396	-3.540105
192	1	0	-5.900582	-4.903968	-4.385137
193	1	0	-8.641518	-2.837828	-2.693321
194	1	0	-8.749828	-1.083887	-2.928395
195	1	0	-2.589559	1.656928	-4.477031
196	1	0	-5.787669	6.378803	-1.457154
197	1	0	-6.542328	7.985999	-1.473558
198	1	0	-2.807709	5.953989	-4.337693
199	1	0	4.008999	8.449262	3.108993
200	1	0	3.763371	8.182041	4.841314
201	1	0	0.189601	3.114459	6.126704
202	1	0	-1.541285	3.035860	6.520748
203	1	0	4.087687	-0.930897	3.734997
204	1	0	6.354959	-1.708350	-3.438995
205	1	0	-3.555826	3.866167	-2.862465
206	1	0	-0.440509	-3.473726	-1.832180
207	1	0	0.853368	-2.590602	-1.617676

TS2’

E(B3LYP*/LB)= -5130.721237 Number of imaginary frequencies: 8

Center Number	Atomic Number	Atomic Type	Coordinates (Angstroms) X	Y	Z
1	26	0	-0.529214	-3.162581	0.579912
2	26	0	-3.387194	-1.464792	-0.386737
3	6	0	1.966602	0.151523	-2.305885
4	16	0	2.828569	1.435931	-3.182489
5	6	0	0.882165	0.600767	-1.383773
6	6	0	4.179327	0.471300	-3.968902
7	6	0	0.127082	1.774974	-1.567500
8	6	0	5.396351	0.356522	-3.033997
9	6	0	-0.913554	2.089540	-0.663984
10	7	0	6.468844	-0.430213	-3.609524
11	8	0	2.242893	-1.043203	-2.414860
12	6	0	-1.248264	1.236946	0.367175
13	6	0	7.511475	0.166178	-4.261862
14	8	0	7.550458	1.375093	-4.481258
15	6	0	-0.511523	0.015185	0.549622
16	6	0	8.630240	-0.776464	-4.703972
17	6	0	0.625806	-0.217306	-0.289195
18	6	0	10.014688	-0.185219	-4.405928
19	6	0	7.780515	-1.674990	0.018289
20	6	0	7.783503	-3.199472	-0.132609
21	6	0	6.372939	-3.786553	-0.293739
22	6	0	5.669989	-3.248001	-1.535843
23	8	0	6.260134	-3.161091	-2.621220
24	7	0	4.377218	-2.892022	-1.376134
25	6	0	4.442947	1.581911	3.272511
26	6	0	3.261328	0.962145	4.033806
27	8	0	2.261692	1.617224	4.356782
28	6	0	4.329072	1.235475	1.760947
29	8	0	4.674994	-0.153774	1.628660
30	6	0	5.295061	2.074446	0.930504
31	7	0	3.363929	-0.379354	4.263242
32	6	0	2.218318	-1.215709	4.671240
33	6	0	2.533499	-2.702843	4.524746
34	6	0	3.038605	-3.105643	3.123710
35	6	0	2.168243	-2.687875	1.930474
36	8	0	0.911208	-2.947547	2.002171
37	8	0	2.721095	-2.143025	0.947351
38	6	0	-1.015308	3.042969	5.733523
39	6	0	-1.137612	2.141283	4.500020
40	8	0	-0.542802	0.857259	4.671142
41	6	0	-1.777126	-4.257979	4.568724
42	6	0	-1.603327	-2.730336	4.536297
43	6	0	-2.696818	-1.929044	3.823928
44	6	0	-2.846408	-2.197667	2.340690
45	8	0	-2.266798	-3.194029	1.807944
46	8	0	-3.594198	-1.409483	1.669855
47	6	0	1.708621	-7.358013	3.226864
48	6	0	1.234128	-6.158346	2.381303

49	6	0	0.095587	-6.430537	1.436980
50	7	0	-0.525295	-5.432599	0.680604
51	6	0	-0.456357	-7.639574	1.097883
52	6	0	-1.410556	-6.040090	-0.094409
53	7	0	-1.407509	-7.374589	0.130128
54	6	0	3.155430	7.846532	3.953093
55	6	0	3.761988	6.441700	3.784973
56	6	0	3.391164	5.811440	2.456975
57	6	0	2.469090	4.757422	2.388367
58	6	0	3.929656	6.303398	1.257820
59	6	0	2.079685	4.226342	1.154938
60	6	0	3.548804	5.771243	0.025970
61	6	0	2.614967	4.733224	-0.030455
62	6	0	-5.968682	7.134804	-1.225793
63	6	0	-5.667904	7.370989	0.270390
64	6	0	-4.375975	6.785544	0.801093
65	6	0	-3.263513	7.603464	1.042894
66	6	0	-4.273863	5.420846	1.108466
67	6	0	-2.088963	7.078749	1.585679
68	6	0	-3.102003	4.887859	1.650776
69	6	0	-2.004633	5.718877	1.892443
70	6	0	-2.125619	5.576768	-3.809884
71	6	0	-1.919775	4.058297	-3.821395
72	8	0	-0.874733	3.548601	-4.231196
73	6	0	-2.090467	6.245528	-2.419068
74	8	0	-2.580179	7.584040	-2.515256
75	6	0	-0.695133	6.207001	-1.799300
76	6	0	-8.430491	-2.292707	-2.300753
77	6	0	-6.996228	-2.019758	-1.789595
78	6	0	-6.465482	-3.010495	-0.793324
79	7	0	-5.200532	-2.915207	-0.207161
80	6	0	-7.091342	-4.124793	-0.294480
81	6	0	-5.083776	-3.948363	0.612740
82	7	0	-6.202444	-4.707737	0.589214
83	7	0	-2.961472	3.282890	-3.393451
84	6	0	-2.872519	1.823128	-3.465061
85	6	0	-4.146814	1.128616	-2.967101
86	6	0	-4.313469	1.292768	-1.456317
87	8	0	-4.569241	2.391042	-0.964158
88	8	0	-4.161919	0.220937	-0.710607
89	6	0	-4.782643	-5.363377	-3.842843
90	6	0	-3.460668	-4.878151	-4.473616
91	6	0	-3.146838	-3.394023	-4.244177
92	6	0	-2.846347	-3.094761	-2.775097
93	8	0	-2.047776	-3.819065	-2.137191
94	8	0	-3.466206	-2.085808	-2.280156
95	8	0	-4.095593	1.624944	1.976563
96	8	0	0.520919	-3.301536	-1.314491
97	8	0	-1.552744	-1.427342	-0.176362
98	1	0	8.255948	-3.666056	0.740532
99	1	0	8.376747	-3.483561	-1.008110
100	1	0	6.434074	-4.877049	-0.403613
101	1	0	5.768494	-3.585538	0.598561
102	1	0	3.936496	-2.806435	-0.465618

103	1	0	3.880970	-2.479126	-2.154869
104	1	0	7.438068	-1.197211	-0.904950
105	1	0	3.302174	1.407562	1.413288
106	1	0	5.238745	1.784625	-0.122247
107	1	0	6.323289	1.910889	1.270802
108	1	0	5.059971	3.139452	1.009693
109	1	0	3.940729	-0.678737	1.263150
110	1	0	4.479980	2.654431	3.456746
111	1	0	1.620804	-3.258620	4.759437
112	1	0	3.289810	-3.004314	5.259861
113	1	0	3.122052	-4.198082	3.091248
114	1	0	4.044516	-2.720523	2.935610
115	1	0	1.356617	-0.966871	4.038665
116	1	0	-2.187901	1.975225	4.238888
117	1	0	-0.677249	2.656747	3.642052
118	1	0	0.420846	1.010240	4.692036
119	1	0	-1.187033	4.086572	5.440592
120	1	0	-1.558453	-2.349225	5.563026
121	1	0	-0.638858	-2.491910	4.078061
122	1	0	-3.680688	-2.110523	4.280209
123	1	0	-2.501724	-0.857556	3.945833
124	1	0	-1.489242	-4.725927	3.627710
125	1	0	0.956641	-5.322959	3.030948
126	1	0	2.076286	-5.794875	1.778155
127	1	0	-0.254824	-8.639994	1.444425
128	1	0	-1.971697	-8.054784	-0.355172
129	1	0	-2.028644	-5.546937	-0.832817
130	1	0	1.928597	-8.228979	2.599862
131	1	0	3.416415	5.795841	4.599861
132	1	0	4.853934	6.505760	3.878419
133	1	0	4.655833	7.112716	1.290856
134	1	0	2.068778	4.327878	3.303210
135	1	0	3.981932	6.163676	-0.890012
136	1	0	1.358938	3.413890	1.124985
137	1	0	2.320682	4.316742	-0.989496
138	1	0	2.072074	7.816468	3.795496
139	1	0	-6.503875	6.967348	0.856609
140	1	0	-5.663746	8.452324	0.453527
141	1	0	-3.323596	8.664177	0.811761
142	1	0	-5.124882	4.766159	0.937501
143	1	0	-1.242608	7.733682	1.776760
144	1	0	-3.066325	3.833197	1.906635
145	1	0	-1.091273	5.312654	2.318666
146	1	0	-5.181284	7.548174	-1.862510
147	1	0	-2.799799	5.749220	-1.747567
148	1	0	0.021724	6.738470	-2.438892
149	1	0	-0.331342	5.180838	-1.691134
150	1	0	-0.706095	6.674338	-0.813498
151	1	0	-1.924313	8.100939	-3.003389
152	1	0	-1.317769	5.984868	-4.426222
153	1	0	-6.302974	-1.985043	-2.639004
154	1	0	-6.954274	-1.020259	-1.338744
155	1	0	-8.069740	-4.536957	-0.480314
156	1	0	-6.364704	-5.534328	1.143475

157	1	0	-4.214845	-4.165732	1.217298
158	1	0	-9.165510	-2.257233	-1.490998
159	1	0	-4.088665	0.067730	-3.216313
160	1	0	-5.027439	1.560939	-3.456549
161	1	0	-2.022501	1.468343	-2.875174
162	1	0	3.774236	-0.507227	-4.234405
163	1	0	4.443590	1.012657	-4.880023
164	1	0	0.297681	2.420685	-2.425167
165	1	0	5.089646	-0.103076	-2.090045
166	1	0	5.784330	1.357155	-2.827181
167	1	0	-1.479012	3.004995	-0.801587
168	1	0	-2.078126	1.461673	1.033512
169	1	0	-0.508752	-0.413050	1.547235
170	1	0	8.509965	-1.761275	-4.239757
171	1	0	8.520158	-0.918557	-5.787058
172	1	0	1.290318	-1.044745	-0.065697
173	1	0	10.805808	-0.808850	-4.833183
174	1	0	10.186981	-0.110398	-3.327231
175	1	0	10.087973	0.819856	-4.827692
176	1	0	-4.966469	-4.892517	-2.870873
177	1	0	-3.471615	-5.083853	-5.550058
178	1	0	-2.626983	-5.451770	-4.055198
179	1	0	-3.964375	-2.749732	-4.579519
180	1	0	-2.253286	-3.116626	-4.818594
181	1	0	-4.380154	1.870664	1.078736
182	1	0	-4.073313	0.655445	1.955659
183	1	0	8.780233	-1.292085	0.224541
184	1	0	7.114234	-1.345536	0.823652
185	1	0	5.374805	1.078254	3.529883
186	1	0	1.920428	-0.950409	5.685308
187	1	0	2.631718	-7.095561	3.743807
188	1	0	0.959928	-7.649320	3.969853
189	1	0	-1.167782	-4.652077	5.382286
190	1	0	-2.816073	-4.538753	4.786333
191	1	0	-4.689248	-6.435889	-3.672490
192	1	0	-5.648380	-5.170576	-4.482710
193	1	0	-8.505863	-3.262380	-2.802969
194	1	0	-8.669719	-1.511442	-3.022165
195	1	0	-2.639869	1.544247	-4.492852
196	1	0	-6.077965	6.068570	-1.450059
197	1	0	-6.913996	7.635024	-1.437321
198	1	0	-3.067045	5.823529	-4.300693
199	1	0	3.573458	8.548435	3.224204
200	1	0	3.329562	8.247756	4.951557
201	1	0	-0.002119	2.983483	6.145465
202	1	0	-1.729869	2.829420	6.528553
203	1	0	4.116411	-0.826239	3.754699
204	1	0	6.482678	-1.431044	-3.412175
205	1	0	-3.727308	3.682592	-2.866193
206	1	0	-0.243815	-3.455338	-1.907331
207	1	0	1.018158	-2.535848	-1.659094

Prod’

E(B3LYP*/LB)= -5130.785523 Number of imaginary frequencies: 9

Center Number	Atomic Number	Atomic Type	Coordinates (Angstroms) X	Y	Z
1	26	0	-0.745309	-3.128520	0.770242
2	26	0	-3.609687	-1.774928	-0.463751
3	6	0	1.965028	0.295592	-1.965458
4	16	0	2.878337	1.422081	-3.001448
5	6	0	0.970203	0.935860	-1.064750
6	6	0	4.105530	0.305217	-3.788342
7	6	0	0.222686	2.062123	-1.442121
8	6	0	5.384417	0.195227	-2.939021
9	6	0	-0.745354	2.563620	-0.569108
10	7	0	6.389222	-0.659785	-3.543357
11	8	0	2.163767	-0.922146	-1.966396
12	6	0	-0.953138	1.975222	0.680845
13	6	0	7.505307	-0.138650	-4.140942
14	8	0	7.678744	1.068369	-4.290664
15	6	0	-0.167529	0.889547	1.079547
16	6	0	8.523396	-1.172176	-4.627568
17	6	0	0.777791	0.359829	0.203805
18	6	0	9.966728	-0.682979	-4.448374
19	6	0	7.694072	-2.006557	0.009021
20	6	0	7.611867	-3.528694	-0.137661
21	6	0	6.167292	-4.032695	-0.288310
22	6	0	5.489723	-3.453397	-1.526928
23	8	0	6.069842	-3.423126	-2.619941
24	7	0	4.227778	-3.000134	-1.353821
25	6	0	4.540959	1.449342	3.241853
26	6	0	3.335076	0.888632	4.003100
27	8	0	2.365276	1.589283	4.318594
28	6	0	4.446418	1.126775	1.724944
29	8	0	4.774474	-0.260368	1.568814
30	6	0	5.451434	1.959323	0.935380
31	7	0	3.379023	-0.451810	4.263650
32	6	0	2.193341	-1.219105	4.690905
33	6	0	2.414531	-2.727259	4.543723
34	6	0	2.965593	-3.185694	3.170209
35	6	0	2.184980	-2.768908	1.913312
36	8	0	0.984691	-3.206667	1.800121
37	8	0	2.752638	-2.073585	1.044780
38	6	0	-0.822795	3.204424	5.718478
39	6	0	-0.972241	2.252749	4.528012
40	8	0	-0.418780	0.959550	4.763693
41	6	0	-1.945264	-4.065180	4.653545
42	6	0	-2.101758	-2.558822	4.949016
43	6	0	-3.027125	-1.756501	4.027512
44	6	0	-2.633209	-1.744087	2.559518
45	8	0	-1.435094	-2.124035	2.299261
46	8	0	-3.481719	-1.366374	1.710532
47	6	0	1.376699	-7.347249	3.329751
48	6	0	0.945694	-6.129884	2.495532
49	6	0	-0.263844	-6.331948	1.627784

50	7	0	-0.927515	-5.292814	0.964689
51	6	0	-0.880075	-7.508768	1.289717
52	6	0	-1.899449	-5.845887	0.248102
53	7	0	-1.905993	-7.182050	0.419631
54	6	0	3.563021	7.777123	3.849278
55	6	0	4.234061	6.408919	3.635300
56	6	0	3.860195	5.803753	2.297949
57	6	0	2.851950	4.834380	2.202690
58	6	0	4.471210	6.248496	1.115821
59	6	0	2.451301	4.337917	0.959532
60	6	0	4.079046	5.750102	-0.127036
61	6	0	3.061645	4.796101	-0.209607
62	6	0	-5.620905	7.443228	-1.261641
63	6	0	-5.254263	7.771788	0.202553
64	6	0	-4.097257	7.000755	0.801194
65	6	0	-2.829697	7.583171	0.936821
66	6	0	-4.281984	5.695576	1.280287
67	6	0	-1.781607	6.886420	1.542514
68	6	0	-3.239926	4.991046	1.885922
69	6	0	-1.983838	5.589692	2.019494
70	6	0	-1.875938	5.668080	-3.849742
71	6	0	-1.782035	4.136704	-3.869144
72	8	0	-0.802104	3.562238	-4.342975
73	6	0	-1.785495	6.346764	-2.468193
74	8	0	-2.253490	7.695323	-2.555392
75	6	0	-0.370290	6.289609	-1.896370
76	6	0	-8.543636	-1.866599	-2.198407
77	6	0	-7.108522	-1.700255	-1.670222
78	6	0	-6.780185	-2.578421	-0.499470
79	7	0	-5.512664	-2.634845	0.077333
80	6	0	-7.612901	-3.391335	0.226525
81	6	0	-5.592245	-3.448352	1.120270
82	7	0	-6.844120	-3.933494	1.241337
83	7	0	-2.857329	3.421965	-3.401109
84	6	0	-2.801202	1.959708	-3.451472
85	6	0	-4.037246	1.233857	-2.899064
86	6	0	-4.051685	1.227893	-1.369241
87	8	0	-4.286001	2.273069	-0.762140
88	8	0	-3.793014	0.095503	-0.753415
89	6	0	-5.059659	-5.129765	-3.723653
90	6	0	-3.805341	-4.704281	-4.508257
91	6	0	-3.525327	-3.184049	-4.539731
92	6	0	-2.970519	-2.672432	-3.209104
93	8	0	-1.749126	-2.687938	-3.007169
94	8	0	-3.878322	-2.262300	-2.365547
95	8	0	-4.080622	1.680002	2.062648
96	8	0	0.158210	-2.676666	-1.020417
97	8	0	-2.293724	-2.977590	-0.144639
98	1	0	8.062378	-4.020536	0.733342
99	1	0	8.180461	-3.849103	-1.016962
100	1	0	6.162991	-5.124919	-0.397808
101	1	0	5.582349	-3.794888	0.608114
102	1	0	3.828273	-2.831013	-0.436425
103	1	0	3.762126	-2.533847	-2.120804

104	1	0	7.367444	-1.513333	-0.911819
105	1	0	3.432506	1.325027	1.355477
106	1	0	5.404718	1.700164	-0.125360
107	1	0	6.467827	1.753592	1.289095
108	1	0	5.248828	3.028233	1.040943
109	1	0	3.994489	-0.787832	1.314246
110	1	0	4.625197	2.517630	3.438856
111	1	0	1.454694	-3.218923	4.733116
112	1	0	3.112246	-3.085048	5.310853
113	1	0	3.004305	-4.280825	3.180308
114	1	0	3.995225	-2.845121	3.030230
115	1	0	1.343184	-0.924254	4.061538
116	1	0	-2.027463	2.109089	4.271055
117	1	0	-0.491055	2.707961	3.649551
118	1	0	0.550198	1.080976	4.740262
119	1	0	-0.973595	4.238567	5.383457
120	1	0	-2.480583	-2.440562	5.971610
121	1	0	-1.116711	-2.083262	4.933507
122	1	0	-4.068130	-2.098901	4.083850
123	1	0	-3.037509	-0.707331	4.350329
124	1	0	-1.435760	-4.247142	3.706906
125	1	0	0.765637	-5.271320	3.145938
126	1	0	1.772693	-5.827852	1.841809
127	1	0	-0.679040	-8.526755	1.580579
128	1	0	-2.535265	-7.828668	-0.031626
129	1	0	-2.579733	-5.293780	-0.382719
130	1	0	1.552836	-8.228288	2.702657
131	1	0	3.936849	5.727520	4.440758
132	1	0	5.323187	6.523407	3.705398
133	1	0	5.262569	6.993130	1.171882
134	1	0	2.387094	4.448613	3.107112
135	1	0	4.569909	6.102133	-1.030345
136	1	0	1.664340	3.592261	0.905658
137	1	0	2.758589	4.401085	-1.174665
138	1	0	2.476672	7.691671	3.739954
139	1	0	-6.142846	7.596501	0.823180
140	1	0	-5.042628	8.845531	0.273068
141	1	0	-2.665331	8.591676	0.566279
142	1	0	-5.260139	5.227620	1.192925
143	1	0	-0.809067	7.359819	1.650628
144	1	0	-3.417145	3.986065	2.258541
145	1	0	-1.168611	5.051060	2.494564
146	1	0	-4.831648	7.747789	-1.953990
147	1	0	-2.481347	5.869732	-1.769117
148	1	0	0.325923	6.827396	-2.553246
149	1	0	-0.008489	5.260964	-1.814860
150	1	0	-0.341768	6.744890	-0.904987
151	1	0	-1.608900	8.193690	-3.076715
152	1	0	-1.053093	6.015375	-4.482520
153	1	0	-6.375466	-1.887601	-2.462209
154	1	0	-6.951198	-0.658565	-1.360522
155	1	0	-8.661147	-3.616641	0.121136
156	1	0	-7.167153	-4.554046	1.968132
157	1	0	-4.783226	-3.685930	1.791639

158	1	0	-9.280710	-1.757184	-1.395597
159	1	0	-4.026939	0.205662	-3.266258
160	1	0	-4.957746	1.717031	-3.247270
161	1	0	-1.926177	1.613636	-2.889767
162	1	0	3.624304	-0.663863	-3.938789
163	1	0	4.325725	0.745544	-4.763824
164	1	0	0.343561	2.504245	-2.427107
165	1	0	5.127301	-0.190353	-1.947026
166	1	0	5.828332	1.186439	-2.821880
167	1	0	-1.366215	3.399118	-0.871915
168	1	0	-1.748054	2.330992	1.327218
169	1	0	-0.296425	0.464363	2.070626
170	1	0	8.360559	-2.131385	-4.123895
171	1	0	8.323463	-1.341042	-5.694419
172	1	0	1.390848	-0.485883	0.494549
173	1	0	10.673827	-1.376535	-4.913620
174	1	0	10.223617	-0.595836	-3.387727
175	1	0	10.084572	0.303914	-4.901340
176	1	0	-5.109520	-4.621268	-2.755123
177	1	0	-3.891305	-5.060778	-5.541524
178	1	0	-2.920036	-5.201091	-4.091728
179	1	0	-4.440747	-2.637994	-4.790820
180	1	0	-2.772901	-2.975400	-5.305333
181	1	0	-4.197132	1.973457	1.138933
182	1	0	-3.885783	0.737405	1.958745
183	1	0	8.712573	-1.669970	0.203821
184	1	0	7.054200	-1.637694	0.818495
185	1	0	5.449118	0.904350	3.499824
186	1	0	1.915784	-0.926985	5.703398
187	1	0	2.315193	-7.123469	3.837245
188	1	0	0.628231	-7.609115	4.084163
189	1	0	-1.350158	-4.478109	5.468065
190	1	0	-2.911877	-4.584979	4.645649
191	1	0	-5.017071	-6.203454	-3.540132
192	1	0	-5.979039	-4.900945	-4.272132
193	1	0	-8.693453	-2.840552	-2.674333
194	1	0	-8.749861	-1.083856	-2.928358
195	1	0	-2.589572	1.656985	-4.477033
196	1	0	-5.813251	6.373012	-1.393058
197	1	0	-6.542295	7.986074	-1.473629
198	1	0	-2.807696	5.954022	-4.337719
199	1	0	3.910017	8.504543	3.108032
200	1	0	3.763381	8.182039	4.841308
201	1	0	0.189953	3.138355	6.129871
202	1	0	-1.541285	3.035851	6.520750
203	1	0	4.114861	-0.945265	3.775589
204	1	0	6.312151	-1.665567	-3.393079
205	1	0	-3.555633	3.856361	-2.812459
206	1	0	-0.531777	-2.583368	-1.729572
207	1	0	0.923460	-2.086320	-1.211784

TS3'

E(B3LYP*/LB)= -5130.717452 Number of imaginary frequencies: 10

Center Number	Atomic Number	Atomic Type	Coordinates (Angstroms) X	Y	Z
1	26	0	-1.364195	-3.003652	0.588820
2	26	0	-3.750942	-0.646122	-0.371066
3	6	0	2.103649	-0.137527	-2.392665
4	16	0	3.253507	0.998648	-3.130625
5	6	0	1.074296	0.446938	-1.470878
6	6	0	4.390171	-0.195780	-3.942960
7	6	0	0.647843	1.800605	-1.479542
8	6	0	5.448360	-0.732664	-2.962311
9	6	0	-0.360569	2.254647	-0.598717
10	7	0	6.253935	-1.786563	-3.545103
11	8	0	2.138519	-1.348843	-2.583516
12	6	0	-1.042068	1.378172	0.211205
13	6	0	7.444956	-1.513642	-4.156869
14	8	0	7.858386	-0.366965	-4.316708
15	6	0	-0.650860	-0.059133	0.283391
16	6	0	8.226145	-2.737438	-4.634028
17	6	0	0.508350	-0.418410	-0.566670
18	6	0	9.721338	-2.614001	-4.313826
19	6	0	7.126091	-3.576677	0.059044
20	6	0	6.750931	-5.036614	-0.208558
21	6	0	5.239096	-5.226345	-0.416794
22	6	0	4.727612	-4.407931	-1.597826
23	8	0	5.325796	-4.408350	-2.682035
24	7	0	3.594041	-3.702496	-1.390353
25	6	0	4.646444	0.350705	3.339935
26	6	0	3.325780	0.030943	4.048439
27	8	0	2.492576	0.906196	4.318897
28	6	0	4.495501	0.099822	1.812319
29	8	0	4.457033	-1.320912	1.613337
30	6	0	5.682664	0.673893	1.045759
31	7	0	3.101727	-1.291133	4.289091
32	6	0	1.781865	-1.834200	4.663659
33	6	0	1.741442	-3.348812	4.470303
34	6	0	2.147996	-3.800441	3.050808
35	6	0	1.377262	-3.147666	1.896178
36	8	0	0.096420	-3.164009	1.984971
37	8	0	2.014978	-2.659817	0.933928
38	6	0	-0.319009	3.072638	5.772849
39	6	0	-0.650947	2.282731	4.501653
40	8	0	-0.402781	0.884060	4.619517
41	6	0	-2.837440	-3.796552	4.464008
42	6	0	-2.254038	-2.373481	4.451009
43	6	0	-3.102960	-1.285119	3.792113
44	6	0	-3.358625	-1.449305	2.310174
45	8	0	-3.098232	-2.551197	1.723336
46	8	0	-3.864538	-0.465111	1.683242
47	6	0	-0.203204	-7.638236	3.099564
48	6	0	-0.369530	-6.348948	2.274641
49	6	0	-1.565376	-6.317002	1.364721
50	7	0	-1.949185	-5.179893	0.650963

51	6	0	-2.406776	-7.346610	1.027293
52	6	0	-2.982053	-5.532275	-0.099220
53	7	0	-3.295778	-6.832802	0.101609
54	6	0	4.930871	6.727989	4.124619
55	6	0	5.233708	5.233150	3.915526
56	6	0	4.745788	4.738677	2.568865
57	6	0	3.583115	3.962758	2.461732
58	6	0	5.419190	5.095766	1.390265
59	6	0	3.094825	3.576583	1.210216
60	6	0	4.938366	4.705883	0.140240
61	6	0	3.766844	3.950101	0.045249
62	6	0	-4.012486	8.367003	-1.158237
63	6	0	-3.660034	8.510901	0.337468
64	6	0	-2.552648	7.618087	0.858063
65	6	0	-1.297113	8.150186	1.183091
66	6	0	-2.768463	6.249159	1.075952
67	6	0	-0.290764	7.345818	1.721842
68	6	0	-1.767774	5.438171	1.617433
69	6	0	-0.524256	5.986921	1.943866
70	6	0	-0.634394	5.951076	-3.732204
71	6	0	-0.797776	4.428457	-3.721636
72	8	0	0.131855	3.677833	-4.037181
73	6	0	-0.447679	6.604671	-2.344557
74	8	0	-0.614835	8.019098	-2.451834
75	6	0	0.899123	6.253234	-1.715744
76	6	0	-8.703267	-0.145425	-2.433945
77	6	0	-7.276005	-0.239609	-1.863695
78	6	0	-7.077093	-1.326822	-0.850681
79	7	0	-5.853262	-1.562830	-0.230217
80	6	0	-7.993706	-2.227836	-0.369404
81	6	0	-6.036127	-2.580413	0.596419
82	7	0	-7.316628	-3.015348	0.543904
83	7	0	-2.019589	3.930459	-3.379320
84	6	0	-2.287095	2.492103	-3.465215
85	6	0	-3.695783	2.132683	-2.982334
86	6	0	-3.815977	2.335229	-1.467689
87	8	0	-3.809634	3.481976	-0.997201
88	8	0	-3.883302	1.272305	-0.726395
89	6	0	-5.901694	-3.995597	-3.996446
90	6	0	-4.473777	-3.852029	-4.565834
91	6	0	-3.799755	-2.504232	-4.283968
92	6	0	-3.493745	-2.306631	-2.799102
93	8	0	-2.959768	-3.238213	-2.140814
94	8	0	-3.811700	-1.172624	-2.309221
95	8	0	-3.518304	2.549458	1.924468
96	8	0	-0.420519	-3.498891	-1.343439
97	8	0	-1.692233	-1.002703	-0.043118
98	1	0	7.070854	-5.673504	0.625158
99	1	0	7.269352	-5.394905	-1.103925
100	1	0	5.021419	-6.280025	-0.632791
101	1	0	4.689613	-4.962429	0.494647
102	1	0	3.185209	-3.569640	-0.471460
103	1	0	3.248241	-3.103992	-2.129905
104	1	0	6.915927	-2.960433	-0.820925

105	1	0	3.568484	0.562375	1.449193
106	1	0	5.587698	0.449534	-0.019925
107	1	0	6.614780	0.222452	1.402574
108	1	0	5.743964	1.758835	1.168551
109	1	0	3.583592	-1.618539	1.300269
110	1	0	4.943626	1.374356	3.565704
111	1	0	0.723254	-3.686030	4.683934
112	1	0	2.402427	-3.843095	5.192943
113	1	0	1.985133	-4.881709	2.973901
114	1	0	3.213149	-3.637914	2.865944
115	1	0	1.018847	-1.368652	4.027077
116	1	0	-1.704069	2.400468	4.227451
117	1	0	-0.061091	2.698718	3.669084
118	1	0	0.567750	0.785202	4.642576
119	1	0	-0.204813	4.134790	5.521024
120	1	0	-2.079915	-2.047879	5.483247
121	1	0	-1.273828	-2.395538	3.965821
122	1	0	-4.087456	-1.205841	4.275753
123	1	0	-2.618051	-0.312730	3.933427
124	1	0	-2.687848	-4.318861	3.519465
125	1	0	-0.410126	-5.476919	2.933674
126	1	0	0.523842	-6.206219	1.652026
127	1	0	-2.445760	-8.374152	1.349940
128	1	0	-4.026927	-7.341438	-0.370870
129	1	0	-3.480505	-4.886765	-0.809675
130	1	0	-0.194910	-8.525798	2.457363
131	1	0	4.756948	4.649620	4.711068
132	1	0	6.314499	5.065996	4.008051
133	1	0	6.330185	5.686791	1.454323
134	1	0	3.064287	3.636642	3.359707
135	1	0	5.477386	4.989286	-0.759529
136	1	0	2.188363	2.980329	1.152479
137	1	0	3.393908	3.645325	-0.928528
138	1	0	3.865020	6.927195	3.971045
139	1	0	-4.568535	8.322935	0.925038
140	1	0	-3.386565	9.555537	0.528921
141	1	0	-1.111404	9.209304	1.021417
142	1	0	-3.732167	5.808507	0.834048
143	1	0	0.671501	7.782633	1.976897
144	1	0	-1.981718	4.391635	1.812984
145	1	0	0.256439	5.363014	2.370951
146	1	0	-3.146659	8.574759	-1.793639
147	1	0	-1.255137	6.289033	-1.675018
148	1	0	1.721260	6.613503	-2.348119
149	1	0	1.024454	5.171321	-1.612797
150	1	0	0.985759	6.707218	-0.727243
151	1	0	0.138595	8.366130	-2.949408
152	1	0	0.255144	6.144889	-4.341139
153	1	0	-6.556982	-0.392535	-2.677771
154	1	0	-6.997128	0.717201	-1.404404
155	1	0	-9.040128	-2.369825	-0.585041
156	1	0	-7.708714	-3.762628	1.095667
157	1	0	-5.270079	-3.015418	1.222432
158	1	0	-9.440424	0.052243	-1.649169

159	1	0	-3.893996	1.088040	-3.228360
160	1	0	-4.436798	2.766475	-3.483354
161	1	0	-1.551100	1.939669	-2.873822
162	1	0	3.775420	-1.001166	-4.349206
163	1	0	4.862749	0.349818	-4.762581
164	1	0	1.055765	2.497998	-2.207424
165	1	0	4.955771	-1.125284	-2.068733
166	1	0	6.112298	0.083770	-2.666688
167	1	0	-0.652669	3.299147	-0.621055
168	1	0	-1.853377	1.726712	0.850028
169	1	0	-0.354663	-0.206737	1.346656
170	1	0	7.802370	-3.653131	-4.207945
171	1	0	8.089903	-2.799051	-5.721797
172	1	0	0.897338	-1.422863	-0.452505
173	1	0	10.286587	-3.434079	-4.766845
174	1	0	9.895519	-2.638413	-3.233111
175	1	0	10.106425	-1.664880	-4.693835
176	1	0	-6.002229	-3.505448	-3.021900
177	1	0	-4.492123	-4.030351	-5.647170
178	1	0	-3.834536	-4.628416	-4.132452
179	1	0	-4.407828	-1.664676	-4.632555
180	1	0	-2.842119	-2.457395	-4.819737
181	1	0	-3.769294	2.868556	1.038546
182	1	0	-3.797561	1.619964	1.923413
183	1	0	8.186213	-3.455045	0.282502
184	1	0	6.552785	-3.154868	0.892536
185	1	0	5.421912	-0.370910	3.597175
186	1	0	1.543914	-1.519585	5.679442
187	1	0	0.748597	-7.619427	3.630564
188	1	0	-1.007084	-7.756024	3.832971
189	1	0	-2.355834	-4.341305	5.276248
190	1	0	-3.913232	-3.786537	4.682507
191	1	0	-6.077576	-5.060600	-3.845326
192	1	0	-6.665451	-3.587703	-4.664329
193	1	0	-8.991724	-1.061465	-2.959655
194	1	0	-8.732471	0.681824	-3.143057
195	1	0	-2.115556	2.180570	-4.495601
196	1	0	-4.383566	7.363010	-1.388998
197	1	0	-4.802452	9.087999	-1.369732
198	1	0	-1.479088	6.429618	-4.227706
199	1	0	5.485869	7.347161	3.412413
200	1	0	5.183973	7.058318	5.132095
201	1	0	0.636591	2.733083	6.186911
202	1	0	-1.075489	3.029300	6.556511
203	1	0	3.758831	-1.916199	3.842620
204	1	0	5.950370	-2.750757	-3.404215
205	1	0	-2.699434	4.501466	-2.892030
206	1	0	-1.238808	-3.460176	-1.893288
207	1	0	0.227224	-2.904445	-1.750271

Prod’$_{phenol}$

E(B3LYP*/LB)= -5130.783584   Number of imaginary frequencies: 13

Center Number	Atomic Number	Atomic Type	Coordinates (Angstroms) X	Y	Z
1	26	0	-2.475695	-2.655055	0.177367
2	26	0	-4.130989	0.326262	-0.376184
3	6	0	2.216675	-0.632971	-2.431682
4	16	0	3.518732	0.340500	-3.174327
5	6	0	1.236008	0.090498	-1.581605
6	6	0	4.534134	-1.026565	-3.848321
7	6	0	1.456557	1.362383	-1.046197
8	6	0	5.413261	-1.691186	-2.775315
9	6	0	0.472240	1.930603	-0.226165
10	7	0	6.025567	-2.913262	-3.260296
11	8	0	2.123423	-1.845274	-2.623751
12	6	0	-0.712959	1.262657	0.063781
13	6	0	7.251818	-2.905984	-3.861609
14	8	0	7.913976	-1.880850	-4.006064
15	6	0	-0.968533	-0.016897	-0.496157
16	6	0	7.747250	-4.269565	-4.337706
17	6	0	0.035751	-0.588962	-1.299132
18	6	0	9.229220	-4.467466	-4.007027
19	6	0	6.344618	-4.907027	0.267397
20	6	0	5.731031	-6.261423	-0.096388
21	6	0	4.216699	-6.172041	-0.350644
22	6	0	3.901886	-5.221859	-1.500504
23	8	0	4.441731	-5.350487	-2.601950
24	7	0	2.986609	-4.248098	-1.246081
25	6	0	4.554989	-0.571956	3.482994
26	6	0	3.122685	-0.665063	3.979292
27	8	0	2.369010	0.312931	3.937047
28	6	0	4.584583	-0.546697	1.931437
29	8	0	4.090303	-1.791915	1.418690
30	6	0	6.001960	-0.359553	1.408513
31	7	0	2.686761	-1.892110	4.379119
32	6	0	1.277654	-2.161370	4.693170
33	6	0	0.898654	-3.594086	4.334486
34	6	0	1.189854	-3.902527	2.842410
35	6	0	0.410444	-3.045056	1.858043
36	8	0	-0.807662	-3.059557	1.758515
37	8	0	1.196622	-2.292576	1.076440
38	6	0	0.125181	3.059393	5.757676
39	6	0	-0.325808	2.280368	4.518861
40	8	0	-0.334976	0.867808	4.732494
41	6	0	-3.623018	-3.196089	4.321821
42	6	0	-2.734654	-1.941046	4.362761
43	6	0	-3.294284	-0.671202	3.733477
44	6	0	-3.620345	-0.738033	2.257648
45	8	0	-3.642893	-1.871382	1.653269
46	8	0	-3.917413	0.346170	1.689866
47	6	0	-1.732885	-7.475024	3.027548
48	6	0	-1.722294	-6.162366	2.217391
49	6	0	-2.963340	-5.951073	1.386411
50	7	0	-3.227745	-4.801150	0.634832
51	6	0	-3.997435	-6.836294	1.209328

52	6	0	-4.384373	-5.012952	0.028196
53	7	0	-4.887827	-6.227638	0.346589
54	6	0	6.036097	5.631511	4.314280
55	6	0	6.358040	4.154942	4.025046
56	6	0	5.976268	3.760215	2.614845
57	6	0	4.670955	3.344068	2.316842
58	6	0	6.897700	3.865902	1.563176
59	6	0	4.294484	3.058405	1.003348
60	6	0	6.526683	3.574342	0.249281
61	6	0	5.220134	3.173218	-0.036474
62	6	0	-2.231177	8.960290	-1.266465
63	6	0	-1.989318	8.995272	0.259031
64	6	0	-1.071977	7.933810	0.831614
65	6	0	0.241439	8.252447	1.205051
66	6	0	-1.523567	6.624082	1.050641
67	6	0	1.073048	7.300434	1.796625
68	6	0	-0.698255	5.663823	1.642009
69	6	0	0.603040	6.004301	2.021579
70	6	0	0.705618	5.935047	-3.735488
71	6	0	0.211332	4.488825	-3.919636
72	8	0	0.853395	3.664557	-4.566231
73	6	0	0.895946	6.428522	-2.292793
74	8	0	1.098892	7.844708	-2.281535
75	6	0	2.038524	5.697138	-1.588642
76	6	0	-8.427868	1.511665	-2.754172
77	6	0	-7.166319	1.281166	-1.923718
78	6	0	-7.363276	0.341920	-0.773943
79	7	0	-6.297815	-0.060621	0.015949
80	6	0	-8.512836	-0.230537	-0.288089
81	6	0	-6.788081	-0.852563	0.951422
82	7	0	-8.129058	-0.988105	0.804996
83	7	0	-1.028937	4.202441	-3.412272
84	6	0	-1.591159	2.860319	-3.546291
85	6	0	-3.076402	2.814072	-3.167098
86	6	0	-3.311234	3.008485	-1.666122
87	8	0	-2.826735	3.987098	-1.084270
88	8	0	-4.043230	2.113196	-1.071105
89	6	0	-6.368044	-2.808305	-4.240741
90	6	0	-4.865787	-3.039077	-4.567023
91	6	0	-3.866079	-1.992908	-4.044480
92	6	0	-3.861431	-1.890422	-2.522118
93	8	0	-3.351843	-2.849678	-1.835473
94	8	0	-4.406568	-0.875218	-2.028851
95	8	0	-2.900018	3.162933	1.775685
96	8	0	-1.009879	-3.836754	-1.205843
97	8	0	-2.116686	-0.657554	-0.295739
98	1	0	5.909049	-6.990430	0.703253
99	1	0	6.207512	-6.654785	-1.000338
100	1	0	3.825339	-7.157677	-0.629908
101	1	0	3.693114	-5.862947	0.562734
102	1	0	2.783721	-3.984828	-0.293736
103	1	0	2.831520	-3.528778	-1.947851
104	1	0	6.280211	-4.210253	-0.574361
105	1	0	3.954935	0.281454	1.584881

106	1	0	5.996651	-0.349514	0.315872
107	1	0	6.643784	-1.183763	1.739022
108	1	0	6.426323	0.584916	1.759611
109	1	0	3.127831	-1.735342	1.346108
110	1	0	4.998157	0.344773	3.879504
111	1	0	-0.163021	-3.746216	4.547125
112	1	0	1.453213	-4.319589	4.942219
113	1	0	0.912161	-4.940350	2.633282
114	1	0	2.254834	-3.783805	2.631405
115	1	0	0.695000	-1.480167	4.061647
116	1	0	-1.335457	2.575772	4.216379
117	1	0	0.336609	2.523087	3.675861
118	1	0	0.583815	0.583114	4.582283
119	1	0	0.415134	4.078074	5.470739
120	1	0	-2.507157	-1.697379	5.407473
121	1	0	-1.776520	-2.170884	3.888685
122	1	0	-4.215321	-0.356715	4.244819
123	1	0	-2.578118	0.146432	3.871870
124	1	0	-3.560615	-3.728549	3.374761
125	1	0	-1.601150	-5.304584	2.886248
126	1	0	-0.854420	-6.148972	1.544787
127	1	0	-4.171974	-7.821666	1.609058
128	1	0	-5.750076	-6.619883	0.001094
129	1	0	-4.855749	-4.324996	-0.657559
130	1	0	-1.855556	-8.339946	2.366120
131	1	0	5.825009	3.522004	4.745141
132	1	0	7.428839	3.978336	4.182890
133	1	0	7.916157	4.182330	1.777809
134	1	0	3.942910	3.235045	3.117633
135	1	0	7.257308	3.659011	-0.550378
136	1	0	3.277150	2.740601	0.797701
137	1	0	4.929866	2.945492	-1.058133
138	1	0	4.966632	5.828549	4.188649
139	1	0	-2.962642	8.927886	0.762793
140	1	0	-1.583001	9.980783	0.517925
141	1	0	0.610535	9.261725	1.039226
142	1	0	-2.536795	6.347537	0.770334
143	1	0	2.083874	7.573700	2.089137
144	1	0	-1.090254	4.667114	1.822198
145	1	0	1.246115	5.266155	2.494719
146	1	0	-1.291235	9.026427	-1.821767
147	1	0	-0.025075	6.289458	-1.716960
148	1	0	2.984867	5.860909	-2.120787
149	1	0	1.857912	4.617342	-1.569121
150	1	0	2.142854	6.055084	-0.562894
151	1	0	1.935384	8.020031	-2.734496
152	1	0	1.657087	5.993381	-4.274078
153	1	0	-6.359546	0.890328	-2.553252
154	1	0	-6.792577	2.233846	-1.529826
155	1	0	-9.541017	-0.163334	-0.604138
156	1	0	-8.737588	-1.527278	1.400946
157	1	0	-6.213109	-1.359744	1.710765
158	1	0	-9.274710	1.801817	-2.121439
159	1	0	-3.496223	1.852320	-3.472369

160	1	0	-3.617258	3.603577	-3.704474
161	1	0	-1.034445	2.149064	-2.919338
162	1	0	3.846450	-1.748490	-4.292781
163	1	0	5.152061	-0.587895	-4.635003
164	1	0	2.375452	1.898159	-1.253604
165	1	0	4.817058	-1.923797	-1.888776
166	1	0	6.212923	-1.006465	-2.483159
167	1	0	0.629698	2.919111	0.196109
168	1	0	-1.455189	1.737305	0.700336
169	1	0	0.671780	-1.726592	0.455313
170	1	0	7.132065	-5.072000	-3.916359
171	1	0	7.607237	-4.299326	-5.426323
172	1	0	-0.167364	-1.523677	-1.804086
173	1	0	9.613206	-5.387946	-4.457431
174	1	0	9.387035	-4.528730	-2.925054
175	1	0	9.809161	-3.620801	-4.381274
176	1	0	-6.507931	-2.251772	-3.309208
177	1	0	-4.737460	-3.129915	-5.651541
178	1	0	-4.562193	-4.005876	-4.147561
179	1	0	-4.095997	-1.001106	-4.442564
180	1	0	-2.859576	-2.271565	-4.378542
181	1	0	-3.036459	3.453830	0.854182
182	1	0	-3.362799	2.314464	1.847219
183	1	0	7.399815	-4.992093	0.528108
184	1	0	5.826919	-4.440714	1.114313
185	1	0	5.167217	-1.429218	3.762769
186	1	0	1.069842	-1.806313	5.702450
187	1	0	-0.814582	-7.639914	3.591233
188	1	0	-2.562628	-7.483857	3.742410
189	1	0	-3.284574	-3.822678	5.147362
190	1	0	-4.675374	-2.952205	4.513689
191	1	0	-6.751514	-3.819190	-4.102313
192	1	0	-6.908456	-2.299487	-5.041574
193	1	0	-8.717681	0.617123	-3.315336
194	1	0	-8.272015	2.328240	-3.459089
195	1	0	-1.446801	2.520999	-4.572106
196	1	0	-2.751695	8.046656	-1.570938
197	1	0	-2.859575	9.819820	-1.501113
198	1	0	-0.012383	6.567679	-4.257414
199	1	0	6.575470	6.289309	3.624940
200	1	0	6.312756	5.907669	5.331919
201	1	0	0.999692	2.580957	6.211671
202	1	0	-0.652579	3.163562	6.514335
203	1	0	3.362371	-2.637851	4.456145
204	1	0	5.489256	-3.776894	-3.204832
205	1	0	-1.429060	4.764338	-2.668816
206	1	0	-1.683574	-3.730655	-1.908628
207	1	0	-0.173543	-3.516041	-1.571629
